# Detecting tropical peatland degradation: Combining remote sensing and organic geochemistry

**DOI:** 10.1371/journal.pone.0280187

**Published:** 2023-03-29

**Authors:** Chloe Brown, Doreen S. Boyd, Sofie Sjögersten, Christopher H. Vane

**Affiliations:** 1 School of Geography, University of Nottingham, Nottingham, United Kingdom; 2 School of Biosciences, University of Nottingham, Nottingham, United Kingdom; 3 British Geological Survey, Centre for Environmental Geochemistry, Keyworth, United Kingdom; Assam University, INDIA

## Abstract

Tropical peatlands are important carbon stores that are vulnerable to drainage and conversion to agriculture. Protection and restoration of peatlands are increasingly recognised as key nature based solutions that can be implemented as part of climate change mitigation. Identification of peatland areas that are important for protection and restauration with regards to the state of their carbon stocks, are therefore vital for policy makers. In this paper we combined organic geochemical analysis by Rock-Eval (6) pyrolysis of peat collected from sites with different land management history and optical remote sensing products to assess if remotely sensed data could be used to predict peat conditions and carbon storage. The study used the North Selangor Peat Swamp forest, Malaysia, as the model system. Across the sampling sites the carbon stocks in the below ground peat was ca 12 times higher than the forest (median carbon stock held in ground vegetation 114.70 Mg ha^-1^ and peat soil 1401.51 Mg ha^-1^). Peat core sub-samples and litter collected from Fire Affected, Disturbed Forest, and Managed Recovery locations (i.e. disturbed sites) had different decomposition profiles than Central Forest sites. The Rock-Eval pyrolysis of the upper peat profiles showed that surface peat layers at Fire Affected, Disturbed Forest, and Managed Recovery locations had lower immature organic matter index (I-index) values (average I-index range in upper section 0.15 to -0.06) and higher refractory organic matter index (R -index) (average R-index range in upper section 0.51 to 0.65) compared to Central Forest sites indicating enhanced decomposition of the surface peat. In the top 50 cm section of the peat profile, carbon stocks were negatively related to the normalised burns ratio (NBR) (a satellite derived parameter) (Spearman’s rho = -0.664, S = 366, p-value = <0.05) while there was a positive relationship between the hydrogen index and the normalised burns ratio profile (Spearman’s rho = 0.7, S = 66, p-value = <0.05) suggesting that this remotely sensed product is able to detect degradation of peat in the upper peat profile. We conclude that the NBR can be used to identify degraded peatland areas and to support identification of areas for conversation and restoration.

## 1. Introduction

Tropical peat swamp forests are recognised for their global importance as carbon sequestering and storing ecosystems, and their role for climate change mitigation [1,2]. Under stable conditions, lowland tropical peatlands have the potential to form one of the most efficient carbon capture environments, with peat accumulation rates up to ten times faster in the tropics compared with other peatlands (temperate, subarctic, boreal) [3–6]. However, increased climate variability, amplified by degradation associated with land use change and resource management, has the potential to shift tropical peatlands from a net carbon sink to a source [7–10]. South-East Asia has experienced the most rapid and destructive large-scale peatland degradation. Since the 1980’s, widespread degradation through deforestation [11–13], drainage [7,8] and frequent intense fire events [13–15], have dramatically increased the pressure on peat swamp forests in South-East Asia.

Ombrotrophic tropical peat swamp forests are rainwater-fed ecosystems with environmental and topographic conditions favouring poor drainage, oversaturation and substrate acidification [16]. The peat deposits are formed and maintained by the continuous input of organic matter from evergreen swamp forest species under waterlogged conditions [17,18]. The surplus of water on the peatland floor creates an intermittent lack of oxygen, thus restricting microbial decomposition creating optimum conditions for peat accumulation, in particular during waterlogged conditions [19]. The microtopography of tropical peatlands creates an irregular forest floor, with elevated surfaces, hummocks and hollows [20,21]. When the organic matter inputs in the peat soil undergo microbial mediated aerobic and anaerobic decomposition greenhouse gasses (carbon dioxide (CO_2_), methane (CH_4_) and nitrous oxide (N_2_O)) can be released into the atmosphere [22–25]. Tropical peatland degradation accelerates these processes releasing high levels of greenhouse gas emissions into the atmosphere through vegetation removal, peat oxidation and combustion [7,10,24–26].

Remote sensing has become one of the primary data sources for large-scale tropical peat swamp forest assessment [27]. This provides a rapid and economical approach to supplement traditional direct field-based methods such as coring and probing [28,29]. Typically, field-based measurements (with laboratory analyses) are used to determine peat properties (for example—peat thickness, bulk density, carbon content), with remote sensing data used to derive information on the area extent, land use change, fire events and vegetation cover [30–32]. Remote sensing data can be collected via a number of platforms such as planes, UAV’s, kites and satellites; in this paper we focus on data captured by satellites. Space-borne data offers a low cost (often freely available) data solution, with regular resampling intervals and large area coverage. Additionally, some satellite series offer archival data, for example the Landsat series dates from 1972. Due to the sheer extent and inaccessibility often associated with tropical peat swamp forests, remote sensing has been flagged as a key technology in tropical peatland mapping and assessment, particularly for monitoring programs.

Biological, physical and geochemical analyses of in situ-samples remains the foundation of peatland soil research. Field measurements are required to develop an accurate, quantitative view on the impact of present-day surface disturbance and land management practices on the underlying peat soil profile formed centuries to millennium ago. Rock-Eval (6) pyrolysis has been identified as a low-cost organic geochemistry method for quantifying soil carbon and tracking soil organic matter (SOM) thermal stability in soil horizons [33–36]. This method has been previously used to investigate carbon dynamics in a wide range of environments—recently on tropical peats [25,37,38], freshwater and saltmarsh peats [39–41] and marine sediments [42]. A better understanding of tropical peat organic geochemistry dynamics and reactions to disturbance (natural and anthropogenic) is vital for targeting resources and regeneration activities in sustainable peatland ecosystem management.

The aim of this study therefore is to explore the use of remote sensing and organic geochemistry for investigating the impact of tropical peat swamp forest degradation on the underlying peat soil profile. In view of this, the research addressed the following questions: (1) What are the relative contributions of above ground vegetation and peat soil to carbon stocks under different peat swamp forest condition classes? (2) What are the impacts of disturbance and past land management on the organic geochemistry throughout the peat profile and (3) Can remote sensing and GIS derived products predict peat soil carbon stocks and properties associated with degradation? The research addressed these questions by combining remote sensing and GIS data with geochemical analysis of peat cores collected across multiple sites under different land management in North Selangor Peat Swamp Forest (NSPSF) reserve, Malaysia.

## 2. Materials and methods

The study gained ethical approval from the School of Geography, University of Nottingham ethics committee for the research.

### 2.1 Study site

NSPSF (3°33’31 N, 101°18’21 E) is one of the largest remaining tropical ombrotrophic peat swamps in Peninsular Malaysia [43]. Comprised of four forest reserves—Raja Musa Forest Reserve, Sungai Karang Forest Reserve, Sungai Dusun Wildlife Reserve and part of Bukit Belata Forest Reserve Extension–NSPSF covers a total area of 81,304 ha. The reserve is predominantly covered by secondary mixed swamp forest; the majority of the area has been selectively logged from the 19th century up until the 1980’s and as a result forest condition varies throughout the reserve. Additionally, the extraction of timber led to the formation of a 500 km network of drainage canals [44], altering the forest structure and accelerating the drainage and erosion of peat soil stores (and the carbon they hold) [43]. In response to past peat swamp forest degradation key areas of the reserve are under restoration and regeneration programmes, such as the updated Integrated Management Plan (IMP) [44] prepared by the Global Environment Centre (GEC).

A significant area of the northern edge of the reserve has already undergone oil palm conversion which has impacted strongly on peat properties and function [25,45]. Reserve encroachment and the hydrological consequences of adjacent oil palm plantation drainage pose a serious threat to the protection and ecosystem function of NSPSF. NSPSF also boarders the Tanjung Karang Irrigation Scheme to the west which predominantly serves the Sekinchan rice paddy fields and mine/ ex-mining activities to the south-east [44].

During the early Holocene, NSPSF would have been colonized by mangrove species later to be replaced with fresh water peat swamp forest vegetation after the last Holocene interglacial marine incursion. This has resulted in the deposit of acidic peat overlaying grey marine clay [46]. Past studies have recorded an average peat depth of 3.6 m across the reserve [44], with the deepest peat depth recorded in Sungai Karang Forest Reserve at 10.15 m. Typical to tropical peat swamp forest, the peat soil in NSPSF is rich in woody materials, including tree trunks, branches and roots, especially in the upper 50 cm section of the peat profile. The forest floor is covered by large intertwined tree root mats, with a 2–3 cm thick loose layer of fallen leaf litter. Tree basal area were 21.58 ± 3.99 m^2^ ha^-1^, tree stem density were 286.66 ± 22.05 stems ha^-1^. During the wet season the water table is close to the peat surface. Soil temperature during the wet season were 26.1 ± 0.52°C. Bulk density) were 0.10 ± 0.008 g cm^−3^, Organic matter content were 95.1 ± 0.99% and soil pH were 3.6 ± 0.06 (Cooper *et al*., 2020).

NSPSF’s tropical climate is characterised by heavy rainfall, high temperatures, and high humidity. The area receives an average annual rainfall of over 2,000 mm [46], with distinct peaks from March to April and October to November. NSPSF has an average recorded shaded air temperature of 28.5°C, with an average monthly relative humidity of 77.2% [47].

To investigate the impact of tropical peatland degradation on the underlying peat soil profile, four different peat swamp forest condition classes were identified to reflect the different forms and degrees of degradation (both natural and anthropogenic) (Table 1).

**Table 1:** Description of forest condition classes.

**Central Forest**	**Managed Recovery**
• tall dense forest, closed canopy • close proximity to river • subject to past selective logging activities, no targeted logging in approx. 40 years • limited human disturbance low fire risk	• tall/medium forest, closed canopy • close proximity to forest boundary • targeted forest regeneration management • subject to past selective logging activities • ecotourism zone low fire risk
**Disturbed Forest**	**Fire Affected**
• medium forest, open canopy • close proximity to forest boundary and encroachment of oil palm (active area of forest conversion) • adjacent to targeted drainage for oil palm agriculture • subject to past selective logging activities medium fire risk	• low forest and scrub vegetation • frequent large scale intense fire events • targeted forest regeneration management peat subsidence

### 2.2 Field sampling

Within each of the peat swamp forest condition classes peat cores were collected for the entire peat profile during a field campaign in August 2017 (dry season): Central Forest = 3 cores, Managed Recovery = 2 cores, Disturbed Forest = 4 cores, and Fire Affected = 2 cores. The complexity and non-uniform nature of tropical peat, for example variations in forest floor structure and depth, means that the peat samples should not be spatially extrapolated to represent large areas of the reserve, instead they are purely a representation of the peat and the processes that impact it for the defined sample area. For information on the location of each of these condition classes please refer to the Selangor State Forestry Department 2014, Integrated Management Plan for North Selangor Peat Swamp Forest 2014–2023.

The peat cores were extracted using a side-filling Russian Peat Corer (Van Walt, UK), with a 0.5 m long sampling chamber. The peat profile was sampled in 0.5 m increments, from the ground surface to the underlying mineral material (clay layer). Immediately following extraction the peat cores were split into 10 cm segments which were bagged and sealed in the field. Surface leaf litter material was collected and bagged separately at each of the sites. All peat and litter samples were transported to laboratories at the University of Nottingham Malaysia Campus for initial processing.

### 2.3 Sample processing

#### 2.3.1 Bulk density and carbon storage

To determine bulk density, all sub-samples from the peat profiles collected were weighed for wet weight, oven-dried at 40°C for 48 hours, and then re-weighed for dry weight. Bulk density was calculated as: oven dried mass (g cm^-3^) / volume (cm^3^). In this case each 10 cm section = 98.17 cm^3^ [45].

The carbon density for the peat sub-samples (g cm^-2^) were first calculated from the bulk density multiplied by the total organic carbon (TOC) (generated from Rock-Eval analysis), then multiplied by the depth per layer (10 cm). The carbon stock (Mg/cm^2^) for each peat core was then calculated as the sum of each 10 cm layer throughout the peat core. Mg/cm^2^ was then scaled to Mg/ha.

#### 2.3.2 Rock-Eval 6 pyrolysis

Rock–Eval (6) pyrolysis, is a well-established screening tool in petroleum geochemistry [48]. It has increasingly been used to track bulk changes in organic matter composition and the degree of decomposition [36,40], most recently in tropical peatlands [25,37,38].

The peat core sub-samples and leaf litter collected were analysed using Rock-Eval (6) pyrolysis. Samples for analyses were selected before and after obvious changes in texture and/or colour in the peat profile stratigraphy. Freeze-dried powdered peat and litter samples (~60 mg) were heated at 200°C for three minutes before an increase in temperature to 650°C at a rate of 25°C per minute in an inert N_2_ atmosphere. Residual carbon was subsequently oxidized from 300°C to 850°C at a rate of 20°C per minute. The amount of hydrocarbons released during the two-stage pyrolysis process was detected using a flame ionisation detector, CO and CO_2_ were measured using infrared detectors during the thermal cracking of the organic matter. Rock-Eval analysis generated a range of standard parameters, for this research we assessed the organic geochemistry of the peat cores using the following parameters:

- **S1**—a measure of free hydrocarbons released on heating to 200°C
- **S2**- hydrocarbons released on the thermal cracking of organic matter for temperatures up to 650°C
- **TOC (wt %)**—calculated from the sum of the carbon moieties (HC, CO, and CO_2_)
- **TpkS2**—the temperature associated with the highest yield of bound hydrocarbons
- **S3 CO** and **S3 CO**_**2**_—the CO and CO_2_ yielded from the breakdown of kerogen
- **HI (mg HC g TOC)**–the amount of bound hydrocarbons released relative to the TOC
- **OI (mg O**_**2**_ **g TOC)**—corresponds to the quantity of oxygen released as CO and CO_2_ relative to TOC

The intrinsic organic matter (OM) stability of each peat sample was analysed through the calculation of the refractory OM index (R-index) and immature OM index (I-index) proposed by Sebag *et al*., [33]. These indices are calculated from the deconvolution of S2 pyrograms using the temperature nodes at 200–340°C (A1), 340–400°C (A2) and 400–460°C (A3) and >460°C (A4). Nodes A1 and A2 are taken to broadly correspond to labile fresh plant mater and lignin and cellulose respectively. The more thermally stable A3 and A4 nodes correspond to highly humified macromolecule and mature recalcitrant soil organic matter, or charcoal respectively [44,49].

The R-index is calculated using the higher temperature areas representing more thermally refractory OM compounds:

R‐index=(A3+A4/100)

The I-index is calculated using the lower temperature areas representing thermally more labile OM compounds:

I‐index=Log10((A1+A2)/A3)

### 2.4 Remote sensing data

A satellite time-series, captured by Landsat Thematic Mapper (TM) and Operational Land Imager (OLI), was utilised in this research to investigate forest condition and disturbance in NSPSF over the time period 1989–2019. Data selection for this time period (dry season) sought to find data scenes with a relatively cloud free view across the entire study area. Landsat data was acquired for the years 1989, 1995, 2001, 2010 and 2019.

For each Landsat scene, Surface Reflectance (SR) of six spectral bands (blue, green, red, near infrared (NIR) and 2x shortwave infrared (SWIR-1 and SWIR-2)) were downloaded at a spatial resolution of 30 m. The SR products were provided by USGS Earth Resources Observation and Science (EROS) Center Science Processing Architecture (ESPA) (https://espa.cr.usgs.gov); Landsat 5 data was generated by the Landsat Ecosystem Disturbance Adaptive Processing System (LEDAPS) [50] and Landsat 8 data was generated by the Landsat Surface Reflectance Code (LaSRC) [51].

The Normalised Burn Ratio (NBR) was calculated for each of the Landsat scenes from the normalised reflectance of NIR and SWIR bands: NBR = (NIR–SWIR-2)/(NIR+SWIR-2). NBR spectral index ranges between 1 and − 1, with low values indicating low or no vegetation present and high values representative of dense healthy vegetation. In the past NBR has been successfully used to measure burn severity of fires [52], detect and classify forest disturbances [53], and assess forest attributes [54,55].

The NBR results for each year were stacked and simple descriptive statistics calculated for the time-series. A raster layer of average NBR was calculated from the NBR time-series stack to gauge a recent (relative to the peat profile) view of vegetation cover and condition in NSPSF. The standard deviation (sd) for the pixels in the NBR time-series stack was calculated; the data was proven to have a normal distribution (Shapiro–Wilk test) therefore the resulting standard deviation raster layer can be used as a measure of pixel variability over time. Low variability is expected in areas with low disturbance (natural and anthropogenic). Conversely high variability is expected in areas routinely impacted by repeated disturbance such as: large-scale fire events, repeated drainage, and forest encroachment. All processing was generated in R software (version 3.5.2) using the ‘Raster’ package [56].

### 2.5 Additional geo-spatial data

In addition, the proximity to the river (Sungai Tengi) running though the reserve and the proximity to drainage canals were assessed to help determine the impact of human management and the hydrology on the peat soil profiles. Shapefiles for the river and canal network were provided by the GEC IMP report [44]. Distance measurements (m) were calculated in ArcGIS 10.2.2. Estimates for carbon stored in the AGB (Mg/ha) for NSPSF was calculated by applying a carbon content conversion factor of 0.47 [57] to the AGB estimates generated in the research presented in Brown *et al*., [58].

### 2.6 Data analysis

All statistical analysis was conducted in R software (version 3.5.2). A variety of non-parametric statistical tests were employed in this research. Spearman’s rank correlation coefficient was employed to test for correlation between Rock-Eval parameters and indices with remote sensing and GIS derived products. Wilcoxon test was used to determine if there were statistically significant differences between the carbon stocks (Mg ha^-1^) held in the above ground vegetation and the peat soil. The Kruskal-Wallis test and Pairwise Wilcoxon test (with Bonferroni adjustment to correct for familywise error in the Kruskal-Wallis test) were used to determine if there were statistically significant differences between Rock-Eval parameters and indices between forest condition classes and peat depth layers (surface peat layer, peat just below the water table, and the deepest peat layers within each core which was above the underlying clay layer). The relationships between Rock-Eval parameters (HI and OI) and indices (R-index and I-index) were plotted for visually assessment of degradation patterns linked to these parameters.

## 3. Results

### 3.1 Carbon stock

**Fig 1:**
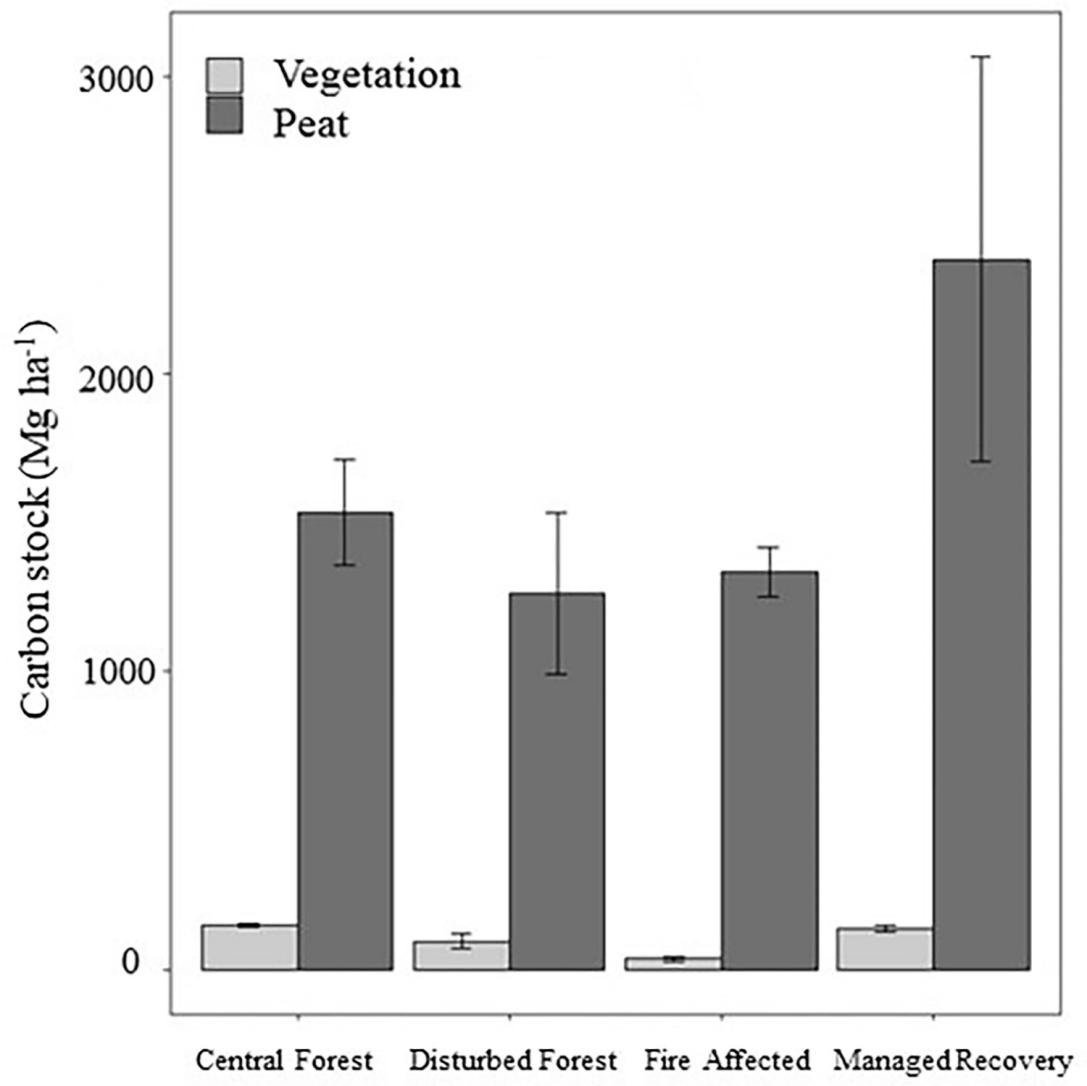
Carbon storage in the vegetation and peat soil for each of the four forest condition classes, mean and error bars (standard error of the mean) shown.

Carbon stocks were found to be significantly different between above ground vegetation and peat soil (W = 121, d.f. = 1, p-value = <0.001), with 12.2 times more carbon stock held in peat than in the vegetation (Fig 1, Table 2). The peat carbon stock did not differ significantly between the four different forest condition classes. Peat carbon stocks were linked to peat depth, which ranged from 0.5–3.5 m in the peat cores sampled.

**Table 2:** Median carbon stock (Mg ha^-1^) and interquartile range (IQR) for above ground vegetation and peat soil. © University of Nottingham/ British Geological Survey © UKRI.

	N	Median	IQR
**Vegetation**	11	114.70	70.48
**Peat**	11	1401.51	336.76

### 3.2 Rock-Eval indices and parameters

**Fig 2:**
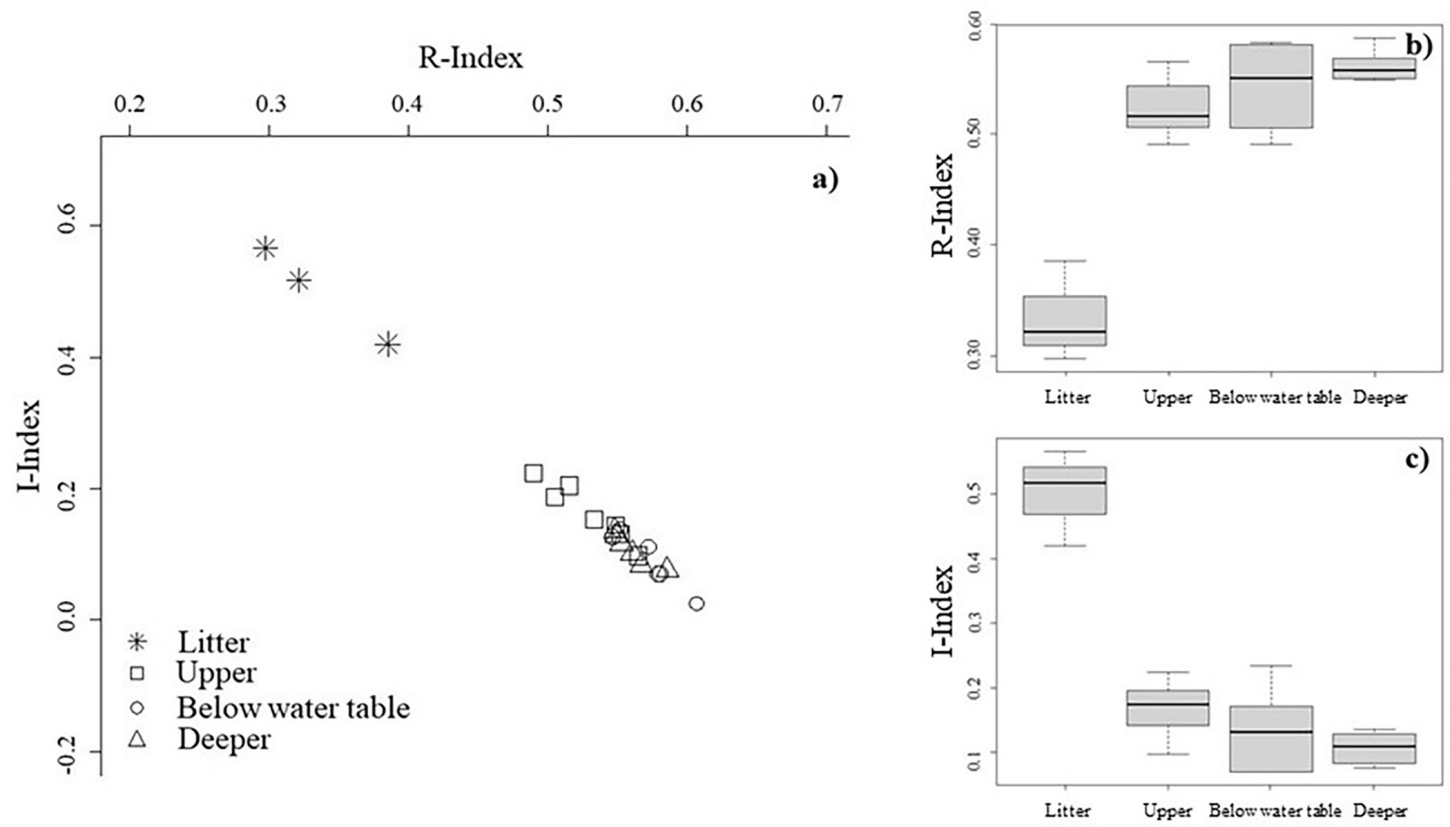
Rock-Eval indices for Central Forest sites: a) I/R diagram; b) boxplots of R-index values for peat profile layers; c) boxplots of I-index values for peat profile layers.

Trends in peat decomposition can be followed on an I-index vs R-index diagram (I/R diagram). Peat core sub-samples and litter collected from Central Forest locations (Fig 2) illustrate the expected (natural) decomposition trend found in a stable, relatively undisturbed tropical peat ecosystem. The I/R diagram showed a decrease in the proportions of immature labile carbon (average I-index range 0.18 to 0.8) and an increase in the proportion of refractory carbon (average R-index range 0.51 to 0.57) with depth. This trend was comparable with Rock-Eval analysis of peats from pristine wetlands of Bocas del Toro, Panama (Supplementary material). There is some overlap in the below water table and deeper peat layers with intervals both above and below the general downward trend. It should be noted that these peat cores do not cover the deepest layers of peat found in NSPSF (2–3 m depth) therefore samples labelled as below the water table and at deeper layers were relatively close together within these peat profiles.

**Fig 3:**
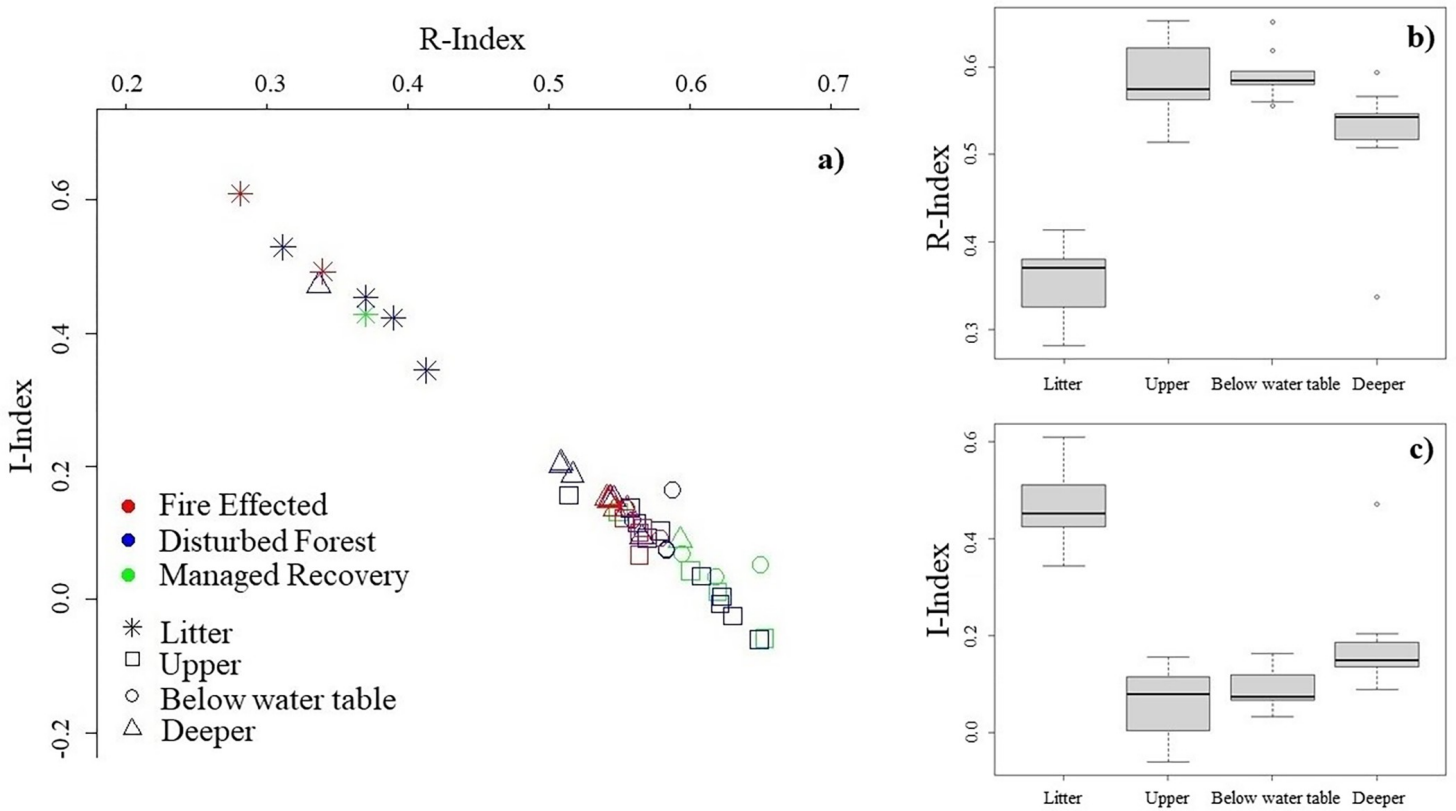
Rock-Eval indices for disturbed sites: a) I/R diagram; b) boxplots of R-index values for peat profile layers; c) boxplots of I-index values for peat profile layers.

Peat core sub-samples and litter collected from Fire Affected, Disturbed Forest, and Managed Recovery locations (i.e. disturbed sites) (Fig 3) do not follow the same decomposition trend found in the Central Forest sites. The upper peat intervals on average produce lower I-index values (average I-index range in upper section 0.15 to -0.06) and higher R -index (average R-index range in upper section 0.51 to 0.65) than the deeper intervals of the peat profile (i.e more thermally stable).

Based on the Rock-Eval I- and R- indices the effects of peat core location and disturbance were assessed between the different depth layers in the peat profiles. For the upper 50 cm of the peat profile, R-index and I-index values were found to be significantly different among locations (R-index: Kruskal-Wallis chi-squared = 11.196, d.f. = 3, p-value = <0.05; I-index: Kruskal-Wallis chi-squared = 10.693, d.f. = 3, p-value = <0.05)(Table 3). A significant difference in R-index and I-index values were found between Central Forest locations and disturbed sites (R-index: W = 11, d.f. = 1, p-value = <0.001; I-index: W = 115, d.f. = 1, p-value = <0.001)(Table 4).

**Table 3:** R- and I- index median and interquartile range (IQR) for upper 50 cm section in the peat profile for the four forest condition classes. Significant pairwise Wilcoxon tests with Bonferroni adjustments share the same subscript letter. © University of Nottingham/ British Geological Survey © UKRI.

Forest Condition Class	N	R-index		I-index	
		median	IQR	median	IQR
Central Forest	3	0.52^a^	0.04	0.17^b^	0.05
Disturbed Forest	4	0.59^a^	0.06	0.06^b^	0.12
Fire Affected	2	0.56	0.01	0.10	0.02
Managed Recovery	2	0.61	0.04	0.03	0.07

**Table 4:** R- and I- index median and interquartile range (IQR) for upper 50 cm section in the peat profile for Central Forest and disturbed sites. © University of Nottingham/ British Geological Survey © UKRI.

	N	R-index		I-index	
		median	IQR	median	IQR
Central Forest	3	0.52	0.04	0.17	0.05
disturbed	8	0.57	0.06	0.08	0.11

For the combined below water table and deeper sections of the peat profile, R-index and I-index values were found to be significantly different between locations (R-index: Kruskal-Wallis chi-squared = 12.79, d.f. = 3, p-value = <0.001; I-index: Kruskal-Wallis chi-squared = 13.907, d.f. = 3, p-value = <0.001) (Table 5). No significant difference in R-index and I-index values were found between Central Forest locations and disturbed sites.

**Table 5:** R- and I- index median and interquartile range (IQR) for combined below water table and deeper layers for the four forest condition classes. Significant pairwise Wilcoxon tests with Bonferroni adjustments share the same subscript letter. © University of Nottingham/ British Geological Survey © UKRI.

Forest Condition Class	N	R-index		I-index	
		median	IQR	median	IQR
Central Forest	3	0.56^a^	0.03	0.11^d^	0.05
Disturbed Forest	4	0.55^b^	0.06	0.15^e^	0.09
Fire Affected	2	0.55^c^	0.01	0.14^df^	0.02
Managed Recovery	2	0.61^abc^	0.03	0.06^ef^	0.03

**Fig 4:**
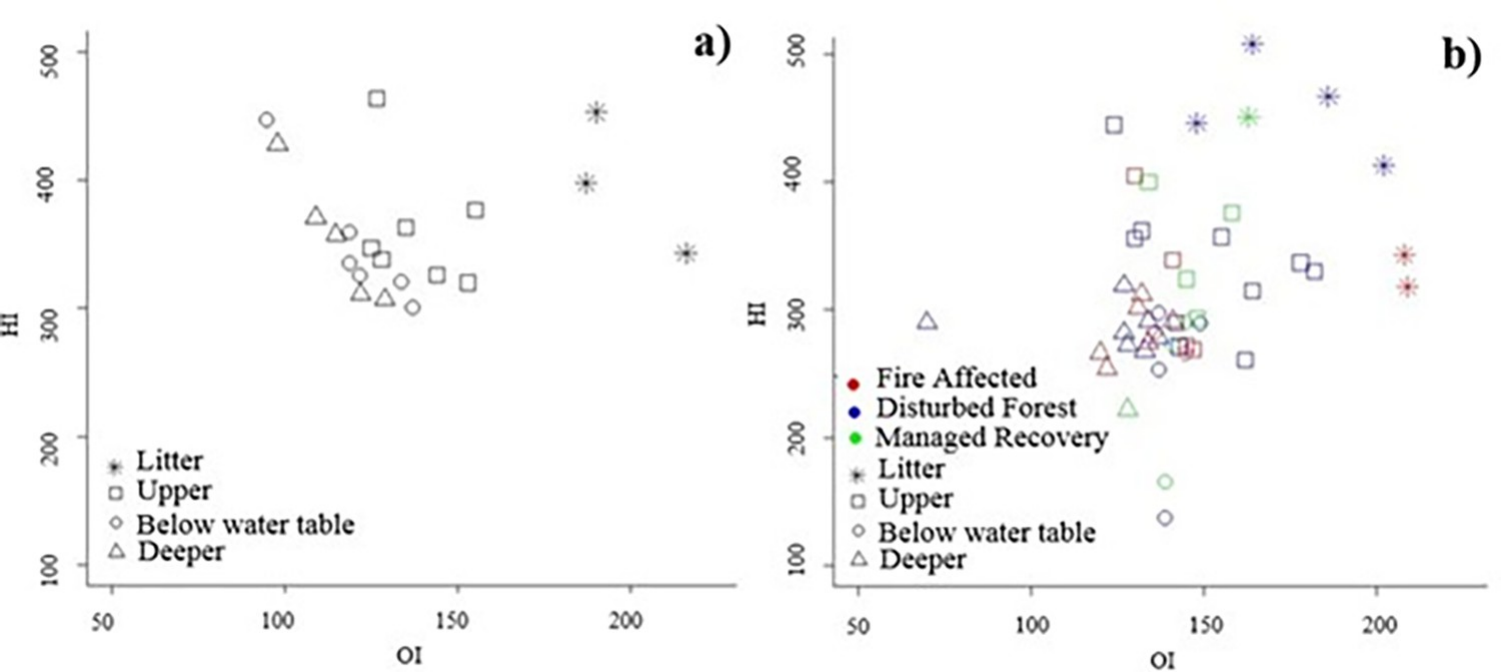
Van Krevelen pseudo diagrams. (a) Central Forest; (b) disturbed sites.

The HI vs OI diagram for Central Forest sites showed that HI values remained high through the peat profile (300–475 mg HC g _TOC_^-1^) but that OI values continuously decreased from the leaf litter down profile to the deeper peat layers (Fig 4A). The high HI values throughout the whole peat profile (≥300 HC g _TOC-1_) indicate a high concentration of plant tissue and other material rich in polysaccharides with a greater degree of hydrogenation and mineral transformation. For the disturbed sites (Fig 4B), OI and HI decreased with depth from leaf litter down profile to the deeper peat layers. This reflects the degradation of plant material and an increased humification of SOM, including greater aromaticity and dehydrogenation of organic compounds (loss of H).

### 3.3 Remote sensing and GIS

**Fig 5:**
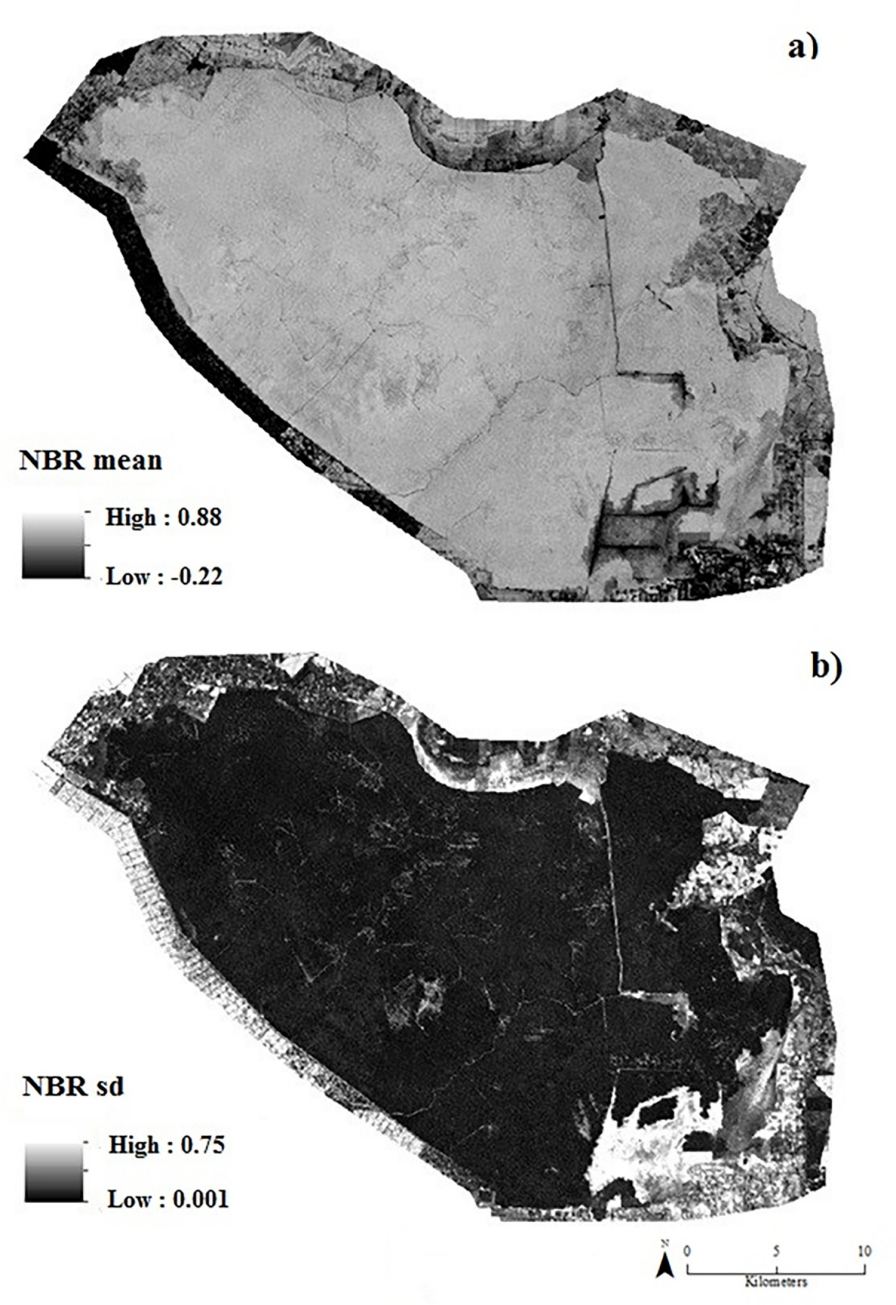
Remote sensing derived products: (a) Normalised Burn Ratio (NBR) mean; (b) Normalised Burn Ratio (NBR) standard deviation (sd).

The Landsat derived raster layers of average and standard deviation (sd) NBR time-series stack (representative of the years 1989, 1995, 2001, 2010 and 2019) show considerable variability across the reserve Fig 5. Fig 5A. provides a view of vegetation cover and condition in NSPSF. Fig 5B. represents a measure of pixel variability over time, this marking change in both peat swamp forest disturbance and recovery. Low variability indicates areas of low disturbance (natural and anthropogenic) and high variability indicates areas that have been routinely impacted by repeated disturbance during the time period 1989–2019.

**Fig 6:**
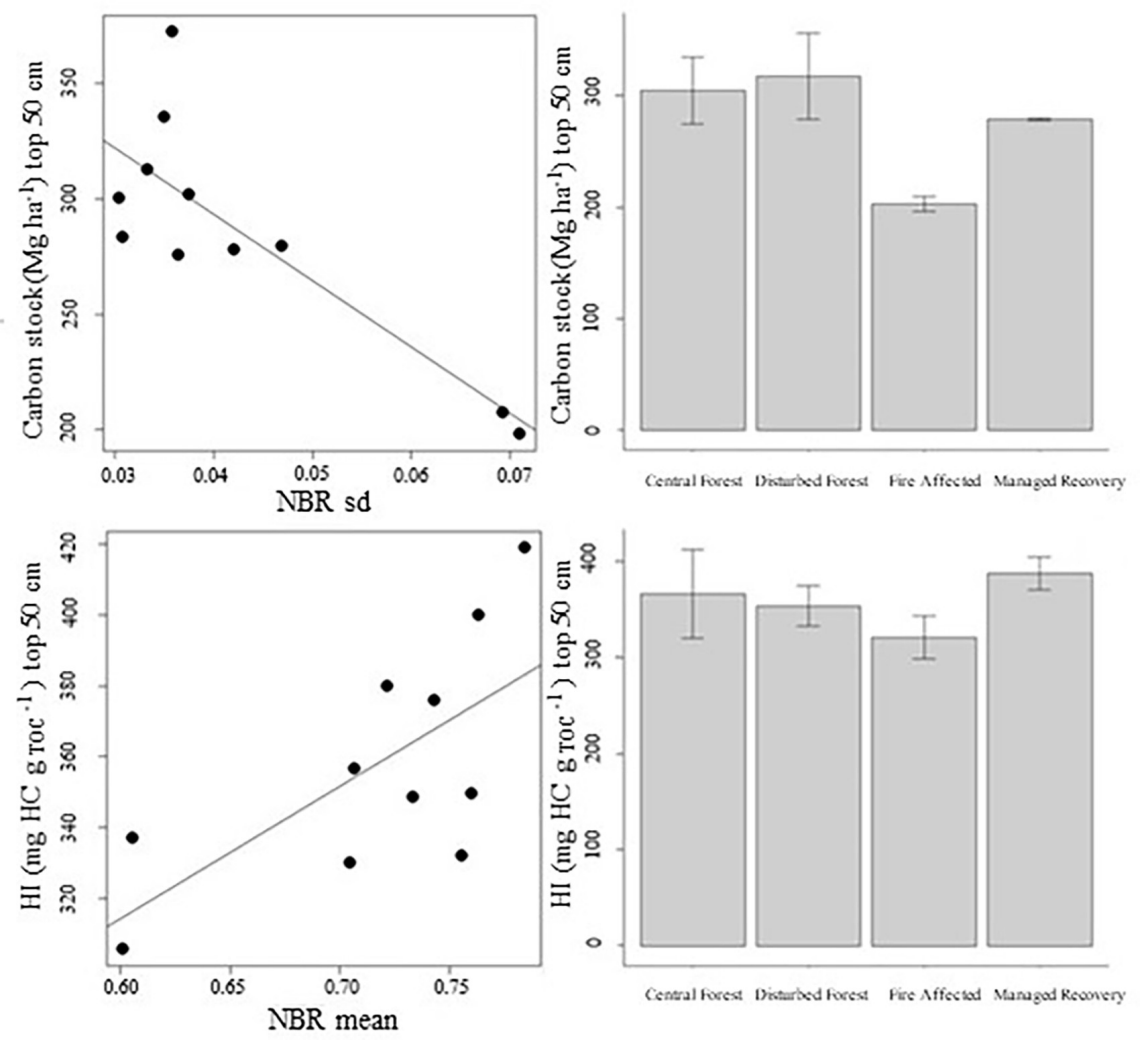
Significant relationships between remote sensing derived products and Rock-Eval pyrolysis parameters: (top left) correlation with Normalised Burn Ration Standard deviation (sd) and Carbon Stock held in the top 50 cm; (top right) Carbon stock held in top 50 cm mean values and SEM for the four forest condition classes; (bottom left) correlation with Normalised Burn Ration mean and HI for the top 50 cm; (bottom right) HI for the top 50 cm mean values and SEM for the four forest condition classes.

Peat carbon stock and the parameters generated from Rock-Eval analyses representative of the total peat profile were found to have no significant correlation with remote sensing and GIS derived products. However, in the top 50 cm section of the peat profile, carbon stocks were found to have a significant negative correlation with NBR sd (Spearman’s rho = -0.664, S = 366, p-value = <0.05) (Fig 6A). HI (the amount of hydrogen relative to the amount of organic carbon) and NBR mean were also found to have a significant positive correlation in the top 50 cm section of the peat profile (Spearman’s rho = 0.7, S = 66, p-value = <0.05) (Fig 6C).

## 4. Discussion

### 4.1 Carbon stocks

Tropical peat swamp forests combine the carbon storage potential in tropical forests with high biomass productivity and the capacity to create and store long-term carbon deposits in the underlying peat soil. However, there is a substantial difference between the magnitude of carbon stocks held by the vegetation and peat soil. Peat soil carbon stocks dwarf the carbon stored in the above ground vegetation in NSPSF (Table 2), which is an important feature in active, accumulative tropical peatlands [29,59,60]. Sites holding the highest carbon stock in the peat soil were found to also have the highest AGB (Managed Recovery and Central Forest sites). A healthy, productive AGB layer protects and promotes the underlying peat soil, creating a continuous supply of litter and organic material to incorporate into the waterlogged peat over prolonged periods [17]. Previous ecological studies have explored the positive relationship between vegetation cover and peat soil; AGB, species richness and plant functional type have been found to relate to the degree of water-logging and peat mineralization in boreal and sub-arctic peatlands [61,62]. To secure a productive and functional peatland ecosystem, there is an urgent need to conserve and protect both the above ground and below ground carbon stores.

### 4.2 Tropical peat decomposition trends

Based on the I/R diagrams (Figs 2 and 3), the dynamics of SOM can be assessed within the different peat profile depth layers (top 50 cm section, water table, and deeper) and leaf litter, or by comparing the peat profiles from the various forest condition classes and disturbance (i.e. Central Forest compared to disturbed sites). The R- and I-index have proven to be powerful indictors of SOM degradation [33,63]. Peat profiles collected in Central Forest sites follow a general negative linear relationship between I- and R- index with depth. This reflects the relationship between decay processes and SOM stabilisation. However, disturbed sites (Fire Affected, Disturbed Forest, Managed Recovery) do not follow this uniform pattern. This difference in the I/R diagrams is most apparent in the top 50 cm section of the peat profiles. For disturbed sites, samples taken from the top 50 cm section of the profile have a high R-index (0.514 to 0.653) and low I-index (0.156 to -0.061) and typically mark the tail-end values on the I/R diagram, indicating that the peat surface and subsurface layers have experienced a rapid depletion of the most thermally labile compounds in this layer, leaving thermally stable compounds behind [33]. In contrast, the Central Forest sites have a relatively lower R-index (0.490 to 0.565) and higher I-index (0.235 to -0.097) vales, reflective of an environment characterised by a continuous supply of fresh labile substrates [64] in anaerobic conditions. The relationship found between disturbed sites and greater thermal stability in the top 50 cm section has previously been explored by Cooper *et al*., [25] with forest conversion to mature oil palm.

In the deeper peat layers, Central Forest and disturbed sites generally hold similar positions in the I/R diagrams, suggesting that the deeper underlying peat in NSPSF has the same composition across all forest condition classes. In the disturbed sites (Fig 3), a number of deeper samples are plotted with very low R-index and high I-index values relative to their position in the peat profile. This is likely due to marine clay banding in the peat samples. Clay particles have been reported to create organo-mineral complexes which protect the organic matter from microbial decomposition [65], hence the preservation of organic material in deeper layers.

Peat core depth in this research ranged from 0.5–3.5 m. These depths are below the average recorded peat depth of 3.6 m across the reserve [44]. This difference could be due to the impact of peat coring activities being restricted to forest edge (range of 0.5–1 km in) and river edge locations, or the result of different peat depth measurement techniques in past research. The assessment of the thermal stability of SOM in deep peat layers is restricted in this research by the relatively limited deep peat representation in the samples.

### 4.3 Application of remote sensing in tropical peat soil degradation

In this research, no significant correlation was found between remote sensing and GIS derived products with peat carbon stock and Rock-Eval parameters representative of the total peat profile. The spectral data and analysis methods adopted in this research (Fig 5) were ultimately limited by optical remote sensing data’s inability to penetrate and record past the ground surface [66]. As has been previously discussed, a relationship exists between vegetation cover and the underlying peat soil in some tropical peatland ecosystems [18], however, it is not representative of the entire peat profile and cannot demonstrate destabilisation of peat throughout the peat profile.

Importantly, a significant correlation was found between remote sensing derived data (NBR sd and carbon stock; NBR mean and HI (Fig 5)) in the top 50 cm section of the peat profile. This suggests that remote sensing products can predict carbon stocks and peat degradation parameters in the surface layers of tropical peat. The surface (0–30 cm) and sub-surface (30 cm-water table) layers represent a dynamic environment in the peat profile linked to fluctuations in the water table. These layers are the interface of a number of active chemical and biological processes in tropical peat [67,68]. These processes are influenced by changes in the water table position (both seasonal and as a consequence of disturbance), which then determines the potential depth of oxygen availability in peat. In oxygen rich conditions, decomposition is enhanced and thus nutrient availability due to mineralization increases. Surface and subsurface peat has been recognized as significant contributors to greenhouse gas production in tropical peatlands [19,69,70]; land-use change induced deforestation, drainage and fire are all linked to accelerating degradation and the release of greenhouse gases [25,71–73]. These land-use changes and disturbances can be mapped and monitored by remote sensing data [12,30,74,75].

In view of the strengths and limitations of remote sensing, we suggest the application of remote sensing coupled with systematic field and laboratory measurements for the assessment of tropical peat soil carbon stock and degradation. This complementary, targeted approach has the potential to provide detailed field-based information of peat profiles in key locations, while at the same time allowing for spatially explicit assessments of peat degradation patterns and its severity over time.

## 5. Conclusions

Active and targeted tropical peat swamp forest management is essential in order to restore the carbon sink function of degraded tropical peatlands. Restoration efforts should aim to both reduce the carbon losses from the peat and increase carbon sequestration potential in the AGB. Remote sensing derived products have proven to be a useful tool in ecosystem restoration programmes, however field-based sampling and geochemical analyses remain an integral part in tropical peatland assessment.

We conclude that the NBR is able to predict carbon stock and condition in the upper 50 cm section of the peat profile. This is important as it demonstrates that remote sensing can be used to identify areas of potentially high carbon stock in the surface peat layers. Such information is urgently needed to target conservation efforts and identify areas of degradation that should be the focus for restoration activities.

## 6. Supplementary materials

**Fig 7:**
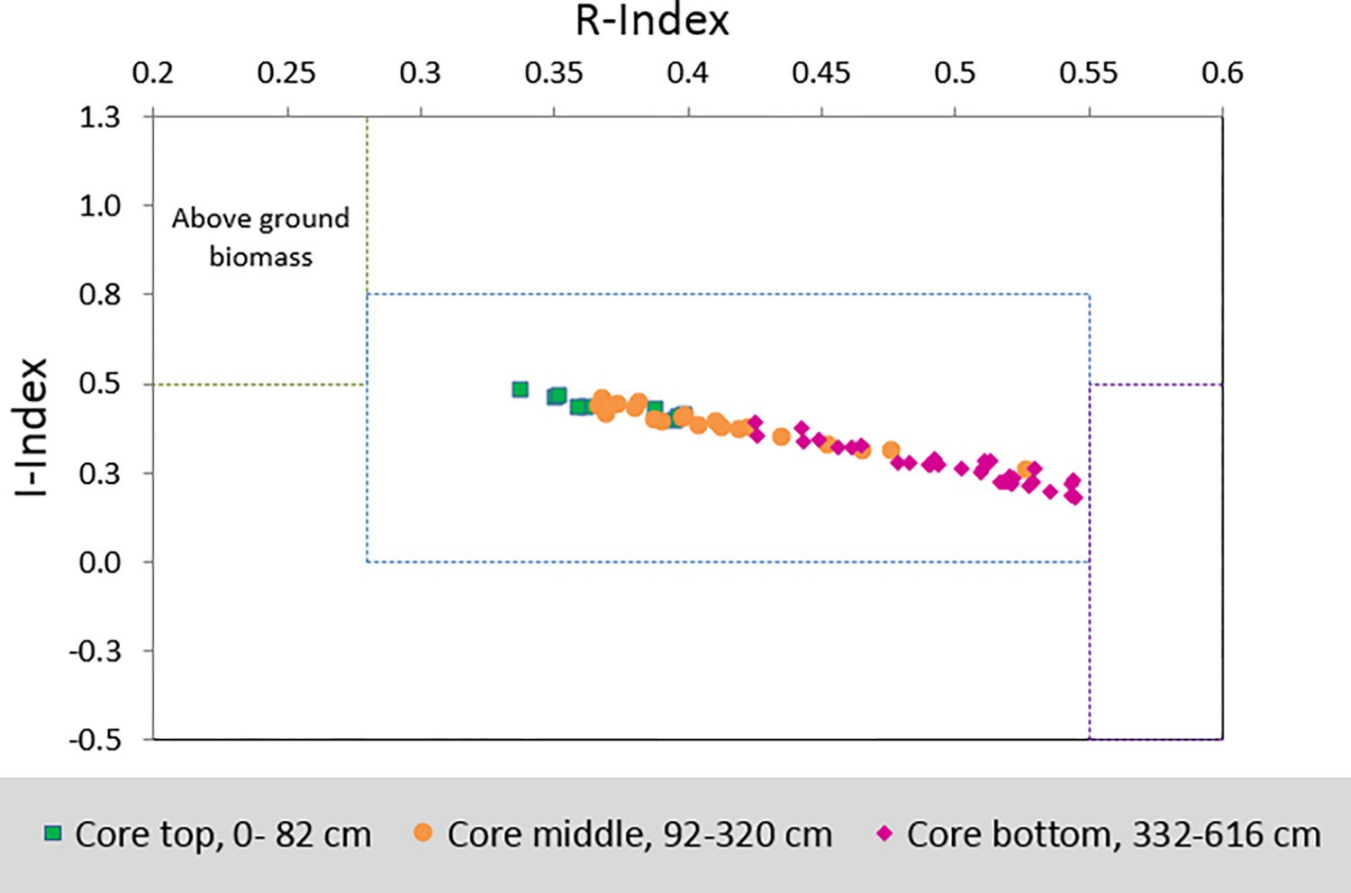
I/R diagram for mangrove peat core from Bocas Del Toro Panama (source data from [38]).

NSPSF has experienced multiple forms of anthropogenic disturbance, in order to better understand the decomposition trends afforded by Rock-Eval 6 pyrolysis a peat core sampled from a pristine peatland in Bocas del Toro, Panama (source data from [38]) was processed as a control dataset. The same methodology as outlined in this paper was applied to the Panama peat core in order to show the expected (natural) decomposition trend (Fig 7). Important to not here that the above ground vegetation was a mangrove forest not a tropical peat swamp forest. Inspection of the I/R diagram clearly shows a systematic decrease in the proportions of immature labile carbons (I-index 0.48 to 0.22) and increase in the proportion of refractory carbon (R-index 0.34 to 0.54) with depth. Although there is some overlap of the middle section of the core (92–320 cm).

## Supporting information

S1 File (ZIP)

S2 File (ZIP)
