# Supplementary material for: Detecting tropical peatland degradation: Combining remote sensing and organic geochemistry
Source: PLoS One. 2023 Mar 29;18(3):e0280187. doi: 10.1371/journal.pone.0280187 (PMC10057786; doi:10.1371/journal.pone.0280187)
Supplement: S1 File — (ZIP) [file pone.0280187.s001.zip › Landsat1989_clip/p127r58_4t19890207.html]

p127r58\_4t19890207

*UFM v1.4*

```

```

**Inventory Metadata**

**ECS Data Granule**

Size MB ECS Data Granule = 380.148000

Reprocessing Planned = no further update anticipated

Reprocessing Actual = processed once

Local Granule ID = p127r58\_4t19890207

Day Night Flag = Day

Production Datetime = 2001-05-16-T16:47:08.0Z

Local Version ID = 001

**Measured Parameter**

**Measured Parameter Container**

Parameter Name = Geospatial Image

**QA Statistics**

QA Percent Missing Data = 0

QA Percent Cloud Cover = 0

**Collection Description Class**

Short Name = GeoCvTM

Version ID = 1

**Input Granule**

Input Pointer = 1. p127r58\_4t19890207\_nn1.tif 2. p127r58\_4t19890207\_nn2.tif 3. p127r58\_4t19890207\_nn3.tif 4. p127r58\_4t19890207\_nn4.tif 5. p127r58\_4t19890207\_nn5.tif 6. p127r58\_4t19890207\_nn6.tif 7. p127r58\_4t19890207\_nn7.tif 8. p127r58\_4t19890207.met 9. p127r58\_4t19890207.hdr 10. p127r58\_4t19890207.jpg 11. p127r58\_4t19890207.htm

**Spatial Domain Container**

**Horizontal Spatial Domain Container**

**Zone Identifier Class**

Zone Identifier = 47

**G-Polygon**

G-Polygon 1

|  | Location | | |
| Sequence | Latitude | Longitude |
| Vertices | 0 | 3.788612 | 100.432672 |
| 1 | 3.784310 | 102.078952 |
| 2 | 2.244098 | 101.971725 |
| 3 | 2.246531 | 100.327388 |

**G-Ring**

Exclusion G-Ring Flag = N

**Single Datetime**

Time of Day = 02:44:00.0

Calendar Date = 1989-02-07

**Sensor Characteristic**

**Sensor Characteristic Container**

Platform Short Name = Landsat 4

Instrument Short Name = Landsat 4

Sensor Short Name = TM

**Additional Attributes**

**Additional Product Specific Attributes**

| Class | Name | Value |
| 1 | SolarAzimuth | 118.66 |
| 2 | SolarElevation | 49.95 |
| 3 | MaxBlockRMSE | 50 |
| 4 | Band1GainSetting | 0.6024314 |
| 5 | Band1BiasSetting | -1.5200000 |
| 6 | Band2GainSetting | 1.1750981 |
| 7 | Band2BiasSetting | -2.8399999 |
| 8 | Band3GainSetting | 0.8057647 |
| 9 | Band3BiasSetting | -1.1700000 |
| 10 | Band4GainSetting | 0.8145490 |
| 11 | Band4BiasSetting | -1.5100000 |
| 12 | Band5GainSetting | 0.1080784 |
| 13 | Band5BiasSetting | -0.3700000 |
| 14 | Band6GainSetting | 0.0551584 |
| 15 | Band6BiasSetting | 1.2377996 |
| 16 | Band7GainSetting | 0.0569804 |
| 17 | Band7BiasSetting | -0.1500000 |
| 18 | Landsat4WRSPath | 127 |
| 19 | Landsat4WRSRow | 58 |
| 20 | NumberofRows | 6914 |
| 21 | NumberofColumns | 7724 |
| 22 | ReferencePixelOrigin | Center of Reference Pixel |
| 23 | ReferencePixelRowNumber | 1 |
| 24 | ReferencePixelColumnNumber | 1 |
| 25 | PixelIncrementUnit | meters |
| 26 | ReferencePixelNorthing | 418921.50 |
| 27 | ReferencePixelEasting | 645069.00 |
| 28 | NorthingIncrement | -28.5 |
| 29 | EastingIncrement | 28.5 |
| 30 | RawSceneProvider | Not Available |
