# Supplementary figures and images for: Detecting tropical peatland degradation: Combining remote sensing and organic geochemistry
Source: PLoS One. 2023 Mar 29;18(3):e0280187. doi: 10.1371/journal.pone.0280187 (PMC10057786; doi:10.1371/journal.pone.0280187)

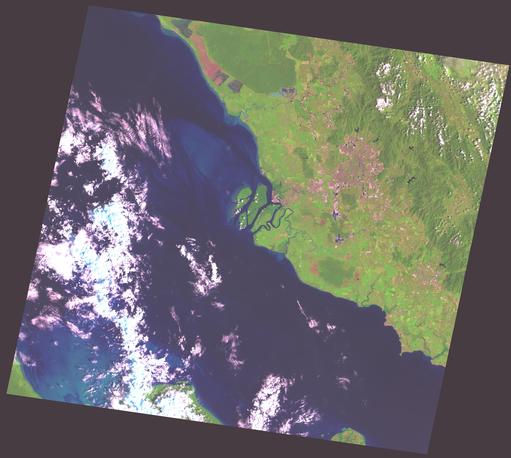

Supplement: S1 File — (ZIP) [file pone.0280187.s001.zip › Landsat1989_clip/p127r58_4t19890207.jpg]

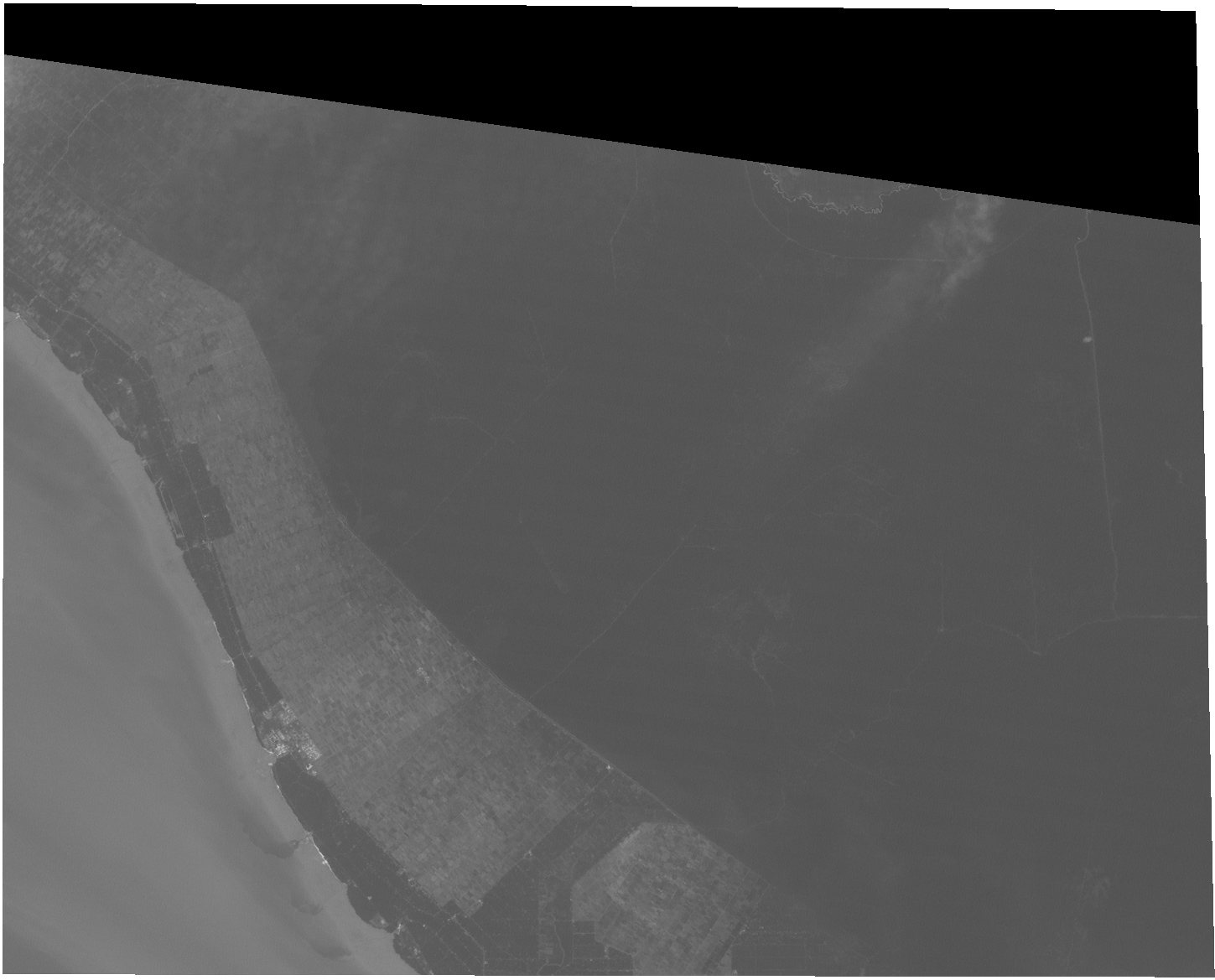

Supplement: S1 File — (ZIP) [file pone.0280187.s001.zip › Landsat1989_clip/p127r58_4t19890207_nn1.tif]

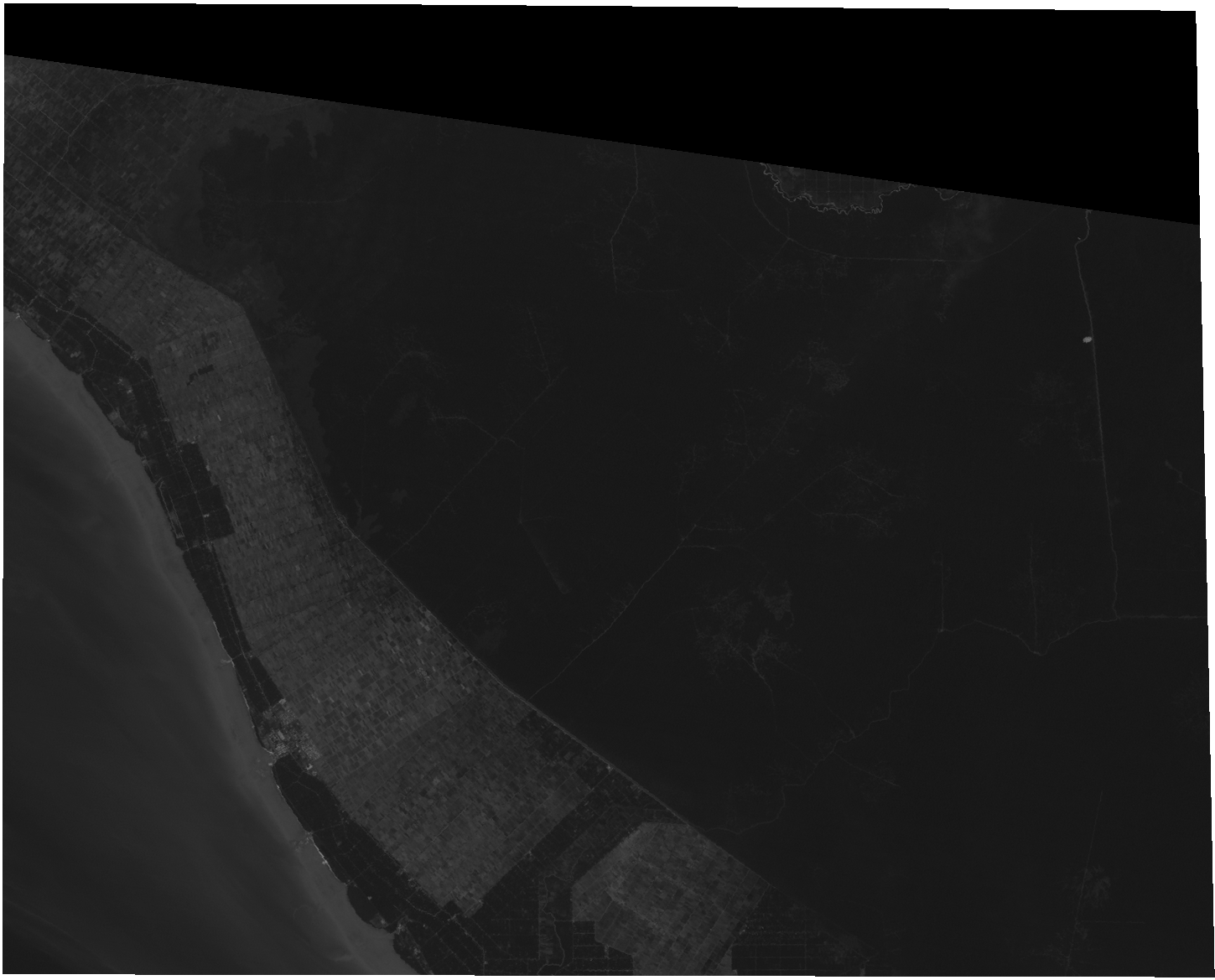

Supplement: S1 File — (ZIP) [file pone.0280187.s001.zip › Landsat1989_clip/p127r58_4t19890207_nn3.tif]

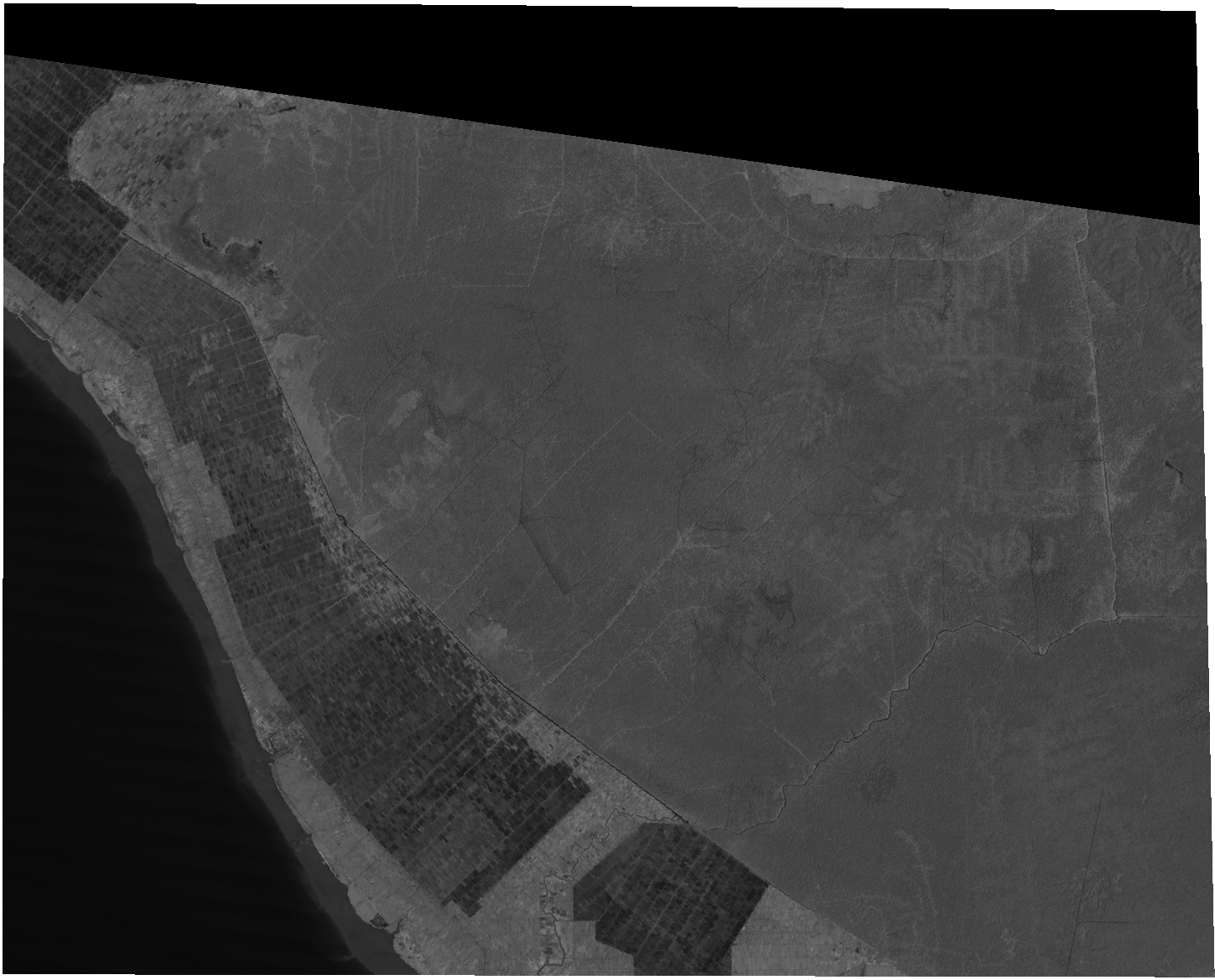

Supplement: S1 File — (ZIP) [file pone.0280187.s001.zip › Landsat1989_clip/p127r58_4t19890207_nn4.tif]

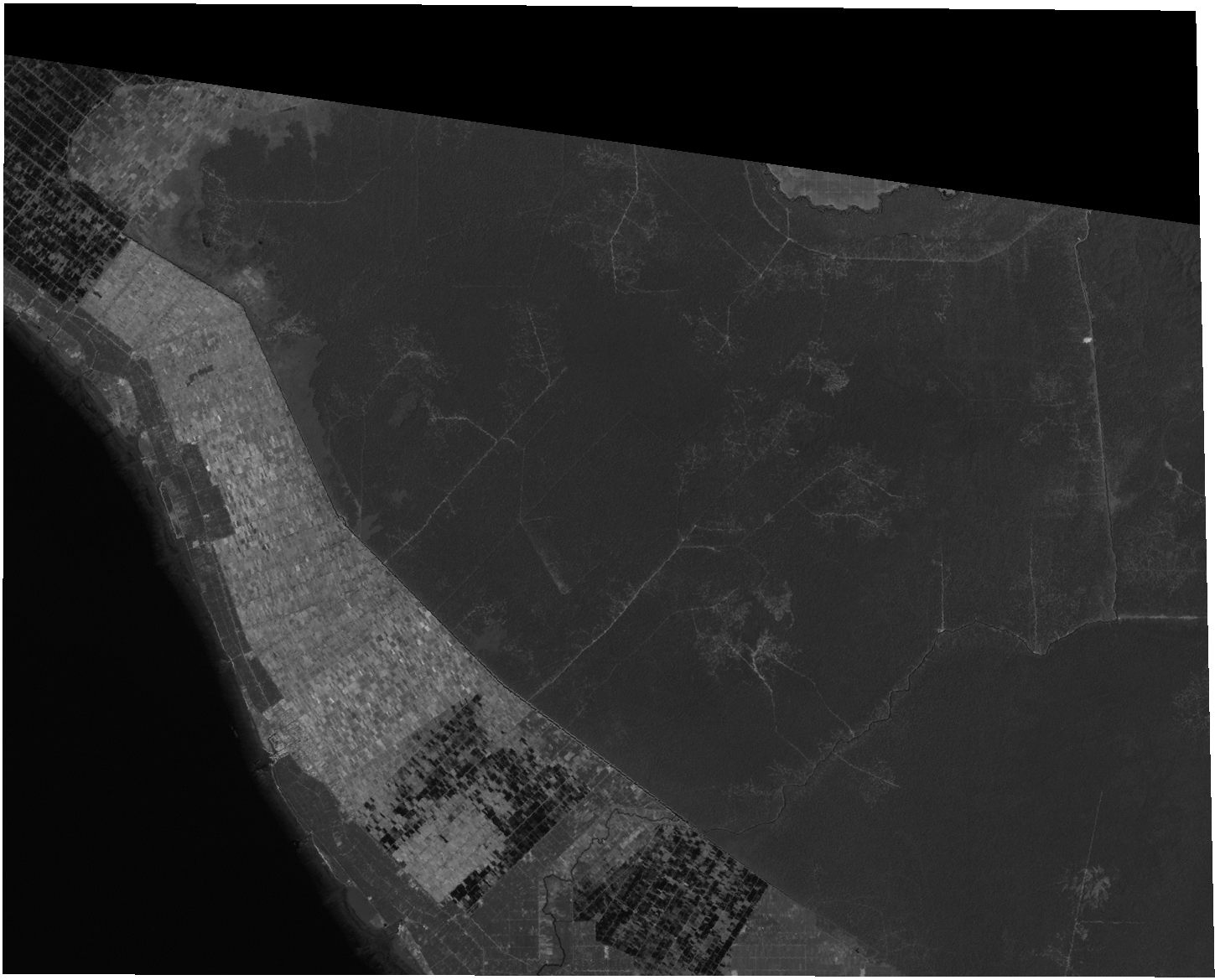

Supplement: S1 File — (ZIP) [file pone.0280187.s001.zip › Landsat1989_clip/p127r58_4t19890207_nn5.tif]

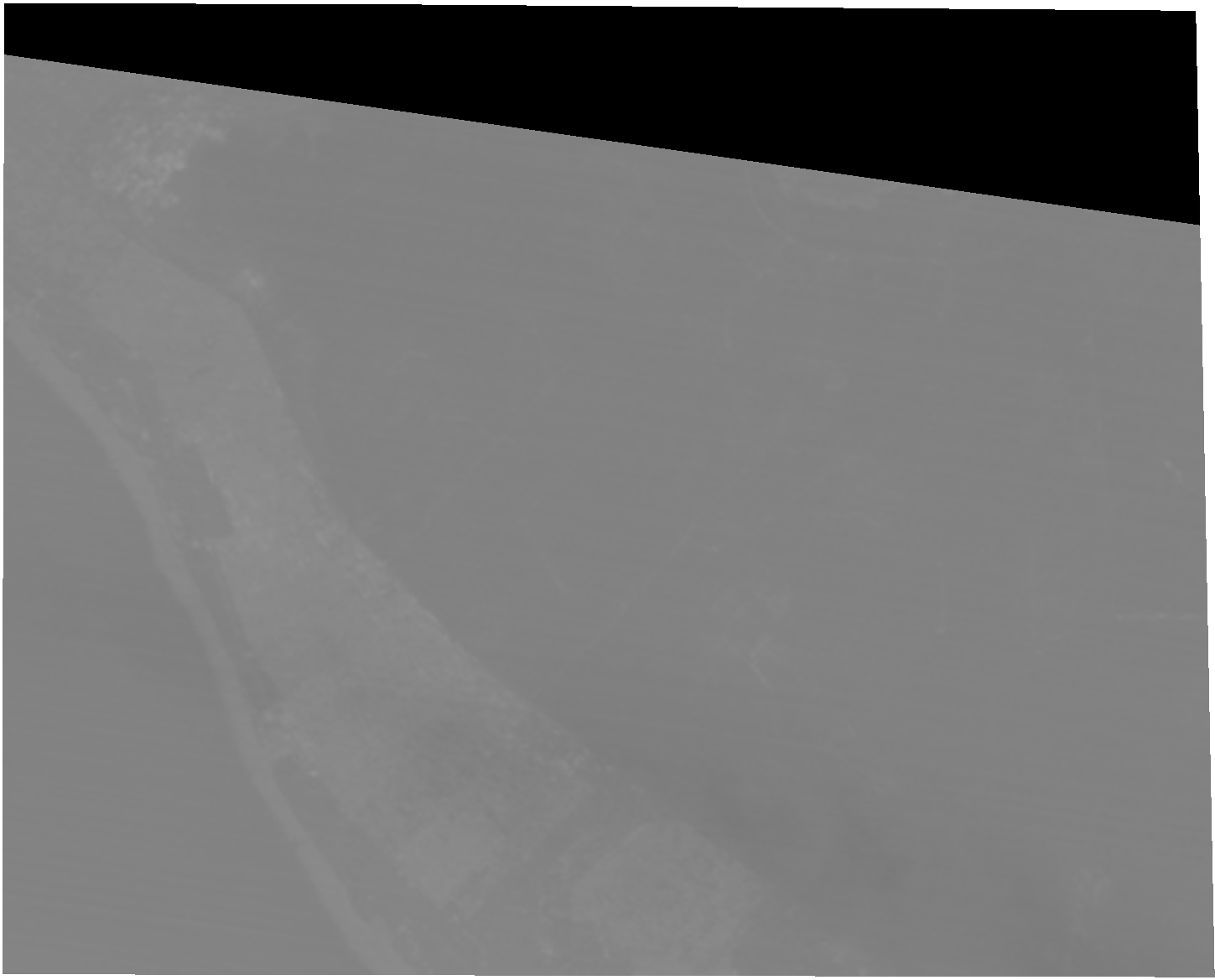

Supplement: S1 File — (ZIP) [file pone.0280187.s001.zip › Landsat1989_clip/p127r58_4t19890207_nn6.tif]

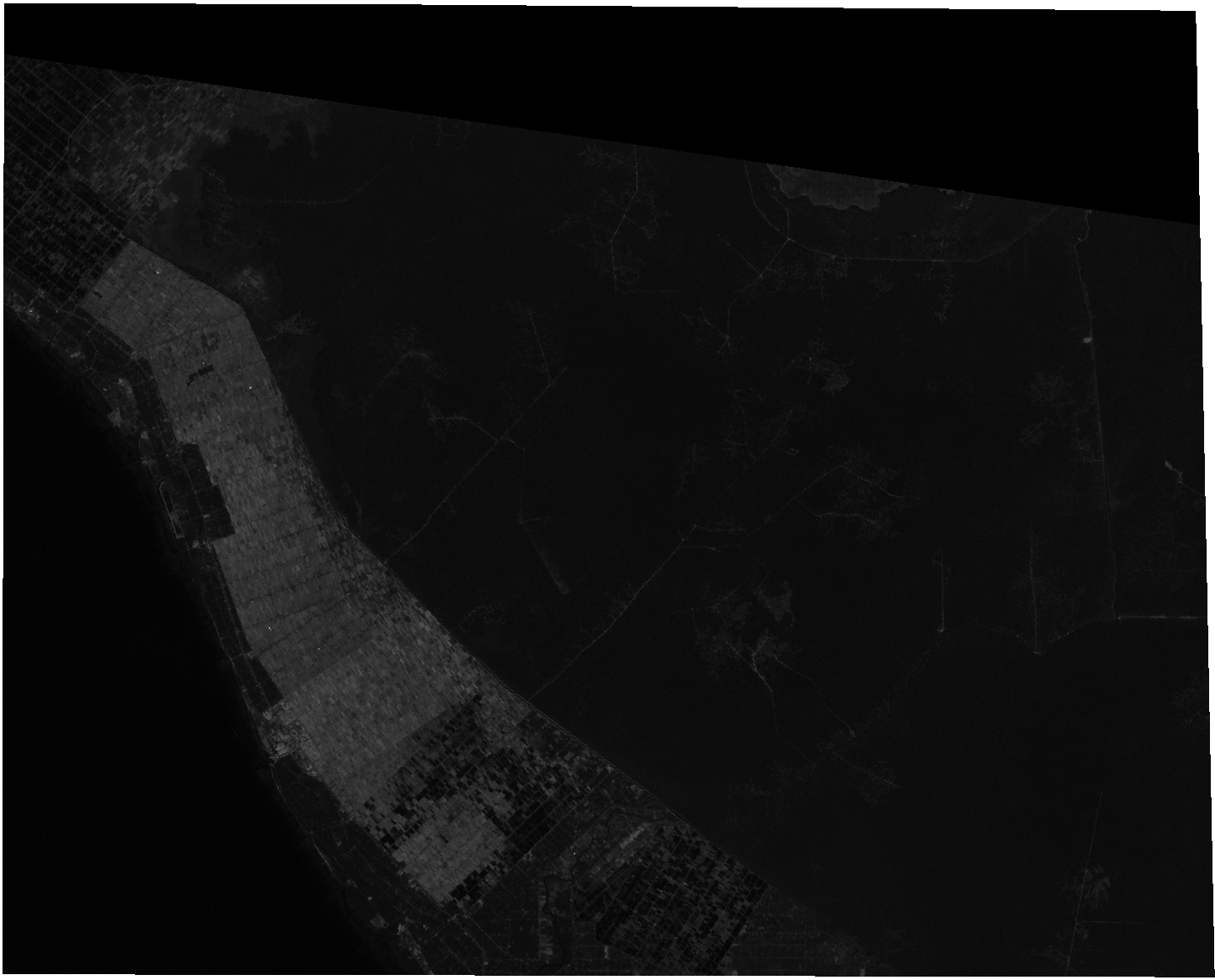

Supplement: S1 File — (ZIP) [file pone.0280187.s001.zip › Landsat1989_clip/p127r58_4t19890207_nn7.tif]

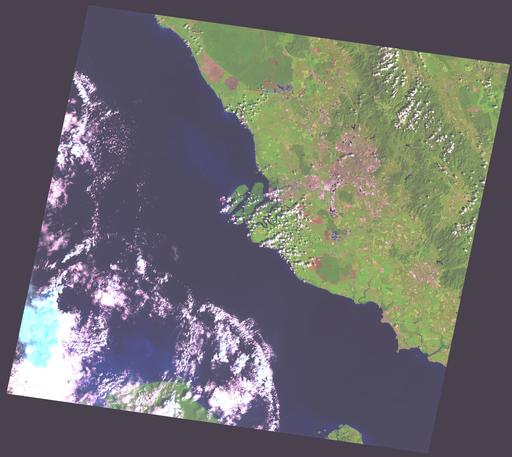

Supplement: S1 File — (ZIP) [file pone.0280187.s001.zip › Landsat1990_clip/p127r58_4t19901227.jpg]

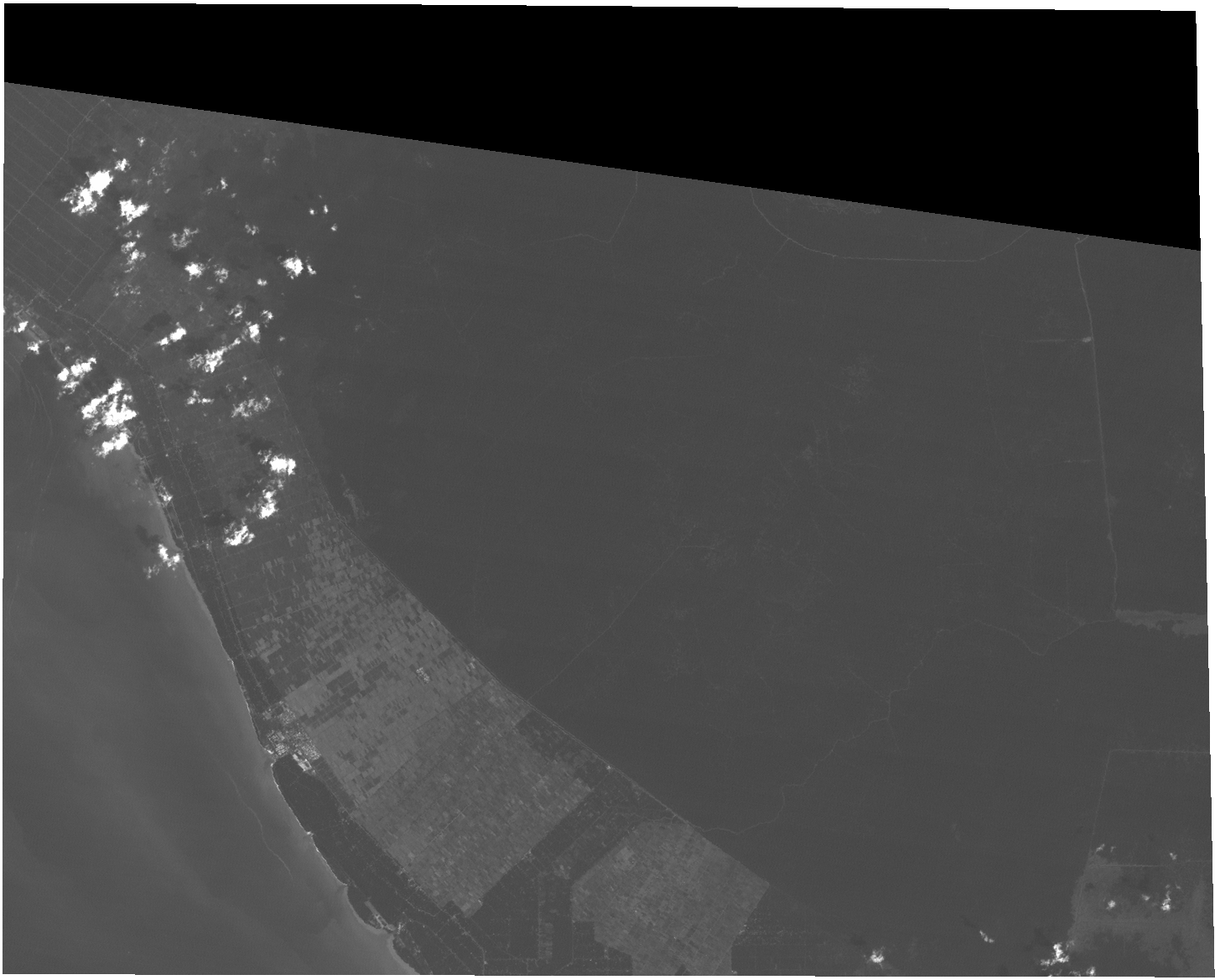

Supplement: S1 File — (ZIP) [file pone.0280187.s001.zip › Landsat1990_clip/p127r58_4t19901227_nn1.tif]

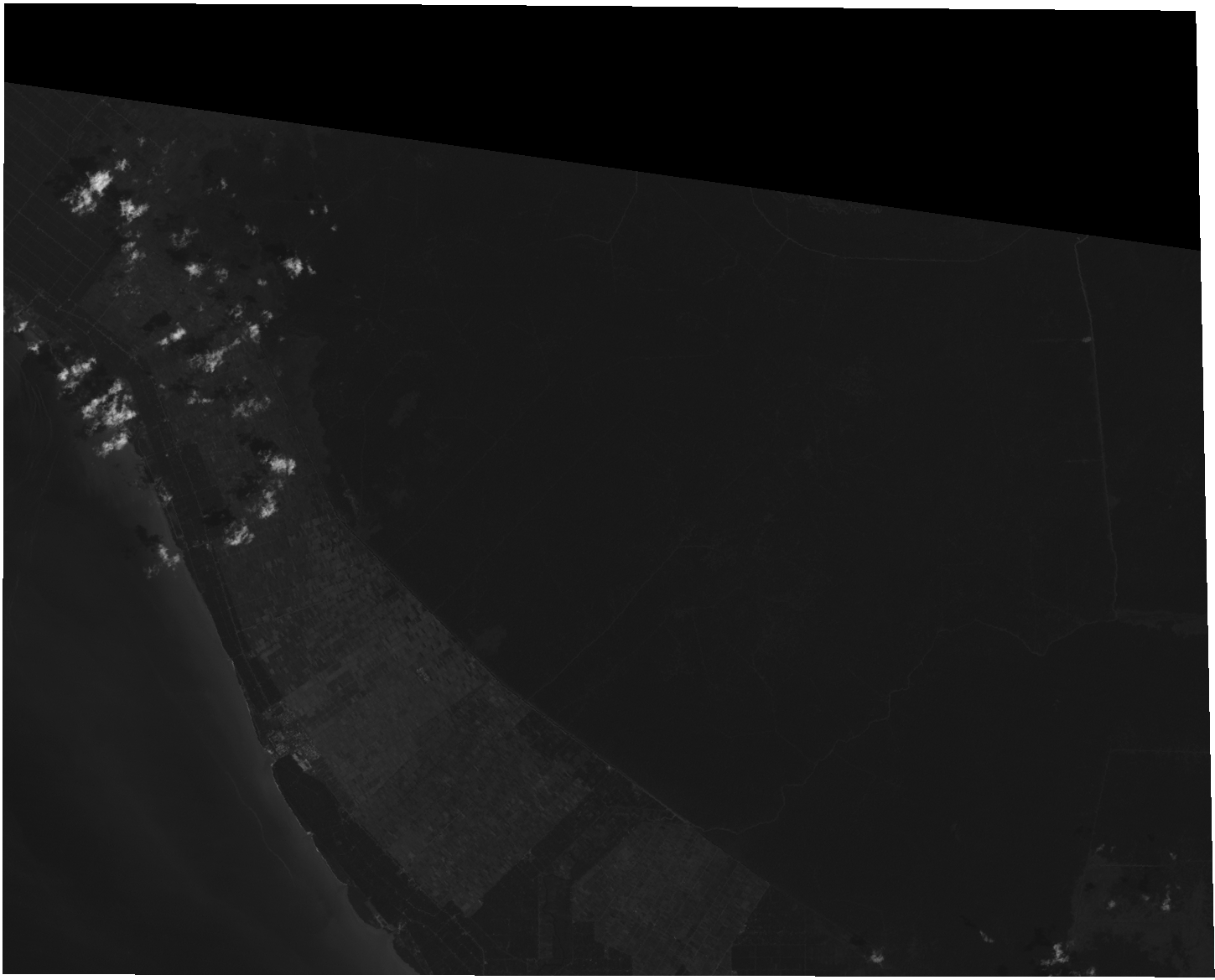

Supplement: S1 File — (ZIP) [file pone.0280187.s001.zip › Landsat1990_clip/p127r58_4t19901227_nn2.tif]

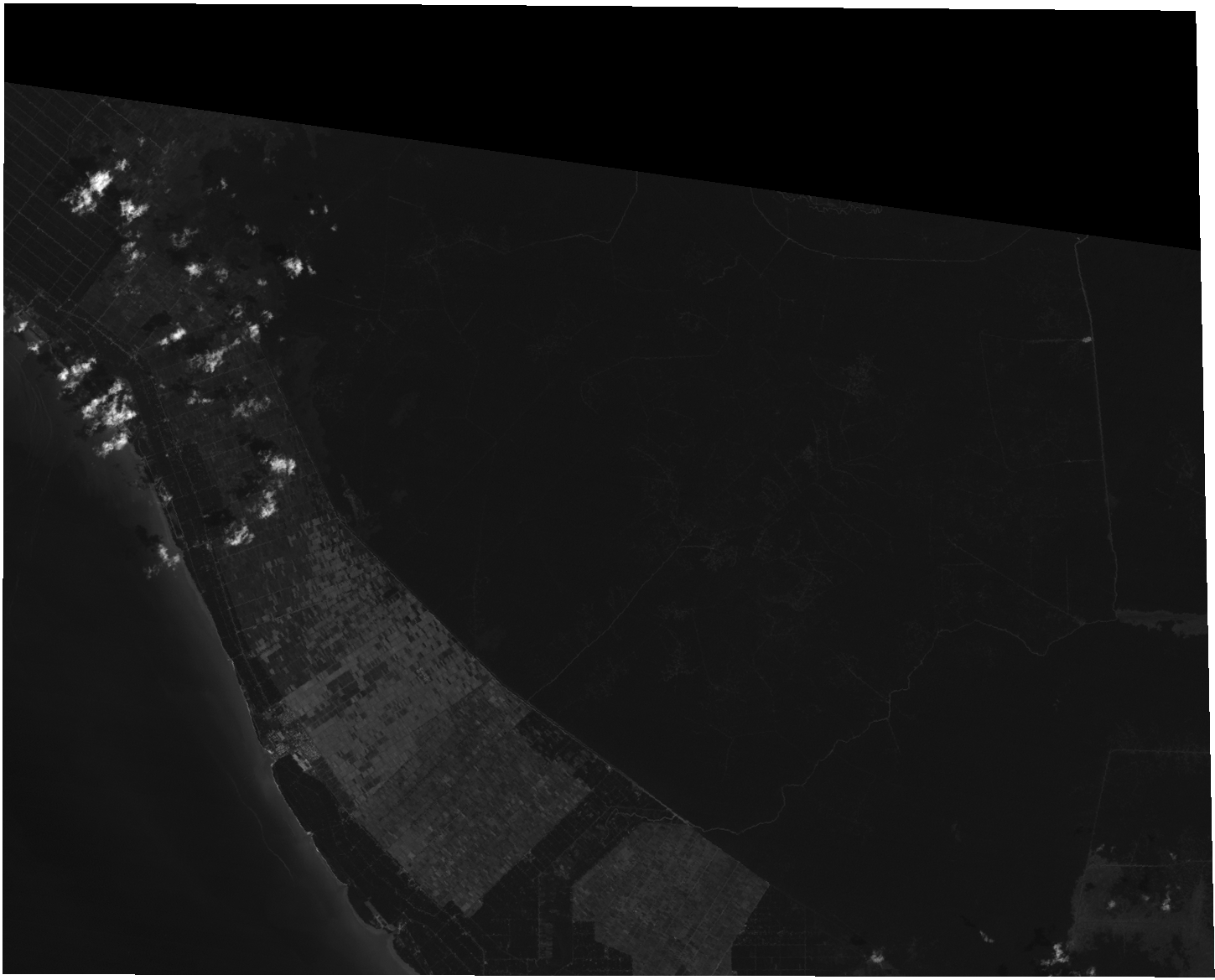

Supplement: S1 File — (ZIP) [file pone.0280187.s001.zip › Landsat1990_clip/p127r58_4t19901227_nn3.tif]

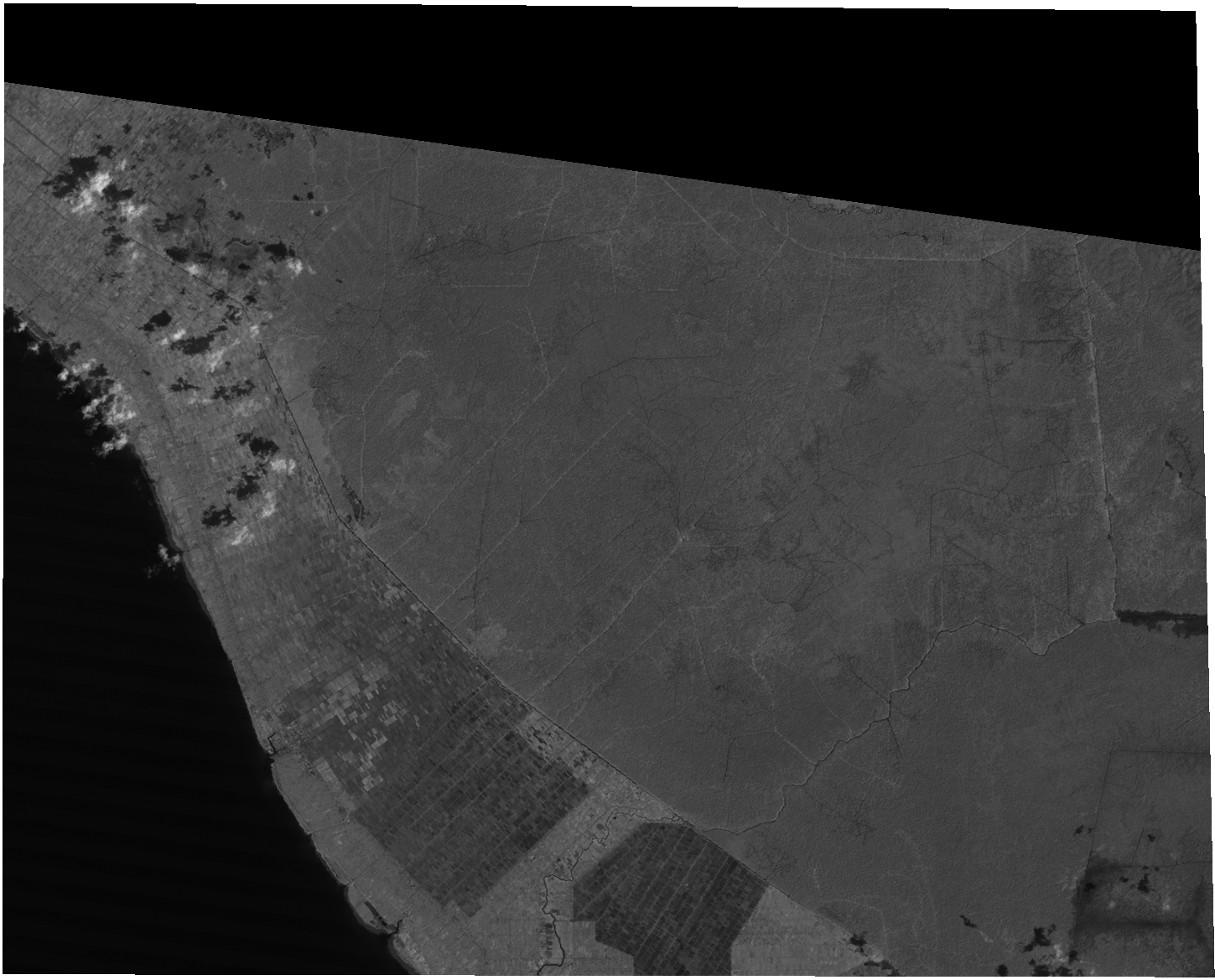

Supplement: S1 File — (ZIP) [file pone.0280187.s001.zip › Landsat1990_clip/p127r58_4t19901227_nn4.tif]

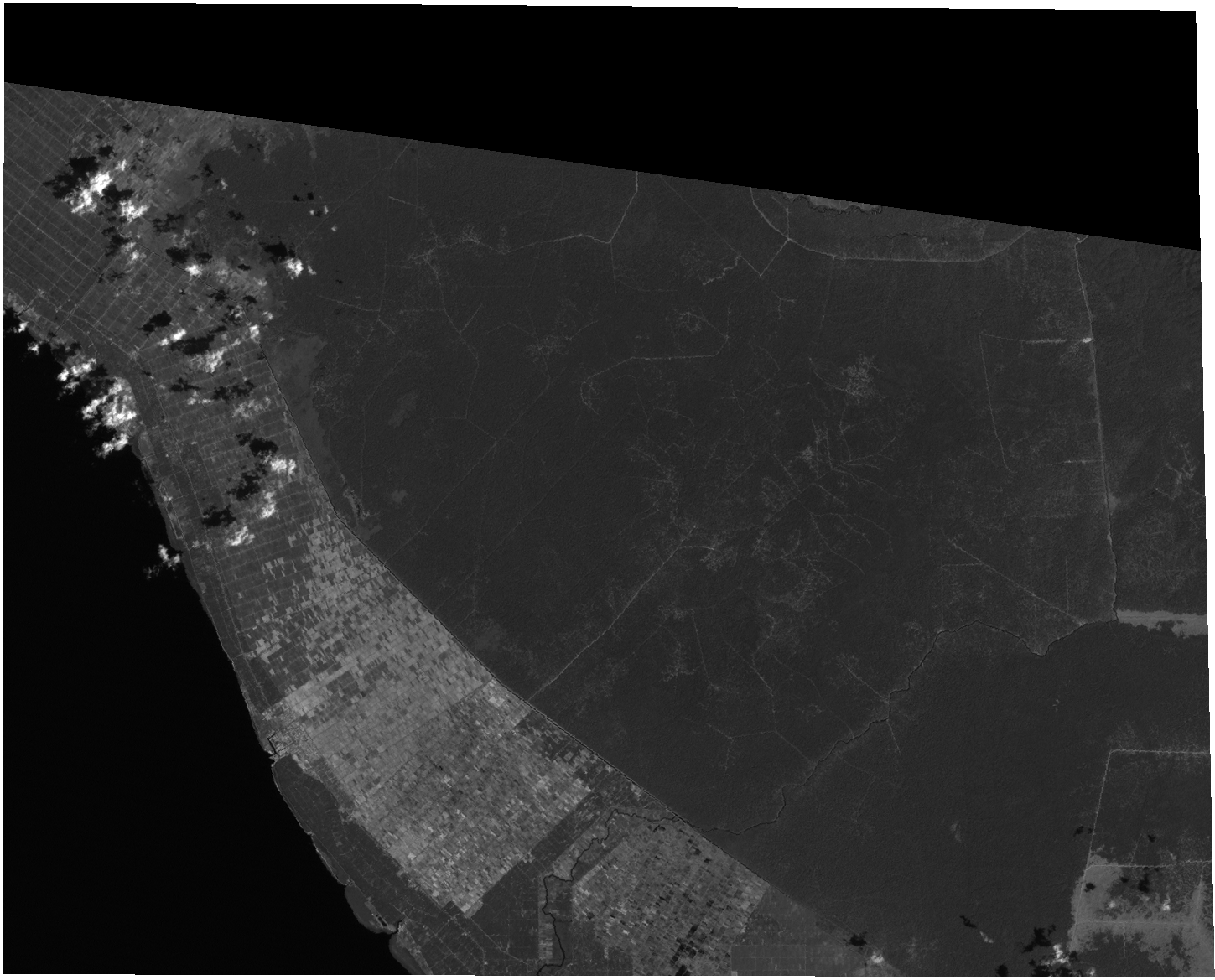

Supplement: S1 File — (ZIP) [file pone.0280187.s001.zip › Landsat1990_clip/p127r58_4t19901227_nn5.tif]

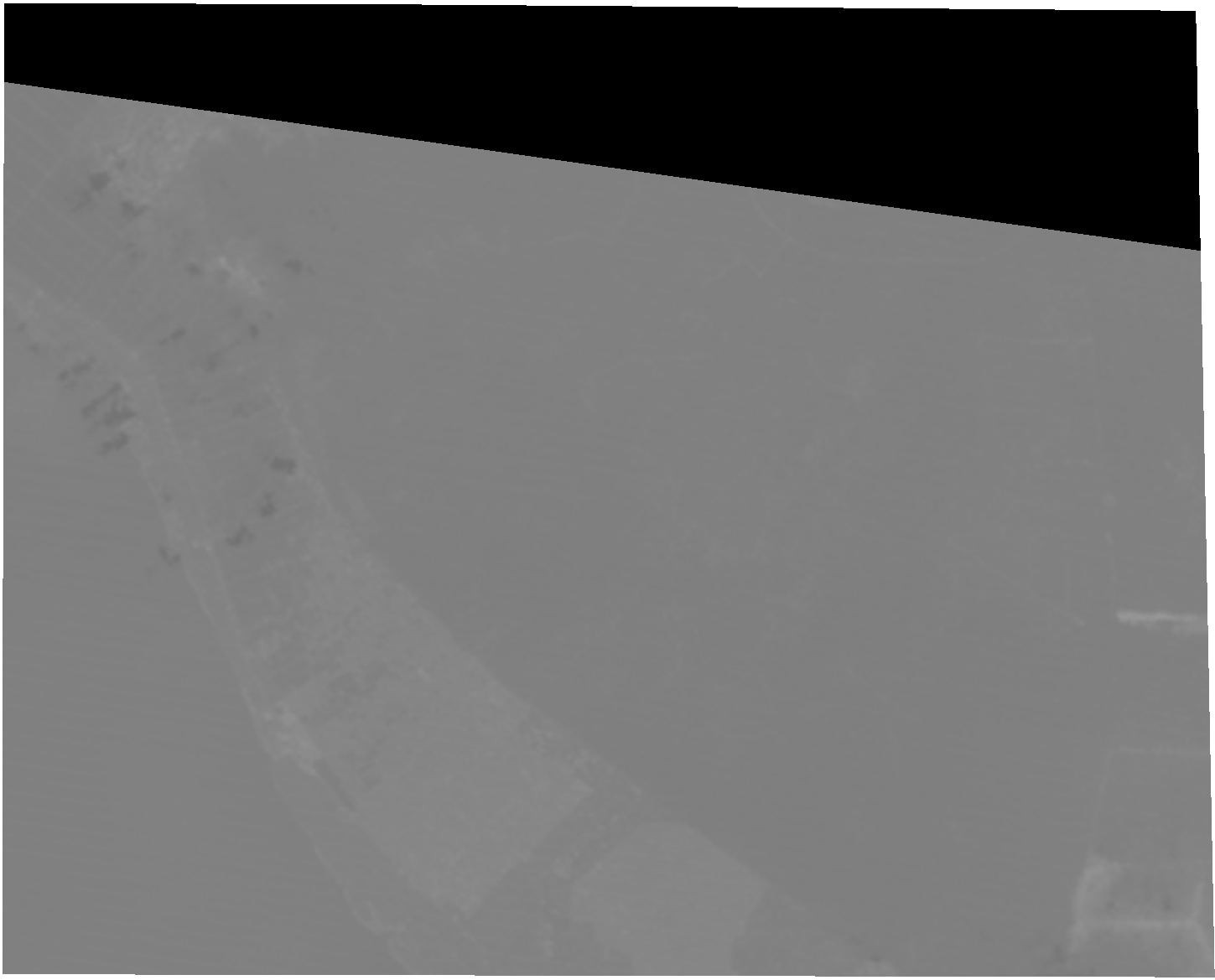

Supplement: S1 File — (ZIP) [file pone.0280187.s001.zip › Landsat1990_clip/p127r58_4t19901227_nn6.tif]

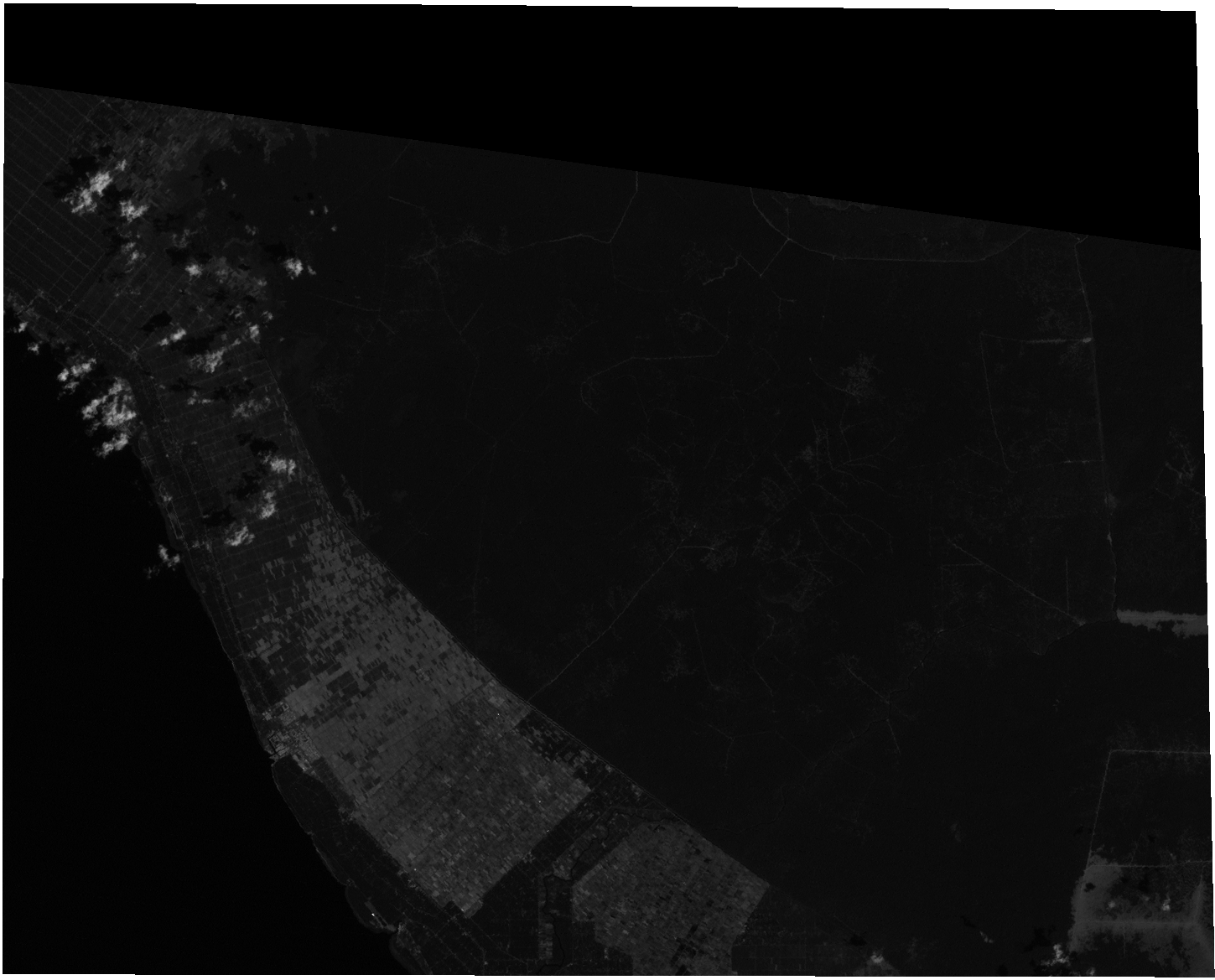

Supplement: S1 File — (ZIP) [file pone.0280187.s001.zip › Landsat1990_clip/p127r58_4t19901227_nn7.tif]

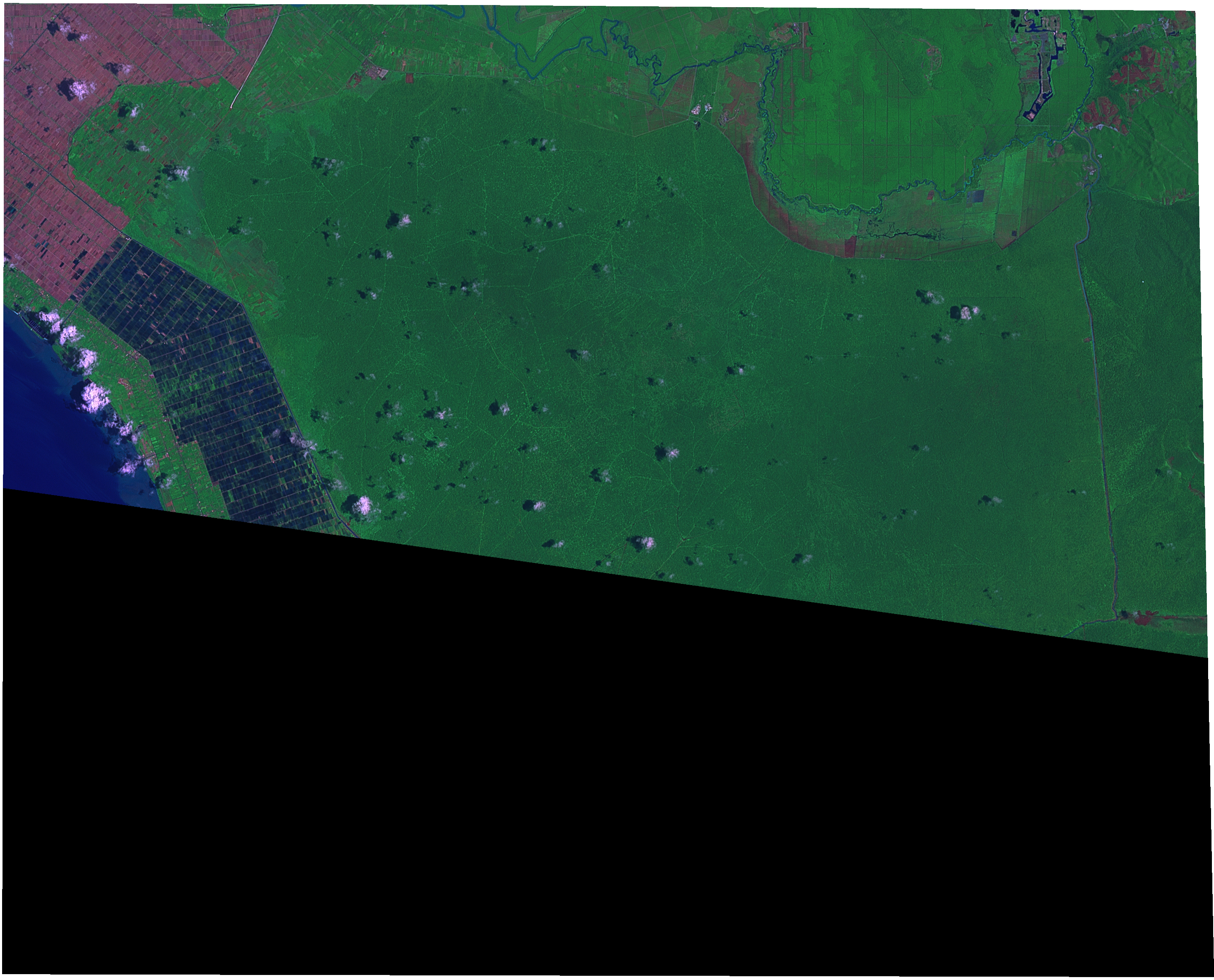

Supplement: S1 File — (ZIP) [file pone.0280187.s001.zip › Landsat2001_clip/p127r057_7f20010920_z47_ps742.tif]

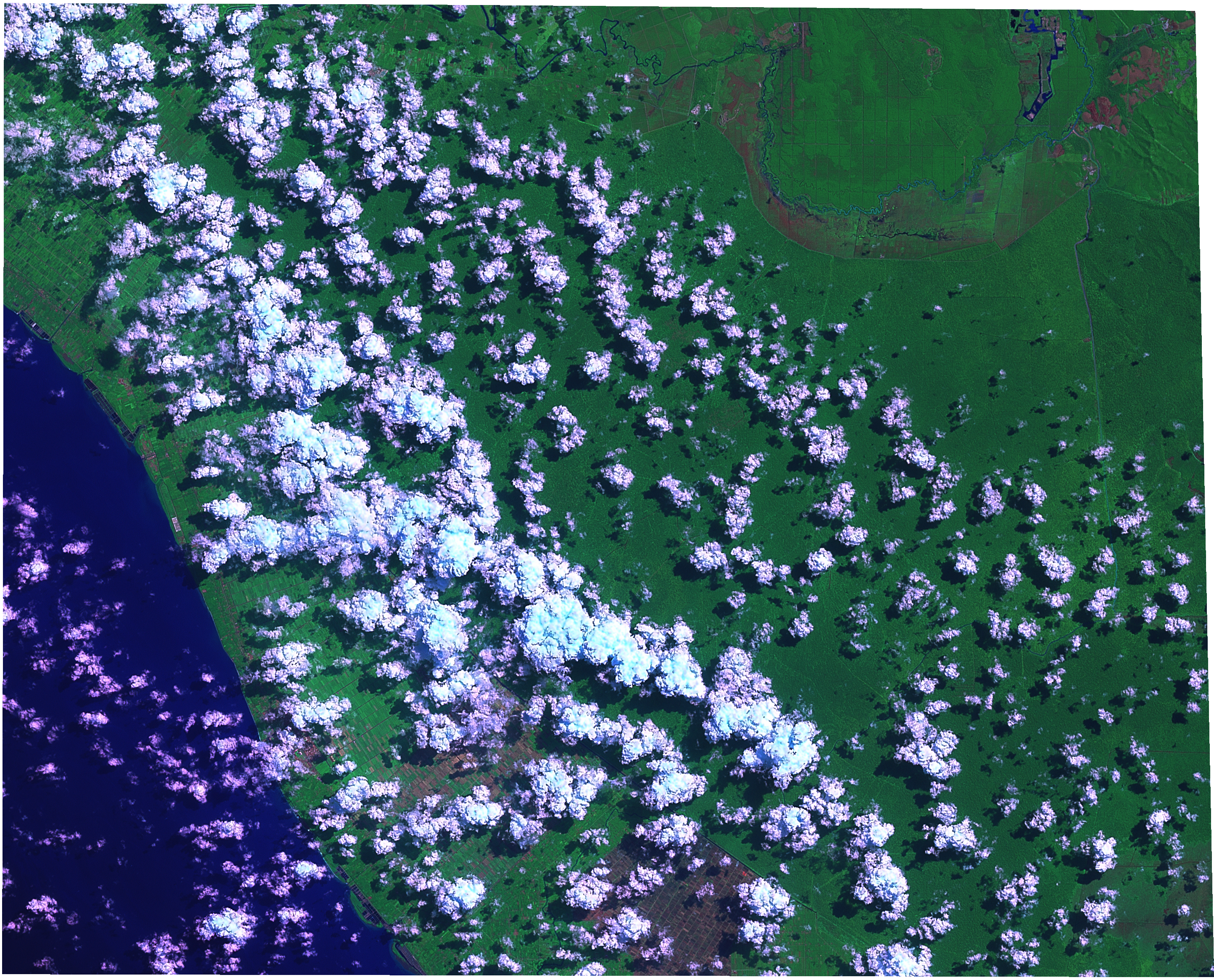

Supplement: S1 File — (ZIP) [file pone.0280187.s001.zip › Landsat2001_clip/p127r058_7f20010531_z47_ps742.tif]

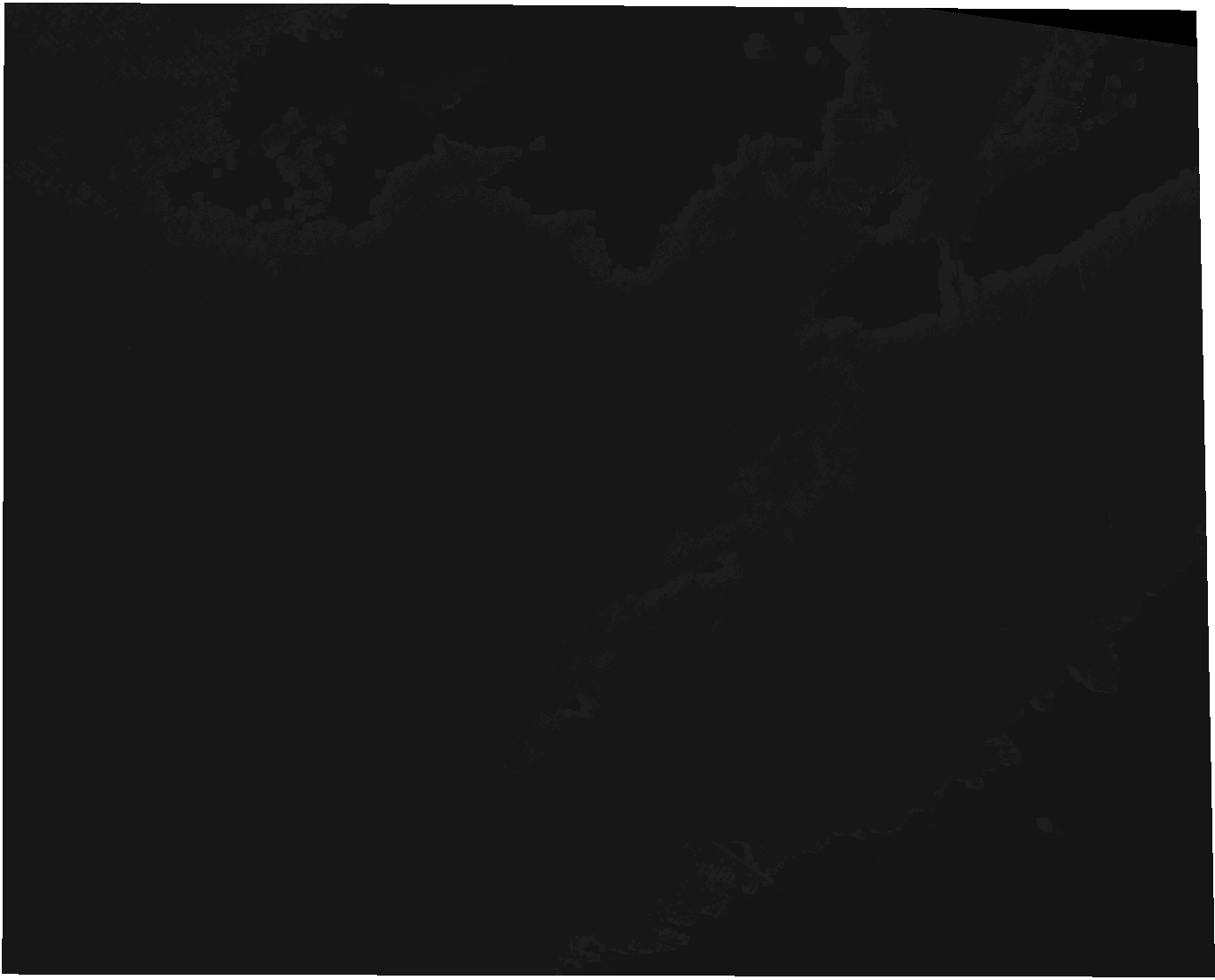

Supplement: S2 File — (ZIP) [file pone.0280187.s002.zip › Landsat2010_clip/LT05_L2SP_127058_20100209_20200824_02_T1_QA_PIXEL.TIF]

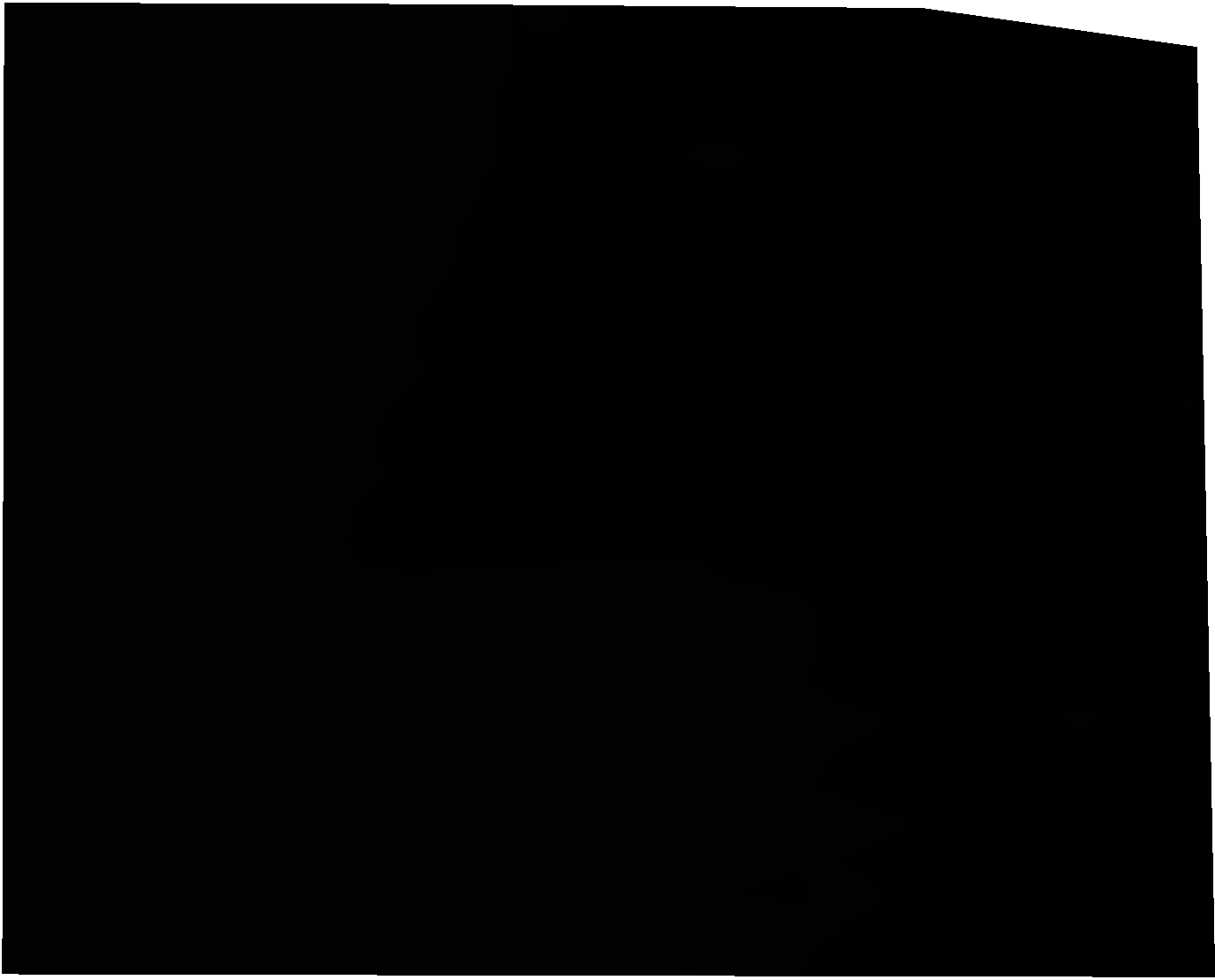

Supplement: S2 File — (ZIP) [file pone.0280187.s002.zip › Landsat2010_clip/LT05_L2SP_127058_20100209_20200824_02_T1_SR_ATMOS_OPACITY.TIF]

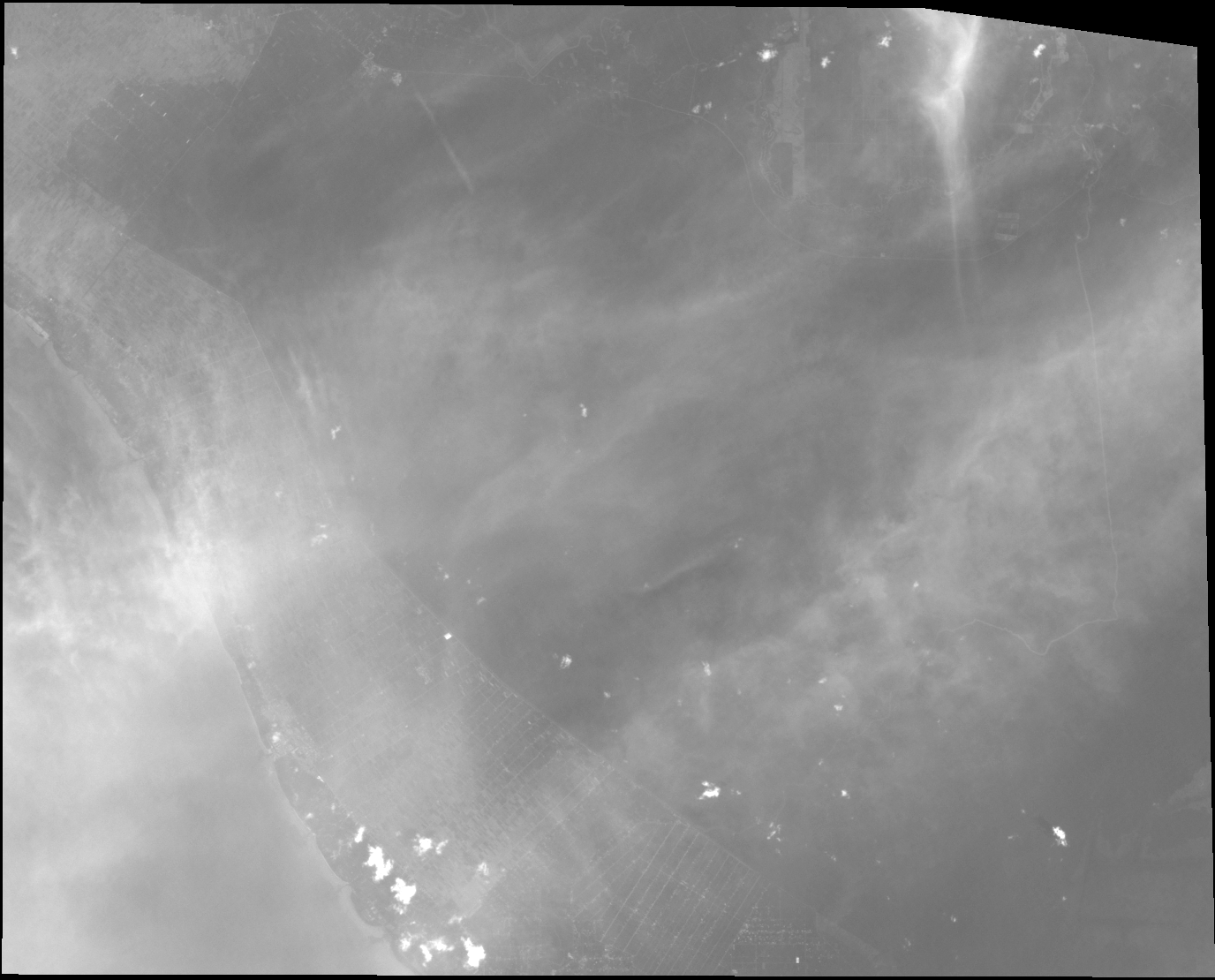

Supplement: S2 File — (ZIP) [file pone.0280187.s002.zip › Landsat2010_clip/LT05_L2SP_127058_20100209_20200824_02_T1_SR_B1.TIF]

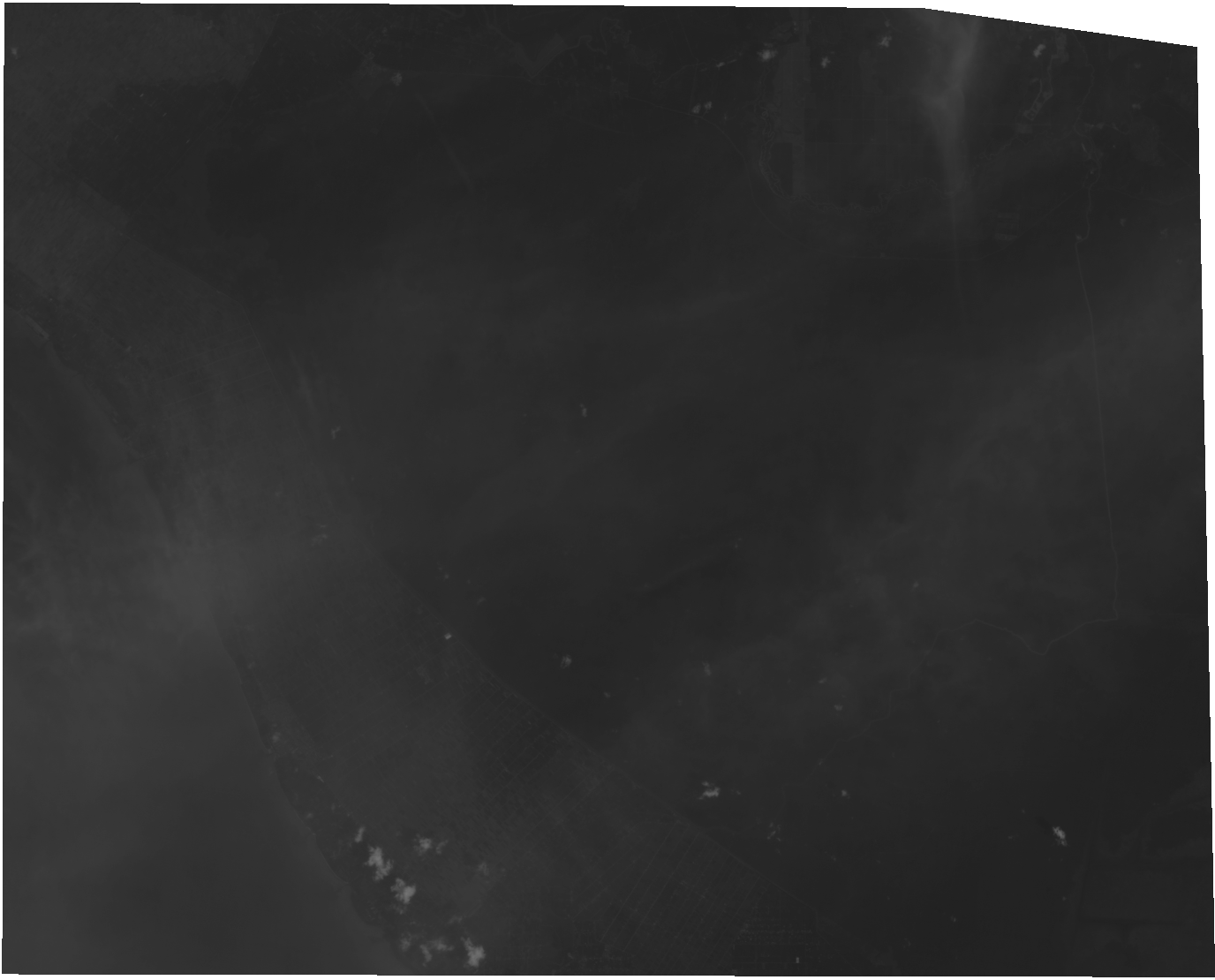

Supplement: S2 File — (ZIP) [file pone.0280187.s002.zip › Landsat2010_clip/LT05_L2SP_127058_20100209_20200824_02_T1_SR_B2.TIF]

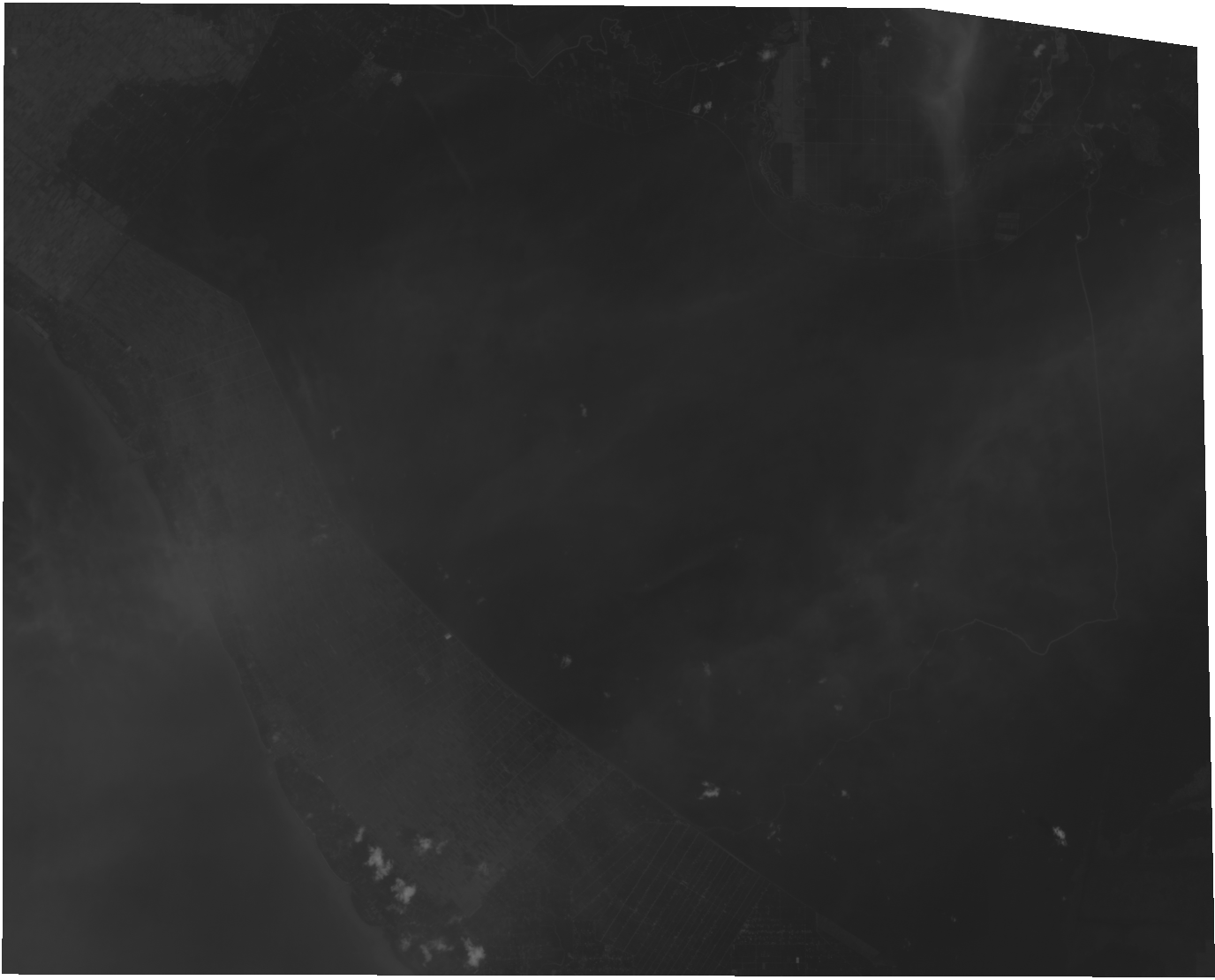

Supplement: S2 File — (ZIP) [file pone.0280187.s002.zip › Landsat2010_clip/LT05_L2SP_127058_20100209_20200824_02_T1_SR_B3.TIF]

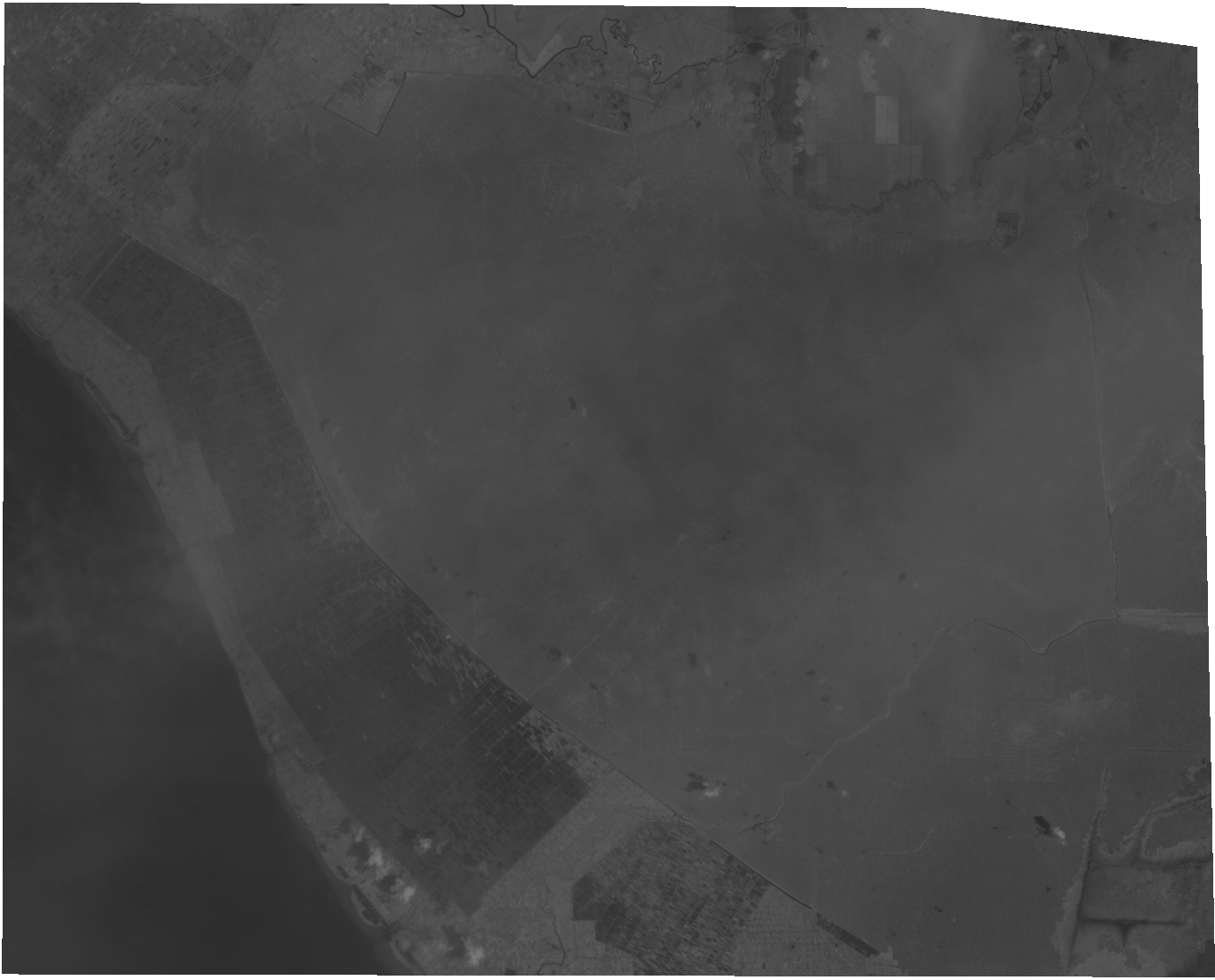

Supplement: S2 File — (ZIP) [file pone.0280187.s002.zip › Landsat2010_clip/LT05_L2SP_127058_20100209_20200824_02_T1_SR_B4.TIF]

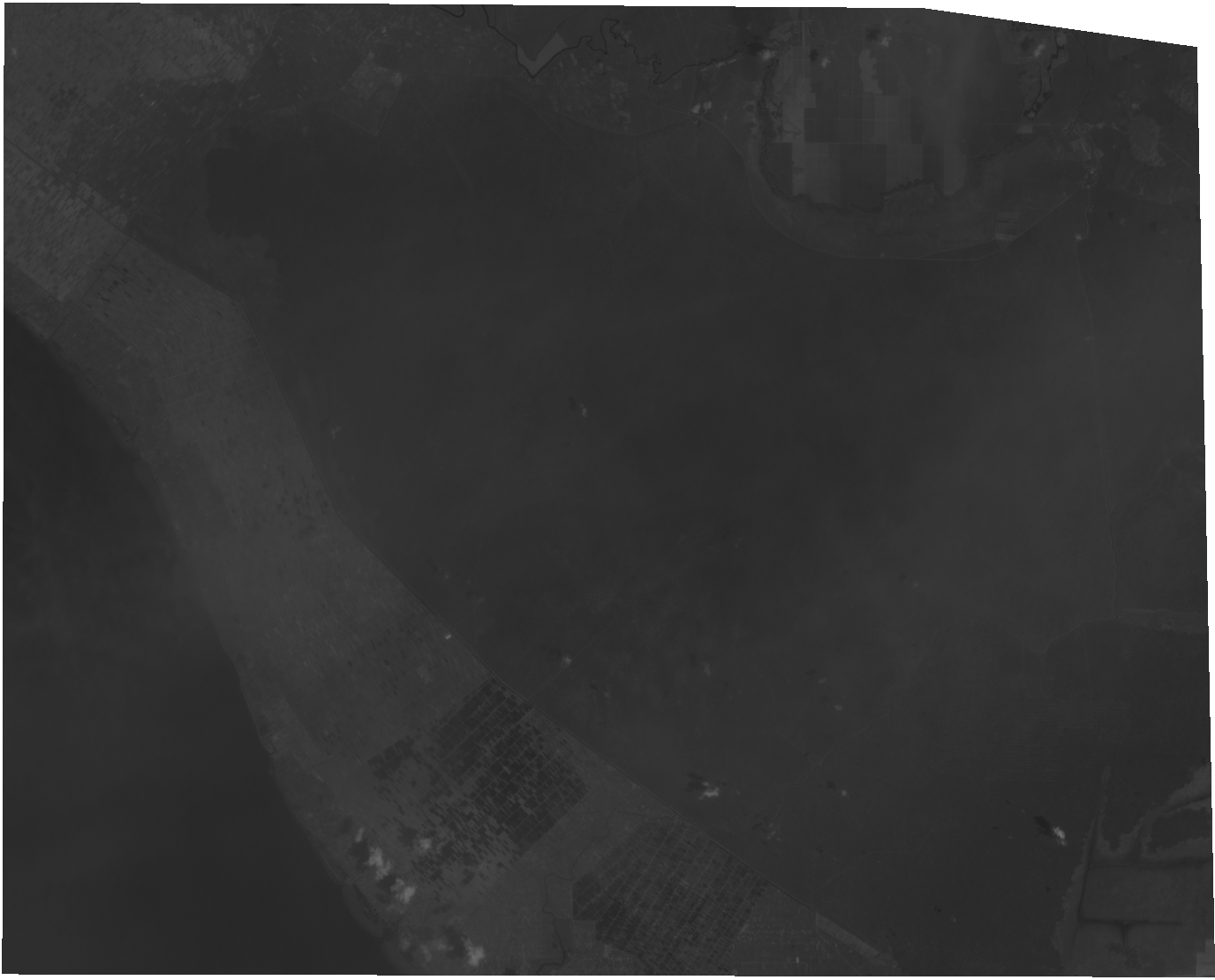

Supplement: S2 File — (ZIP) [file pone.0280187.s002.zip › Landsat2010_clip/LT05_L2SP_127058_20100209_20200824_02_T1_SR_B5.TIF]

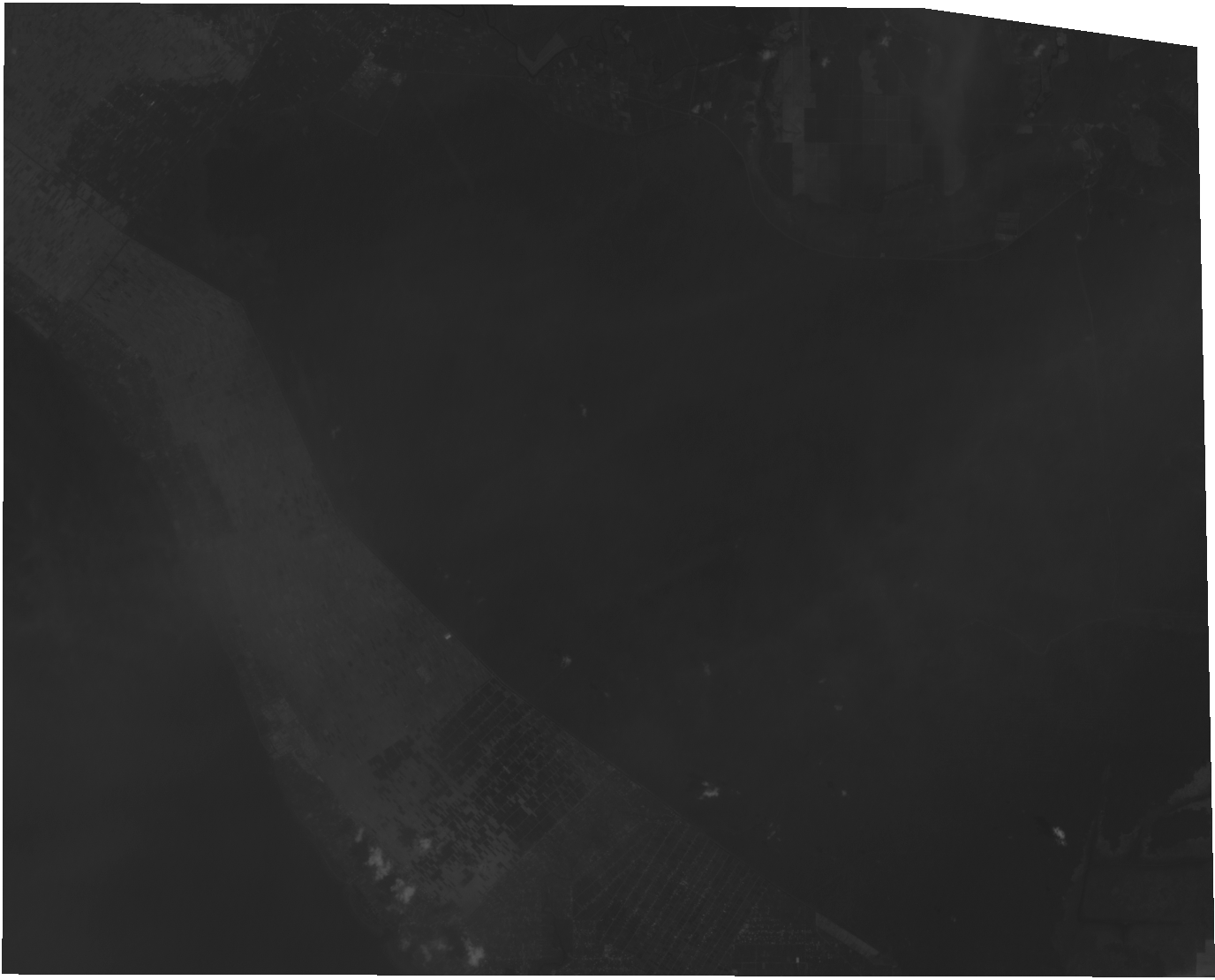

Supplement: S2 File — (ZIP) [file pone.0280187.s002.zip › Landsat2010_clip/LT05_L2SP_127058_20100209_20200824_02_T1_SR_B7.TIF]

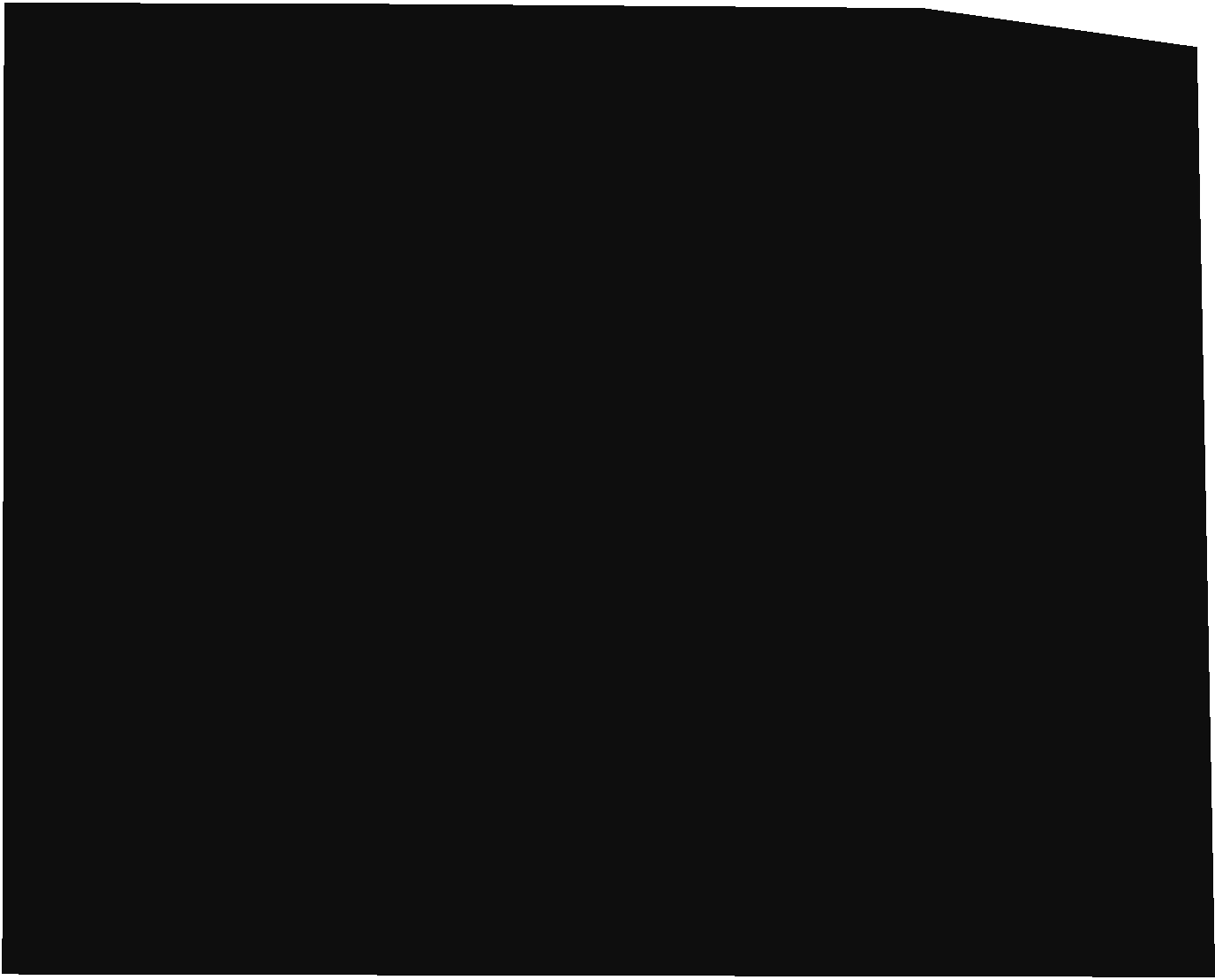

Supplement: S2 File — (ZIP) [file pone.0280187.s002.zip › Landsat2010_clip/LT05_L2SP_127058_20100209_20200824_02_T1_ST_ATRAN.TIF]

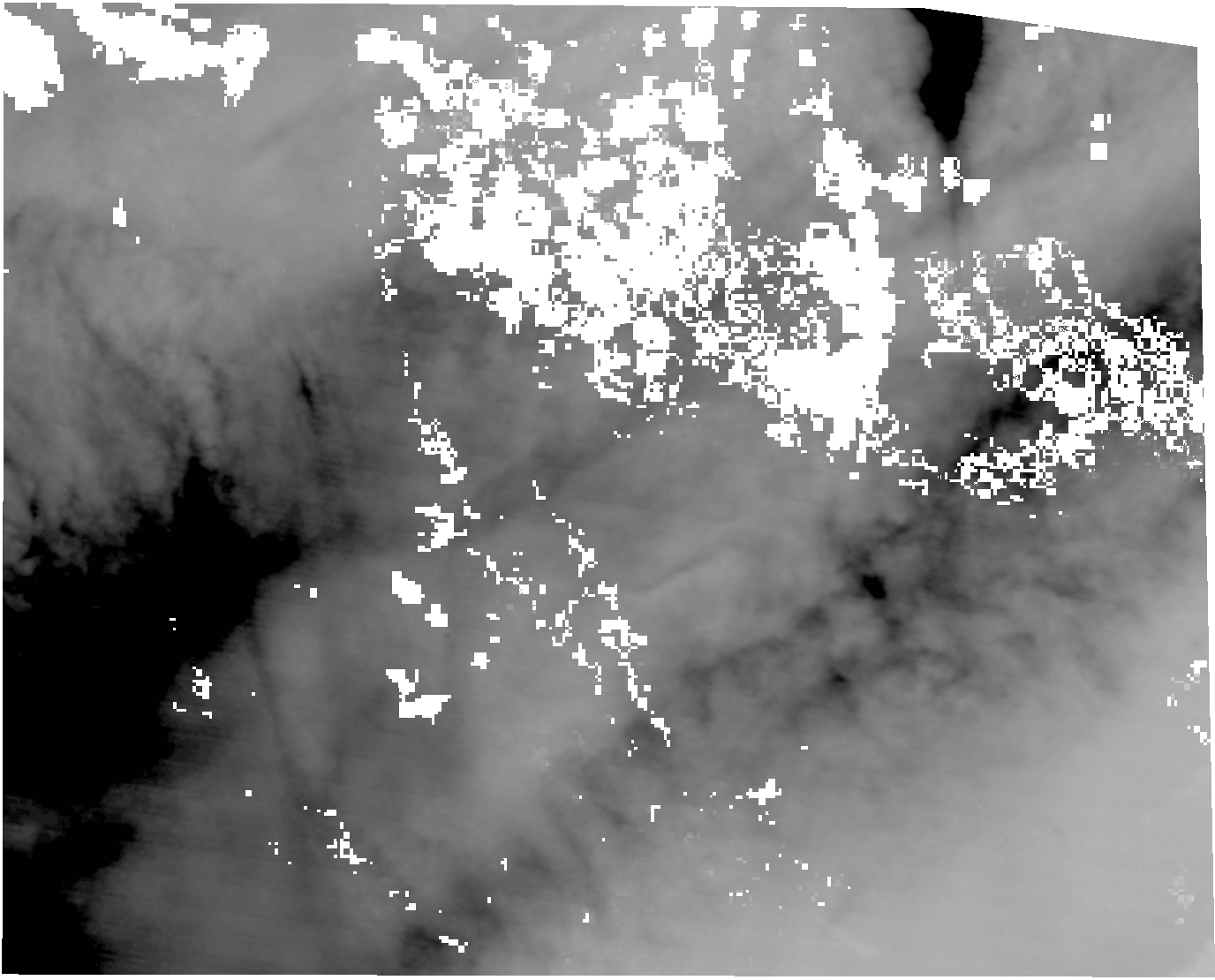

Supplement: S2 File — (ZIP) [file pone.0280187.s002.zip › Landsat2010_clip/LT05_L2SP_127058_20100209_20200824_02_T1_ST_B6.TIF]

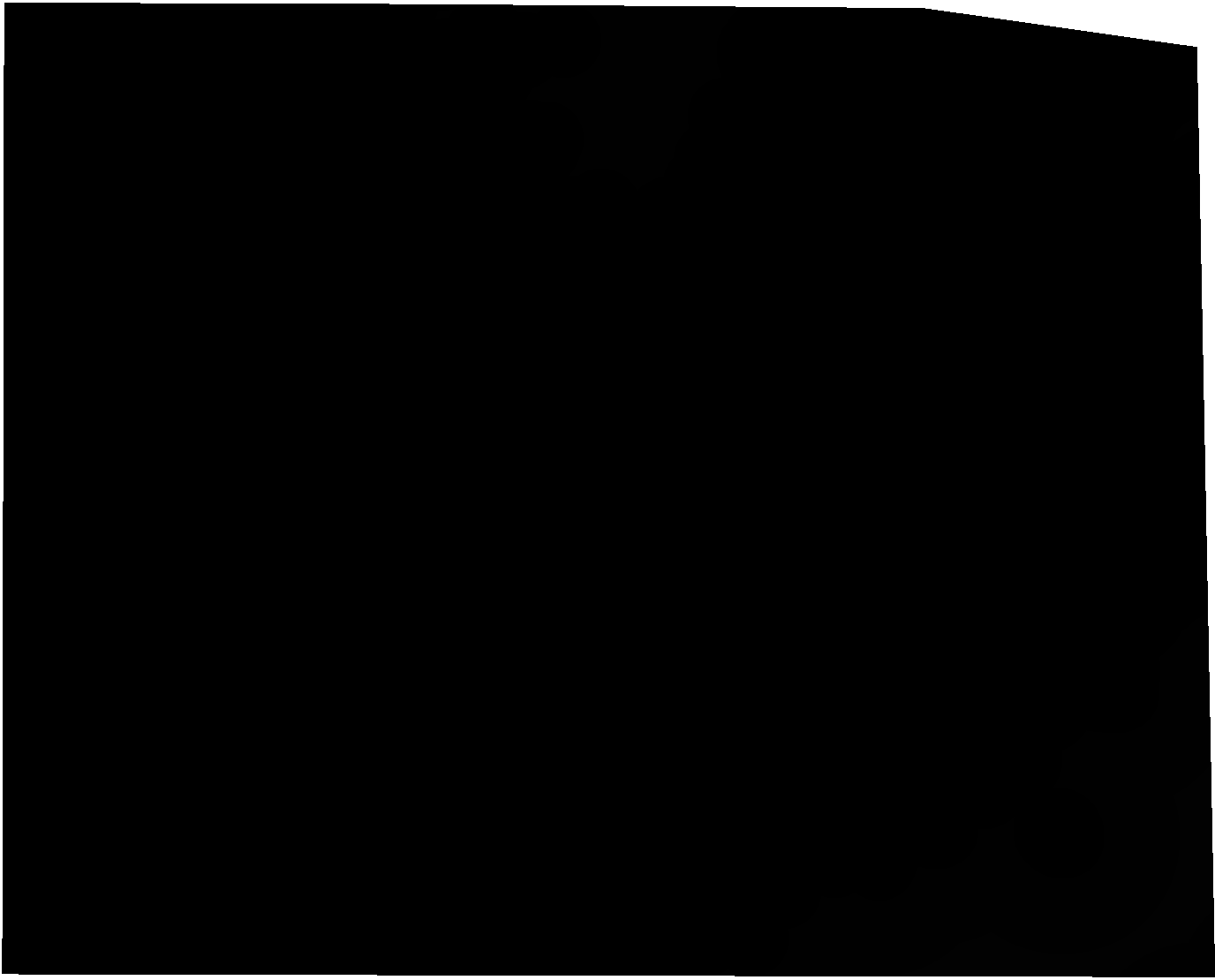

Supplement: S2 File — (ZIP) [file pone.0280187.s002.zip › Landsat2010_clip/LT05_L2SP_127058_20100209_20200824_02_T1_ST_CDIST.TIF]

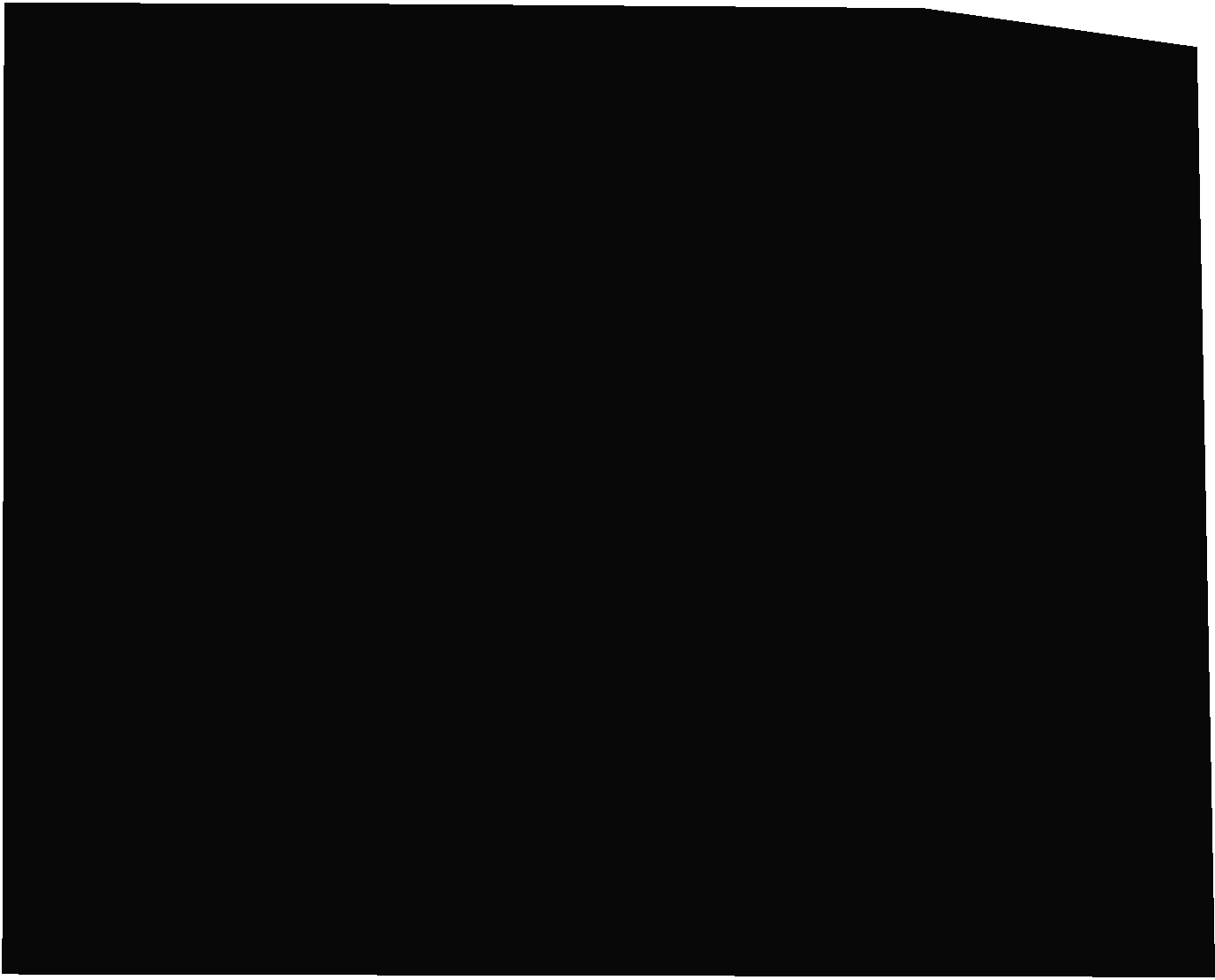

Supplement: S2 File — (ZIP) [file pone.0280187.s002.zip › Landsat2010_clip/LT05_L2SP_127058_20100209_20200824_02_T1_ST_DRAD.TIF]

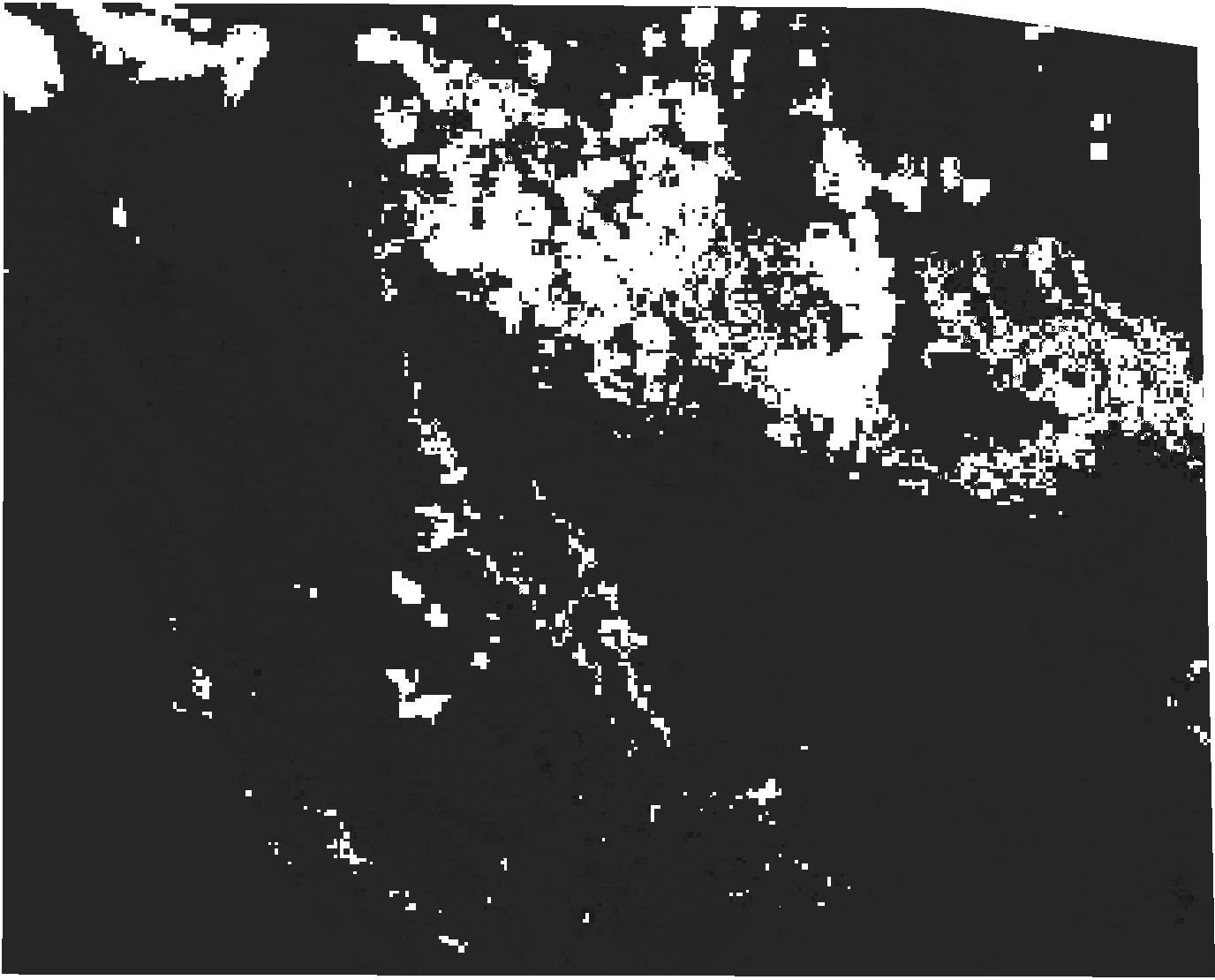

Supplement: S2 File — (ZIP) [file pone.0280187.s002.zip › Landsat2010_clip/LT05_L2SP_127058_20100209_20200824_02_T1_ST_EMIS.TIF]

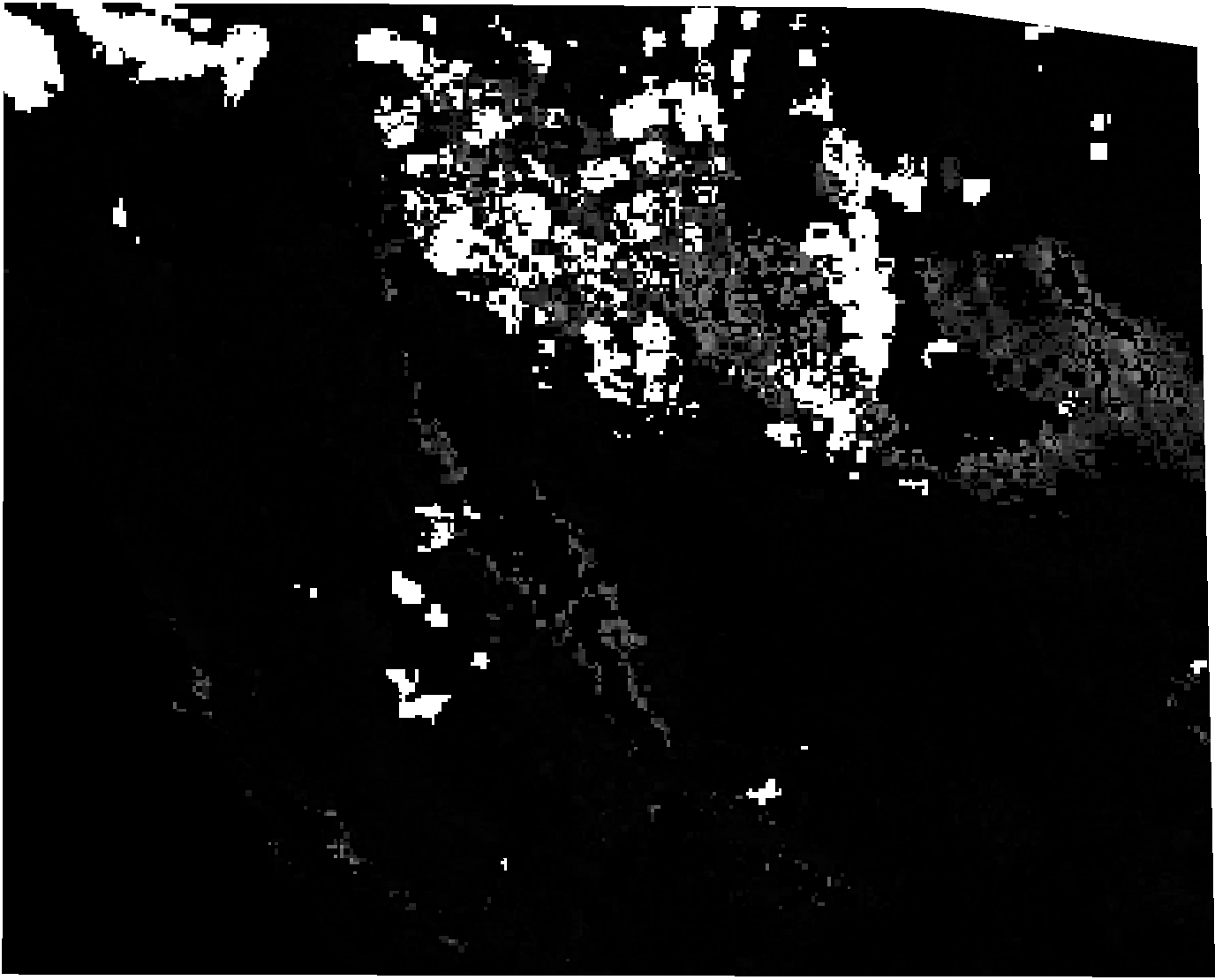

Supplement: S2 File — (ZIP) [file pone.0280187.s002.zip › Landsat2010_clip/LT05_L2SP_127058_20100209_20200824_02_T1_ST_EMSD.TIF]

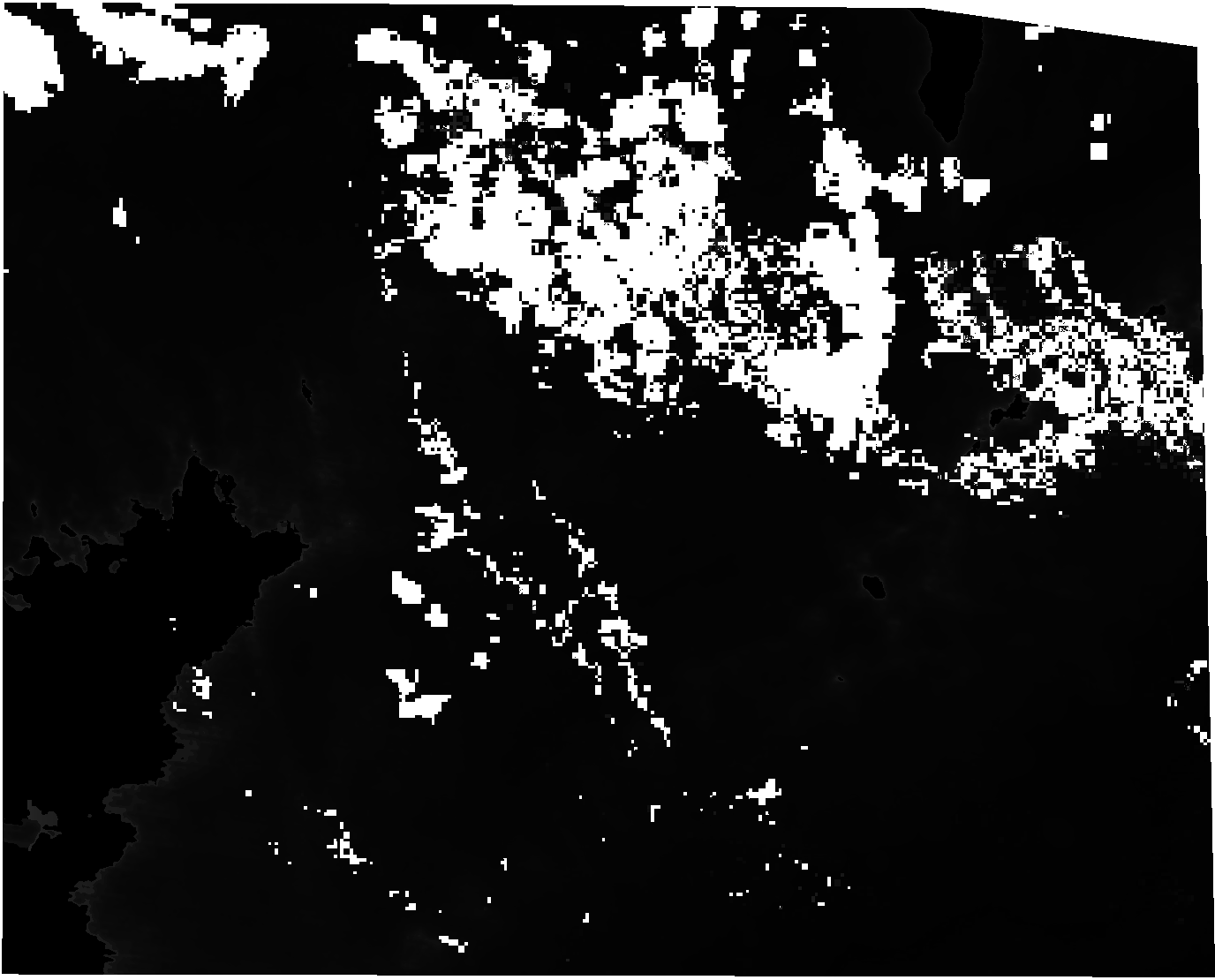

Supplement: S2 File — (ZIP) [file pone.0280187.s002.zip › Landsat2010_clip/LT05_L2SP_127058_20100209_20200824_02_T1_ST_QA.TIF]

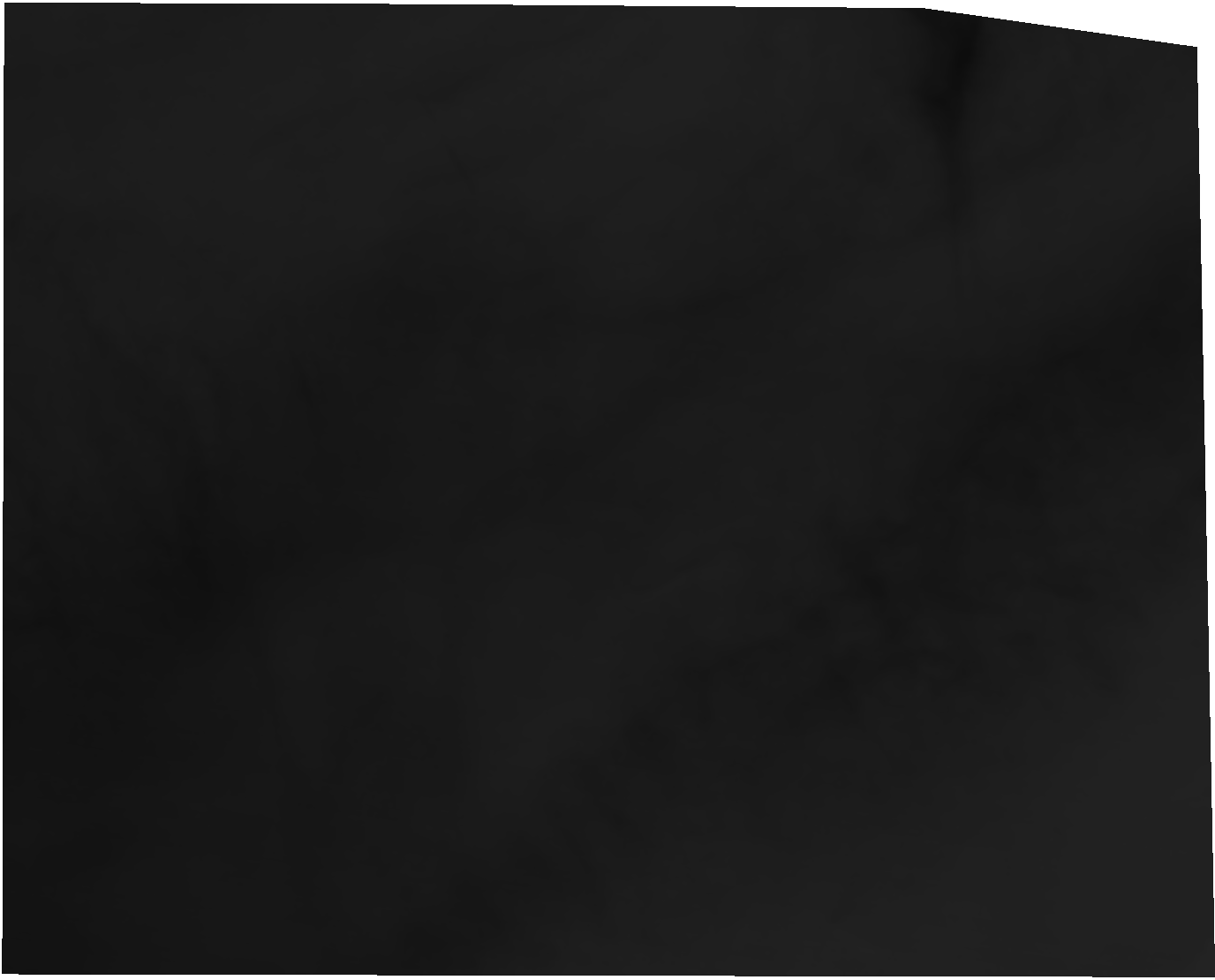

Supplement: S2 File — (ZIP) [file pone.0280187.s002.zip › Landsat2010_clip/LT05_L2SP_127058_20100209_20200824_02_T1_ST_TRAD.TIF]

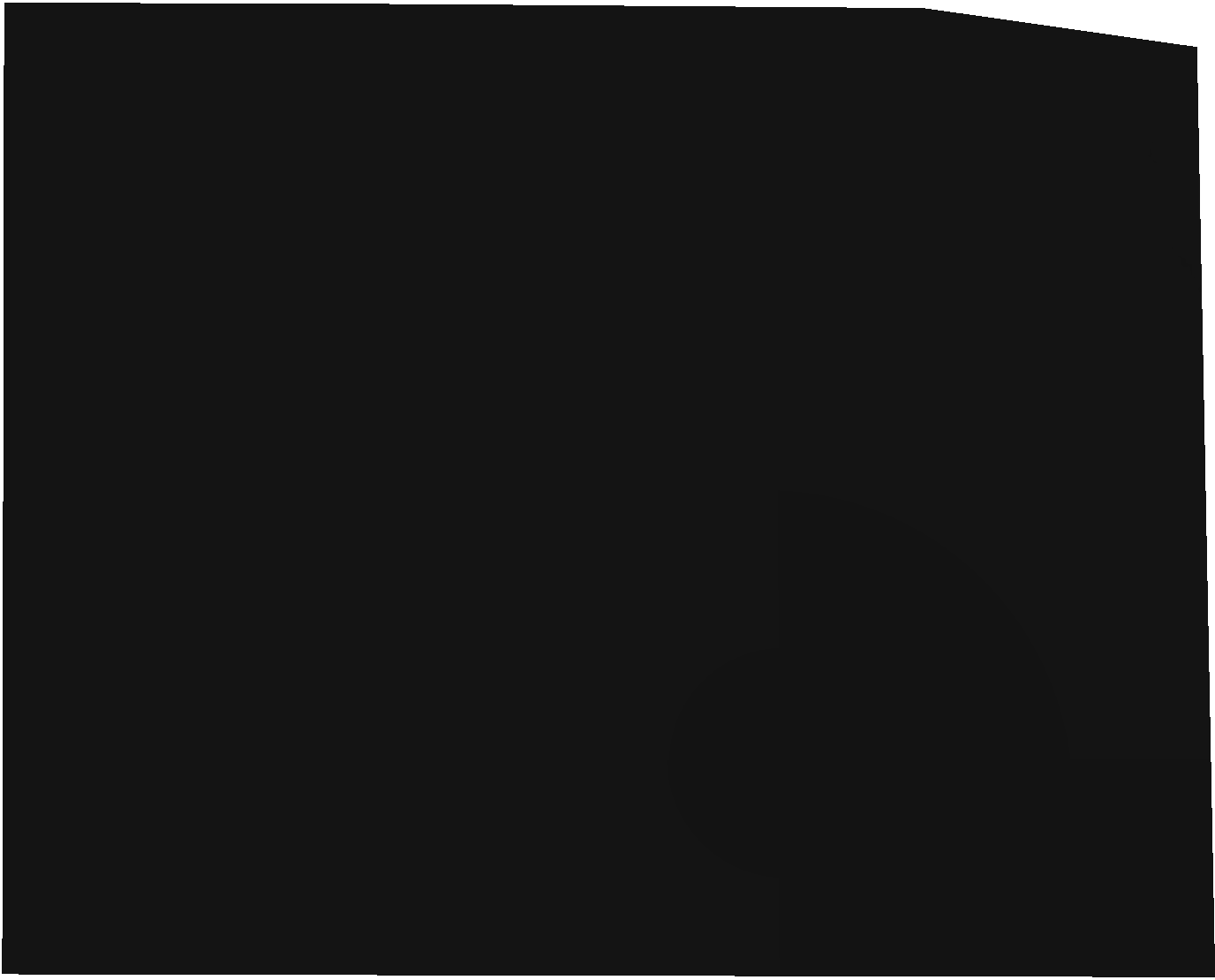

Supplement: S2 File — (ZIP) [file pone.0280187.s002.zip › Landsat2010_clip/LT05_L2SP_127058_20100209_20200824_02_T1_ST_URAD.TIF]

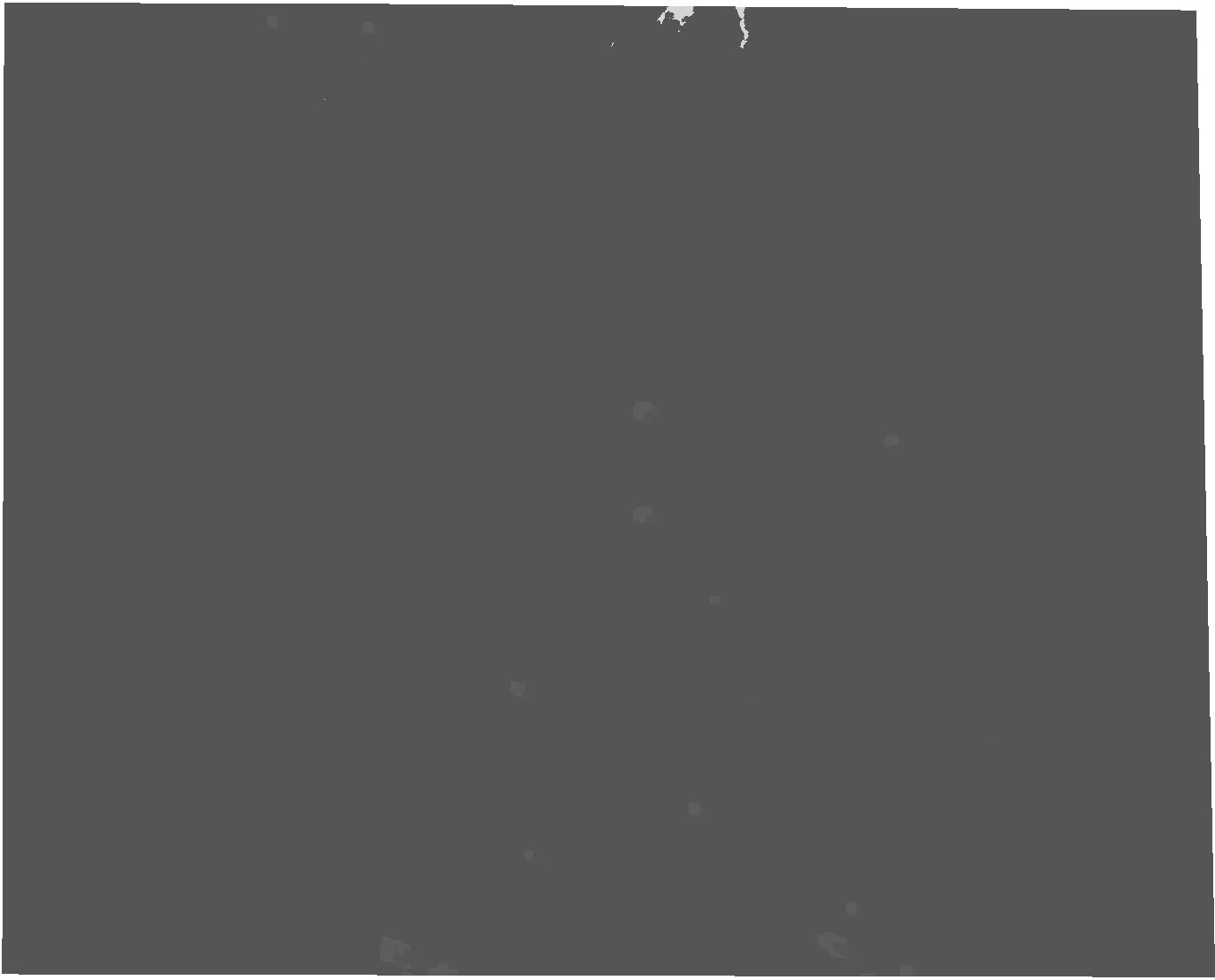

Supplement: S2 File — (ZIP) [file pone.0280187.s002.zip › Landsat2019_clip/LC08_L2SP_127058_20191016_20200825_02_T1_QA_PIXEL.TIF]

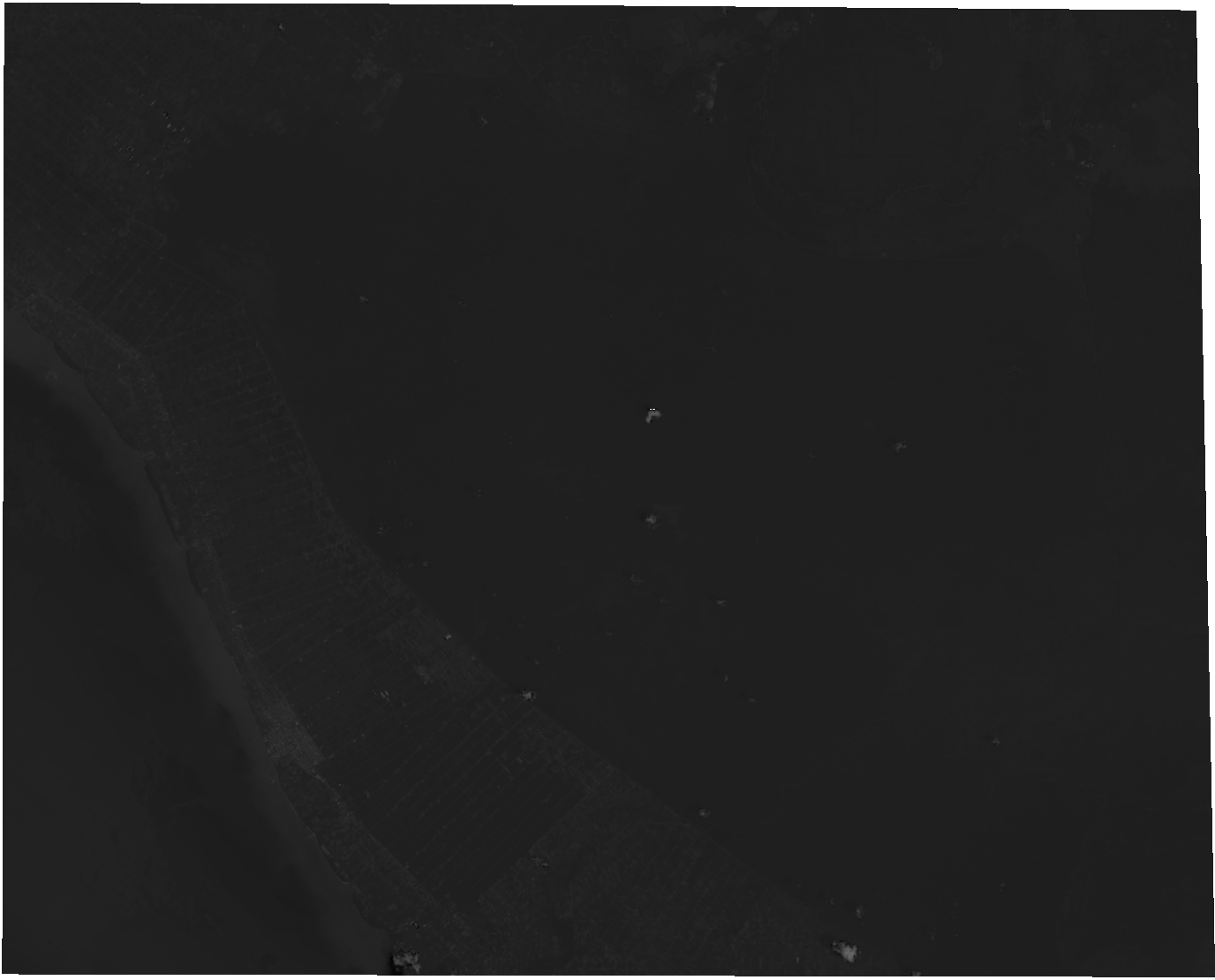

Supplement: S2 File — (ZIP) [file pone.0280187.s002.zip › Landsat2019_clip/LC08_L2SP_127058_20191016_20200825_02_T1_SR_B1.TIF]

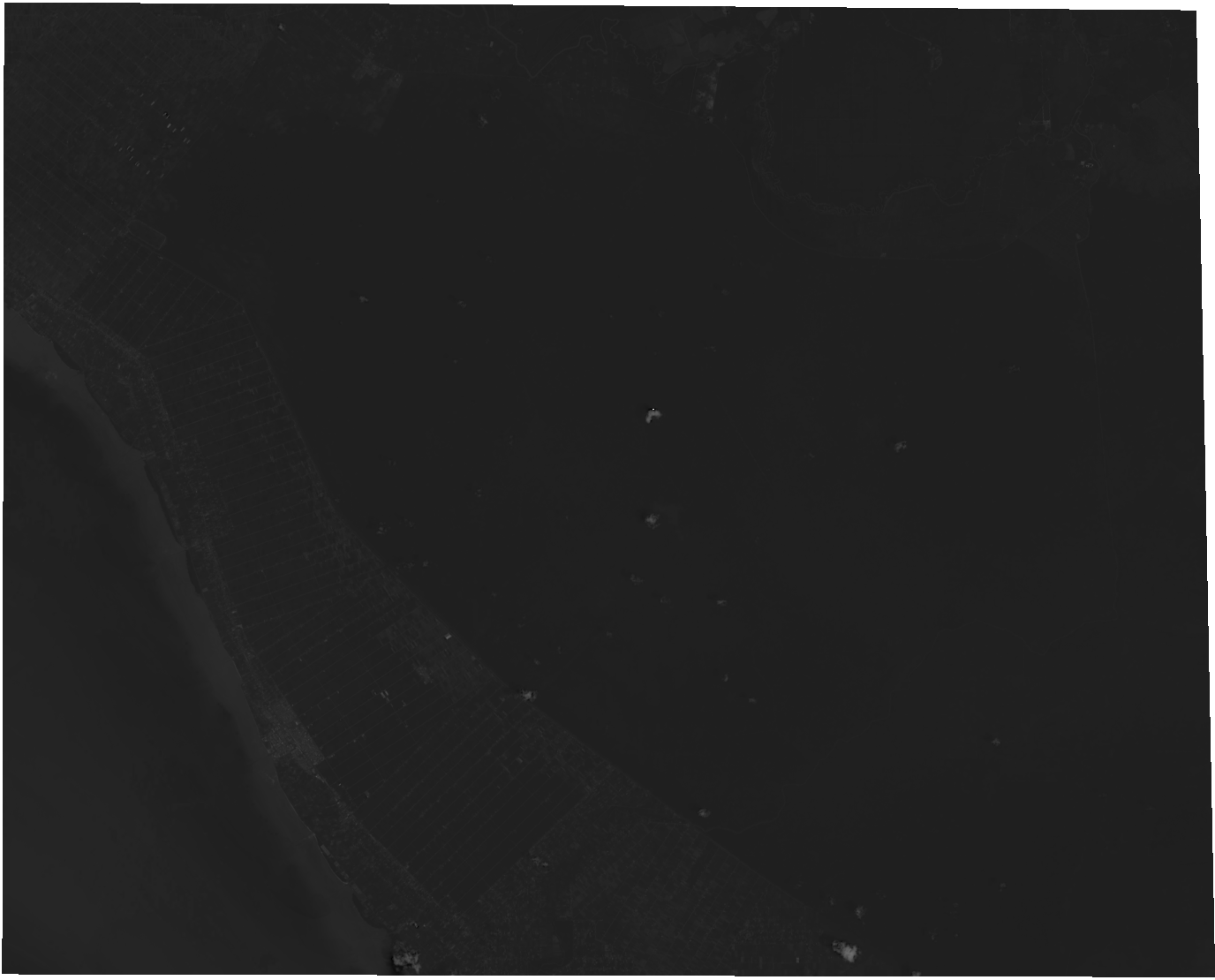

Supplement: S2 File — (ZIP) [file pone.0280187.s002.zip › Landsat2019_clip/LC08_L2SP_127058_20191016_20200825_02_T1_SR_B2.TIF]

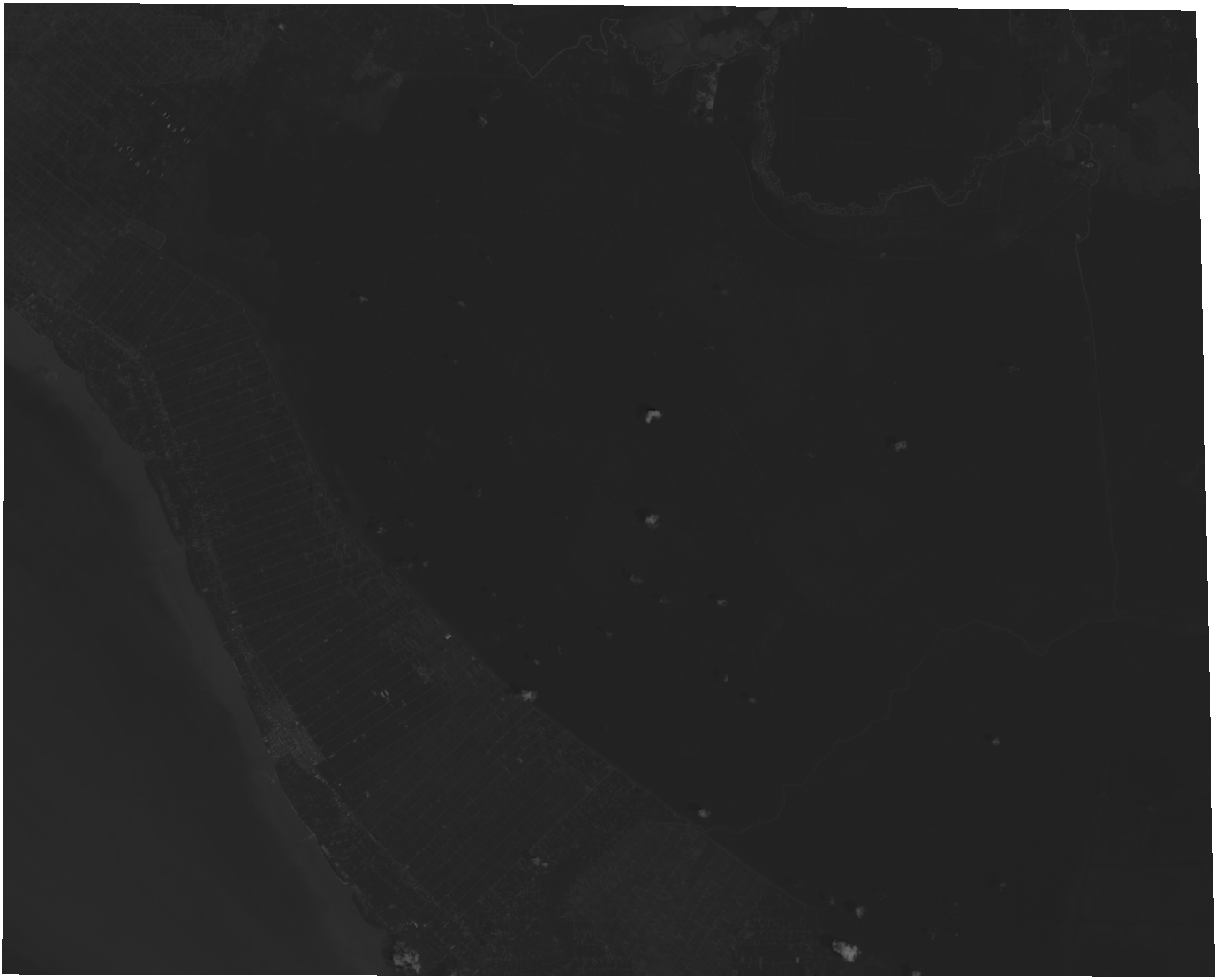

Supplement: S2 File — (ZIP) [file pone.0280187.s002.zip › Landsat2019_clip/LC08_L2SP_127058_20191016_20200825_02_T1_SR_B3.TIF]

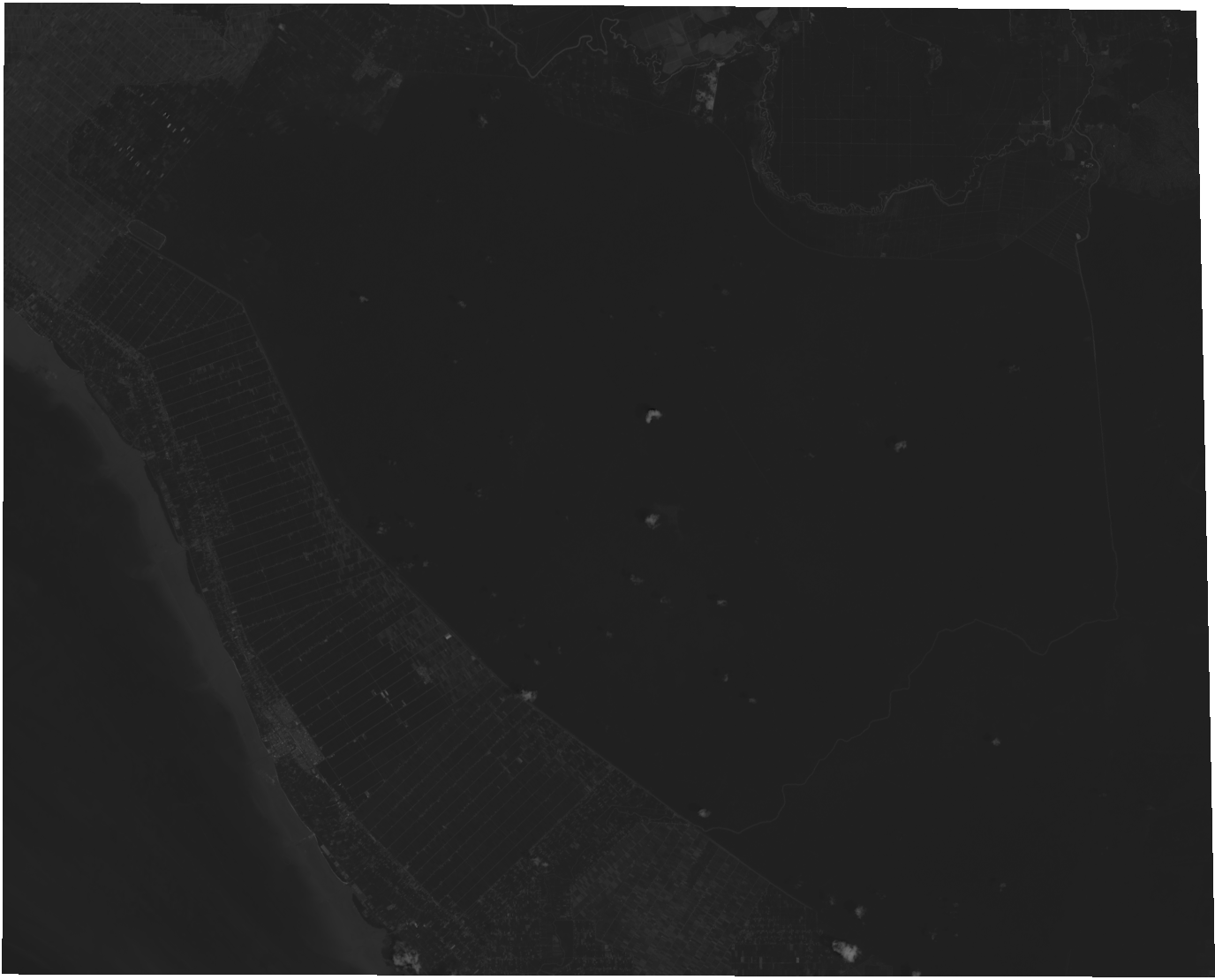

Supplement: S2 File — (ZIP) [file pone.0280187.s002.zip › Landsat2019_clip/LC08_L2SP_127058_20191016_20200825_02_T1_SR_B4.TIF]

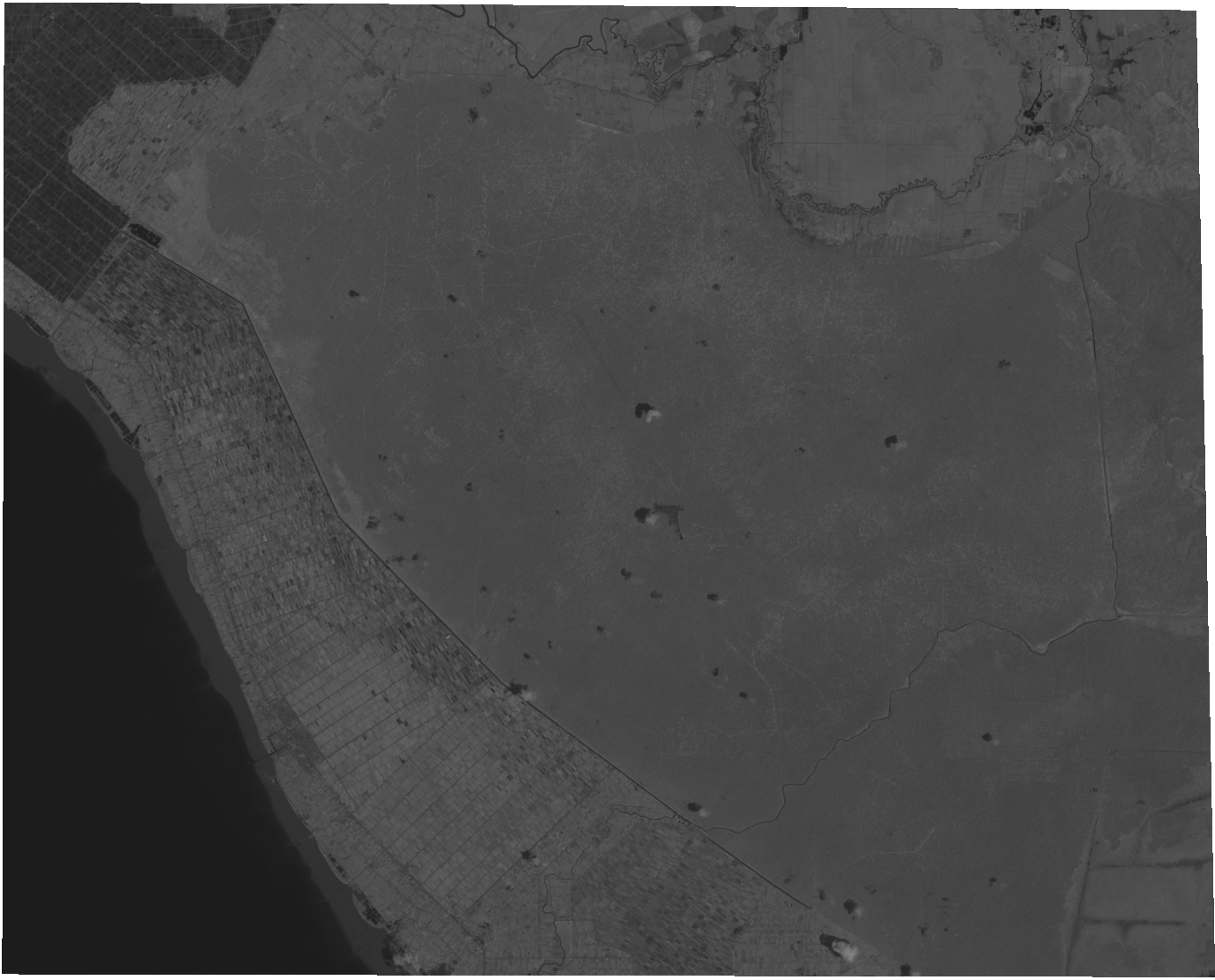

Supplement: S2 File — (ZIP) [file pone.0280187.s002.zip › Landsat2019_clip/LC08_L2SP_127058_20191016_20200825_02_T1_SR_B5.TIF]

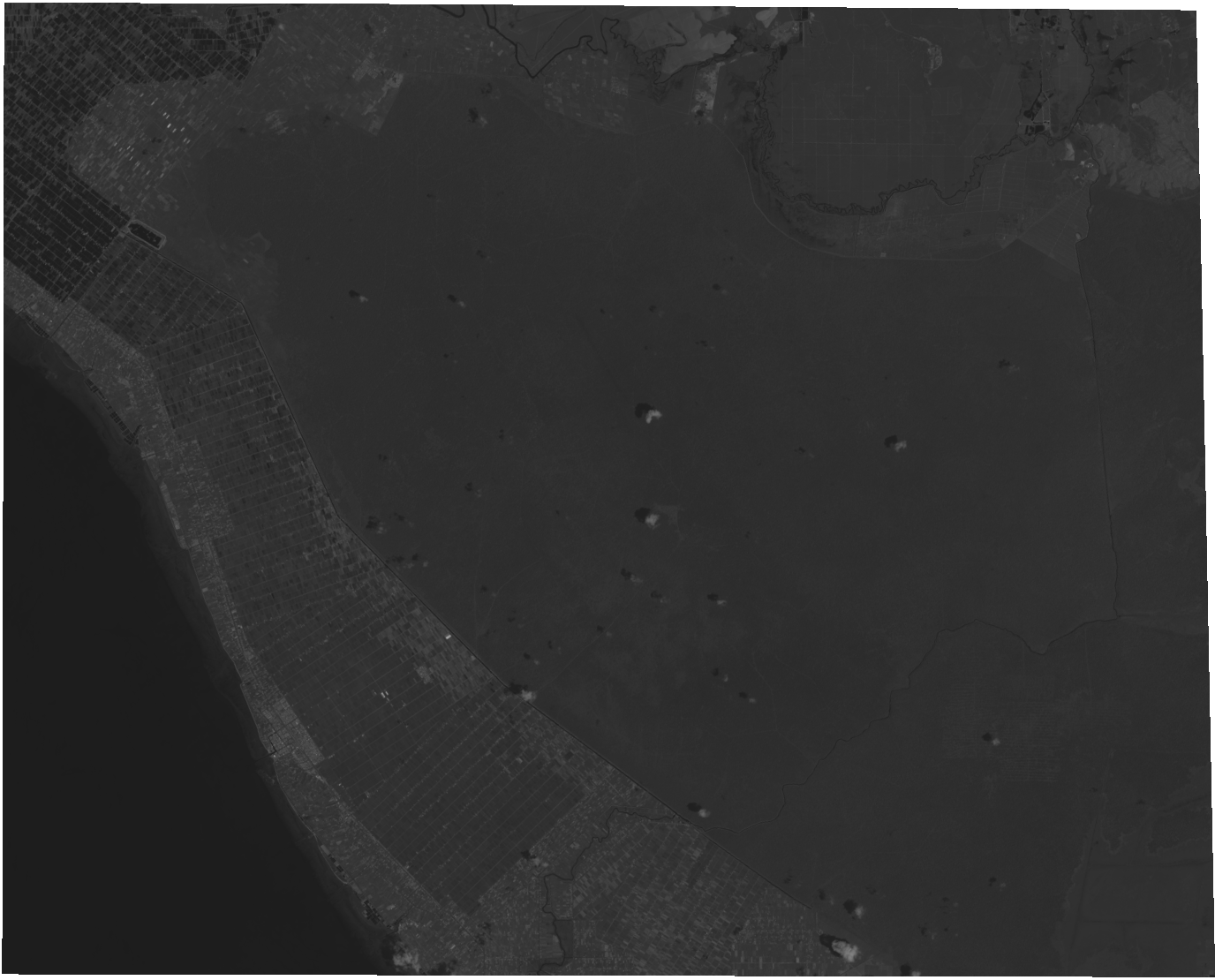

Supplement: S2 File — (ZIP) [file pone.0280187.s002.zip › Landsat2019_clip/LC08_L2SP_127058_20191016_20200825_02_T1_SR_B6.TIF]

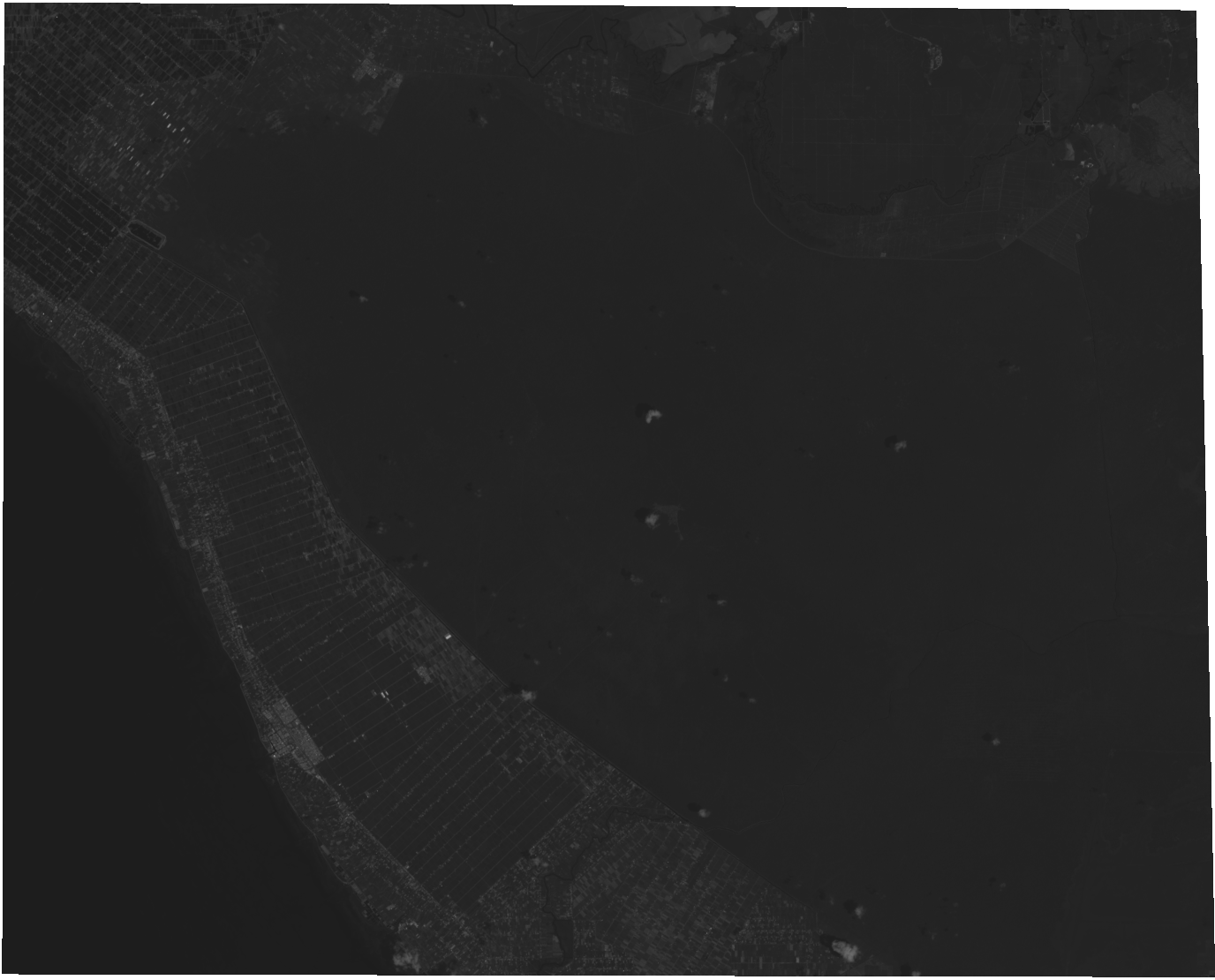

Supplement: S2 File — (ZIP) [file pone.0280187.s002.zip › Landsat2019_clip/LC08_L2SP_127058_20191016_20200825_02_T1_SR_B7.TIF]

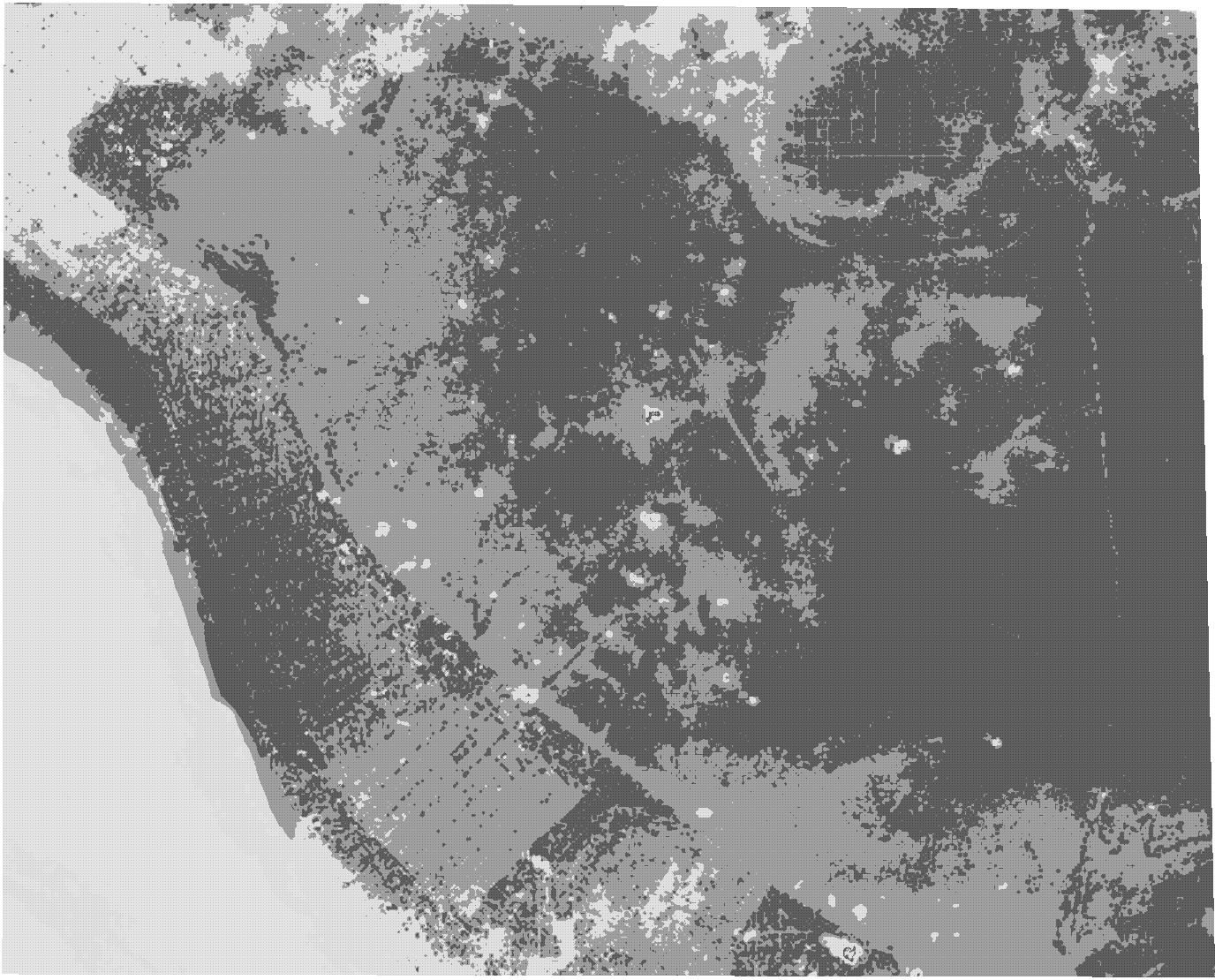

Supplement: S2 File — (ZIP) [file pone.0280187.s002.zip › Landsat2019_clip/LC08_L2SP_127058_20191016_20200825_02_T1_SR_QA_AEROSOL.TIF]

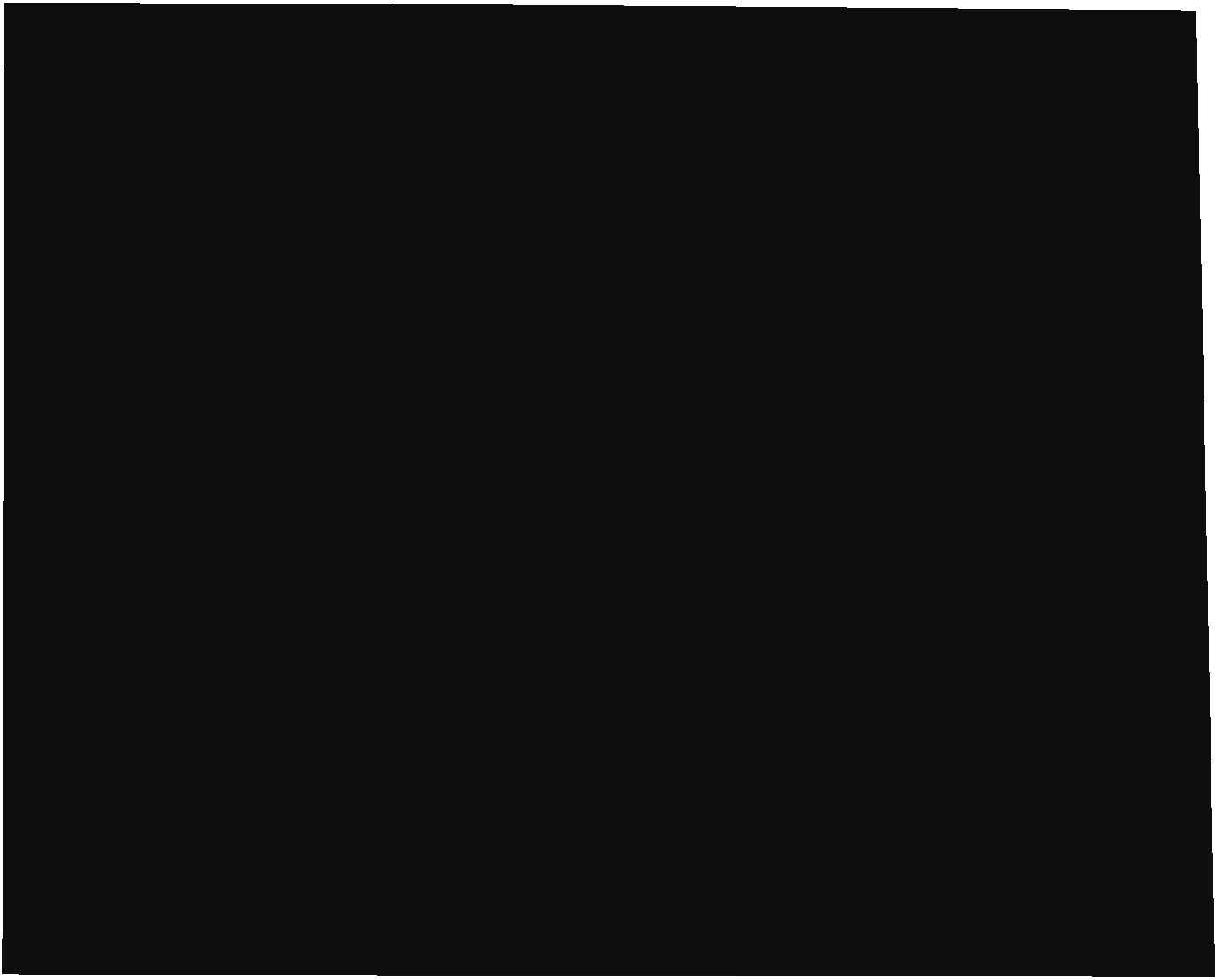

Supplement: S2 File — (ZIP) [file pone.0280187.s002.zip › Landsat2019_clip/LC08_L2SP_127058_20191016_20200825_02_T1_ST_ATRAN.TIF]

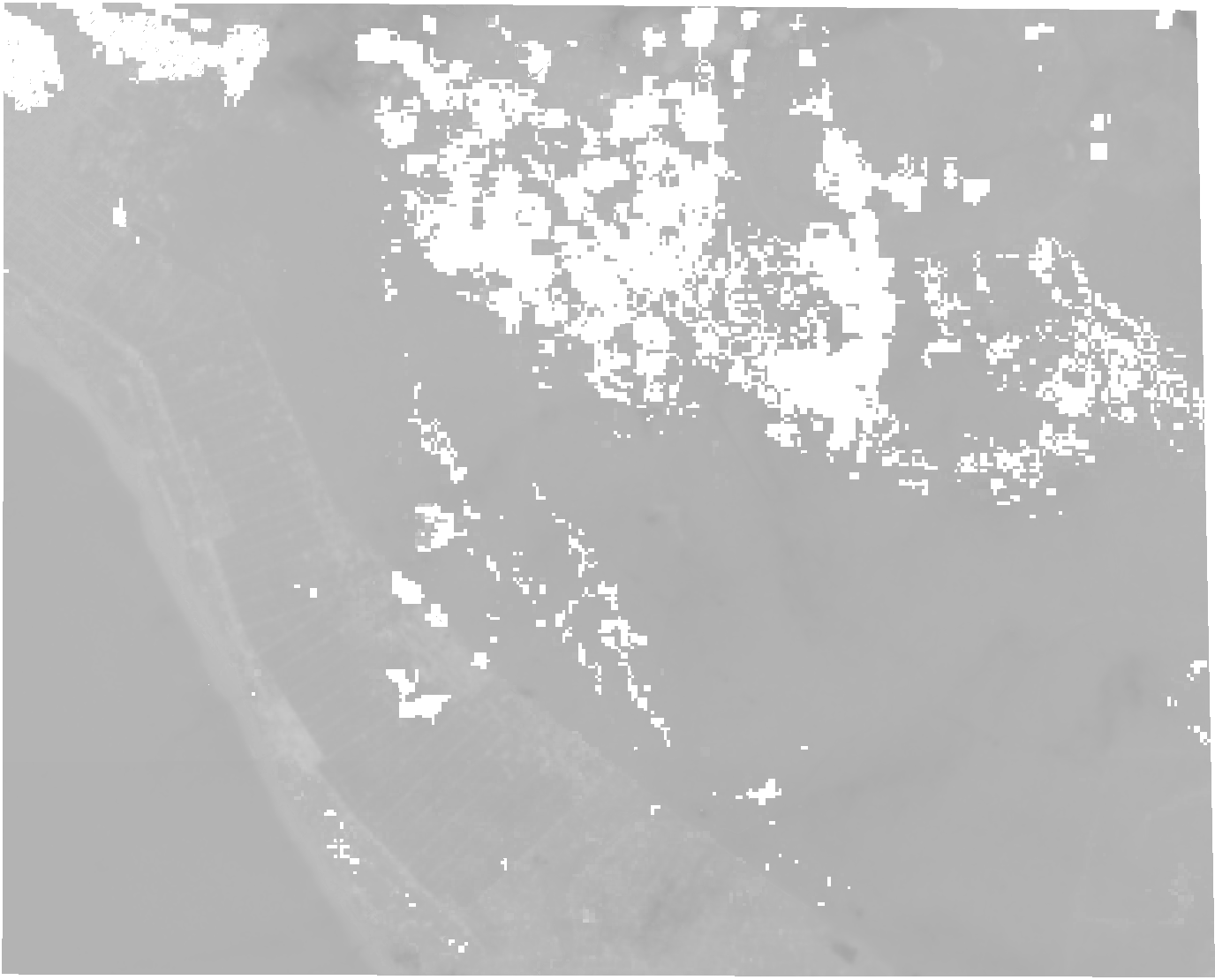

Supplement: S2 File — (ZIP) [file pone.0280187.s002.zip › Landsat2019_clip/LC08_L2SP_127058_20191016_20200825_02_T1_ST_B10.TIF]

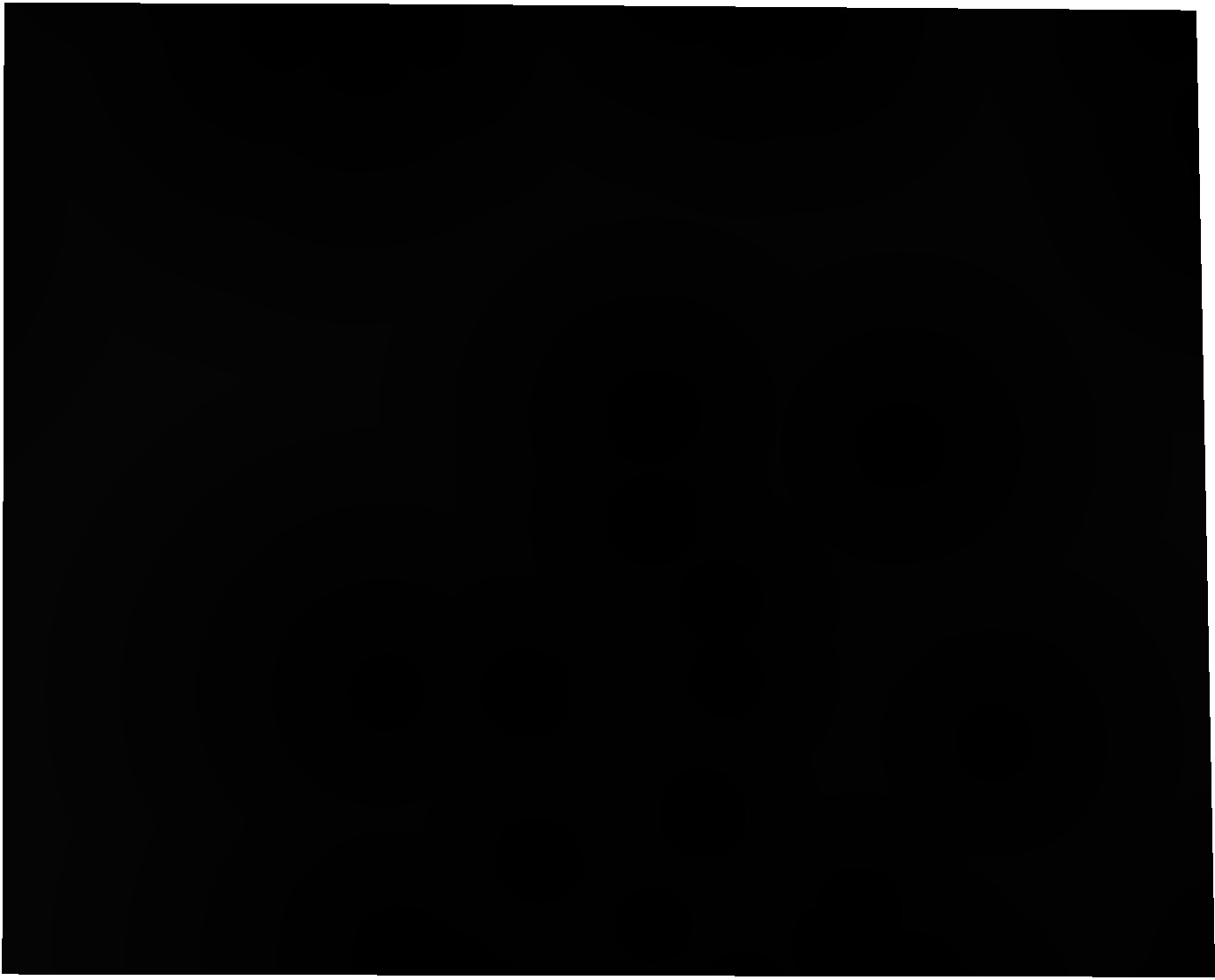

Supplement: S2 File — (ZIP) [file pone.0280187.s002.zip › Landsat2019_clip/LC08_L2SP_127058_20191016_20200825_02_T1_ST_CDIST.TIF]

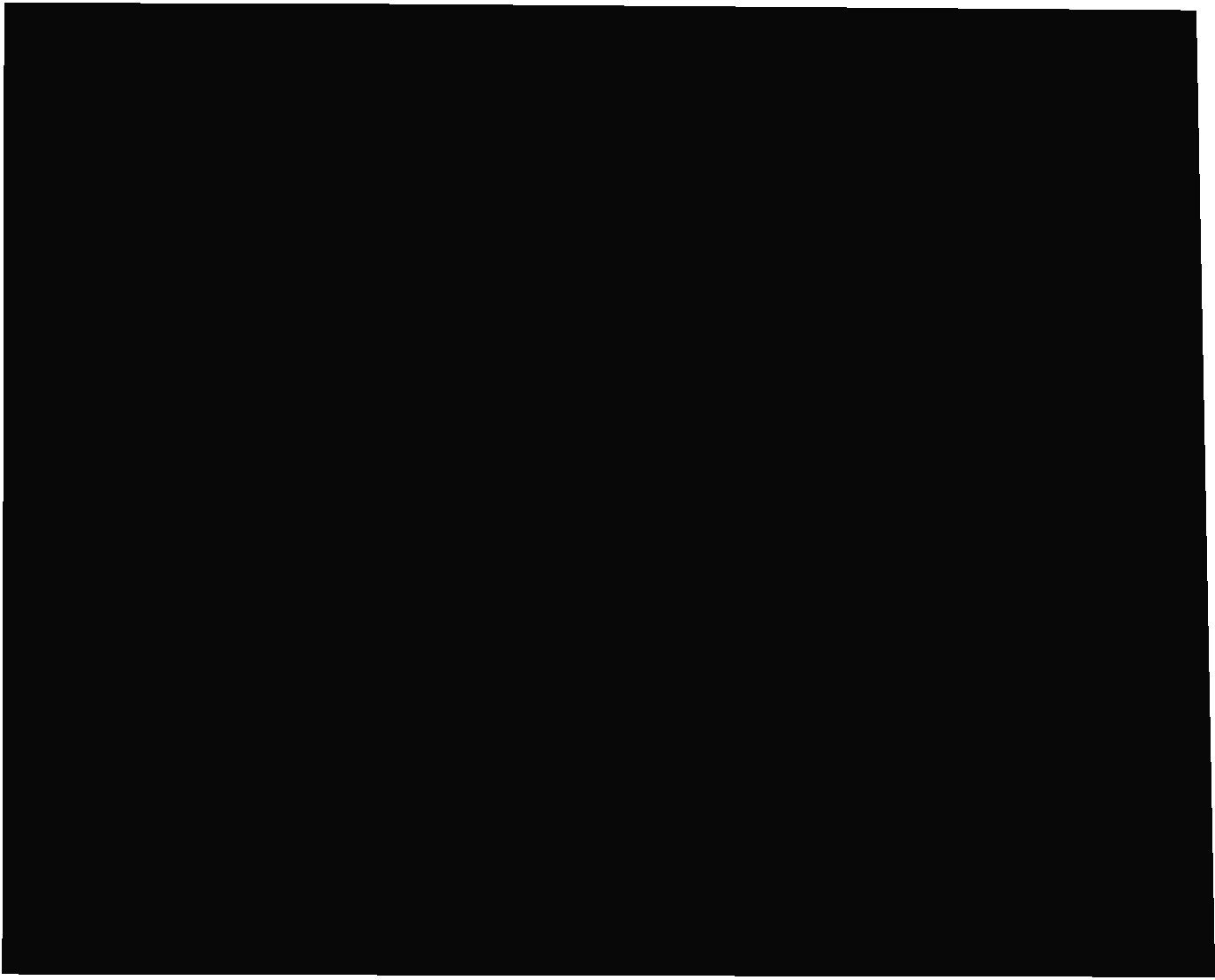

Supplement: S2 File — (ZIP) [file pone.0280187.s002.zip › Landsat2019_clip/LC08_L2SP_127058_20191016_20200825_02_T1_ST_DRAD.TIF]

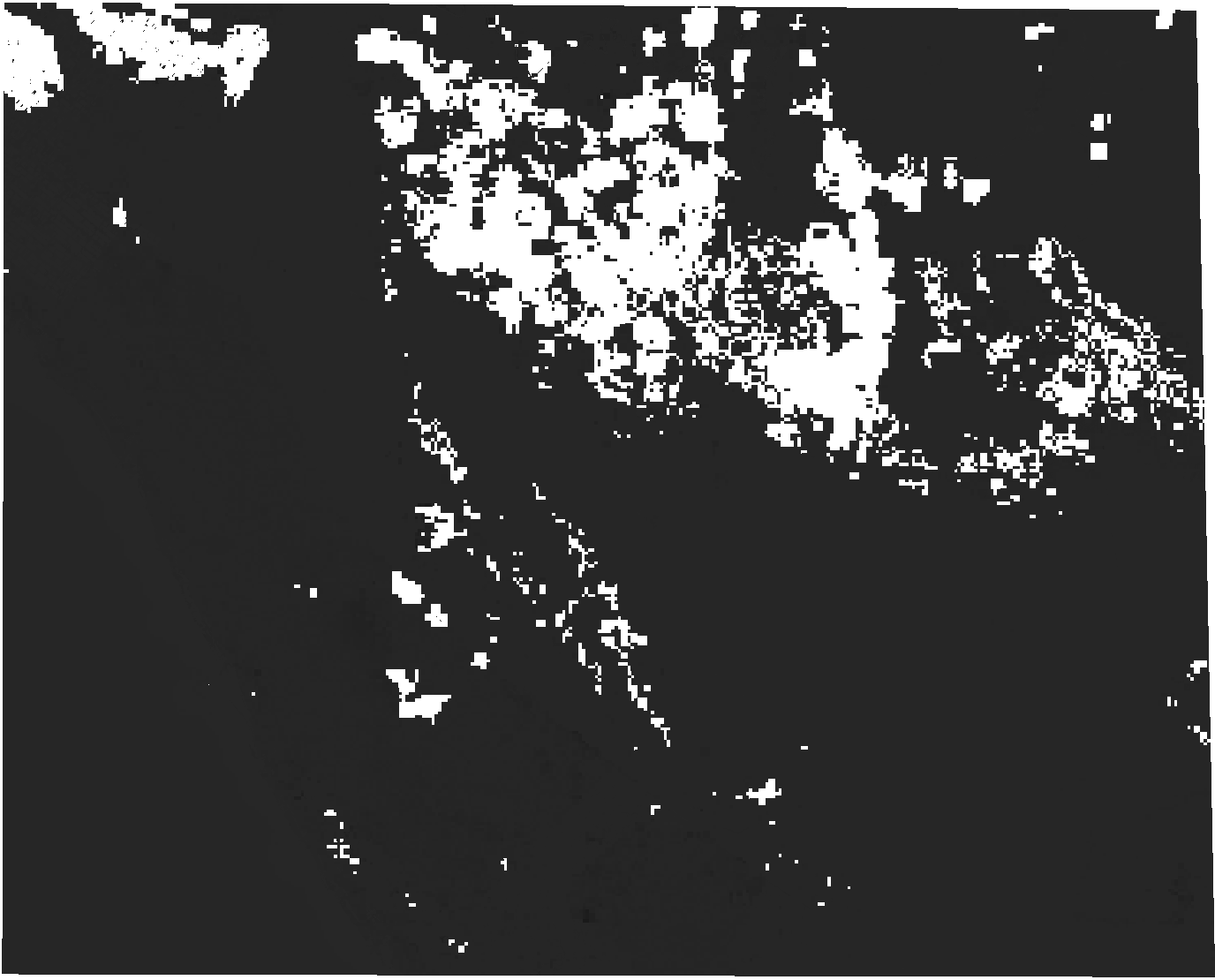

Supplement: S2 File — (ZIP) [file pone.0280187.s002.zip › Landsat2019_clip/LC08_L2SP_127058_20191016_20200825_02_T1_ST_EMIS.TIF]

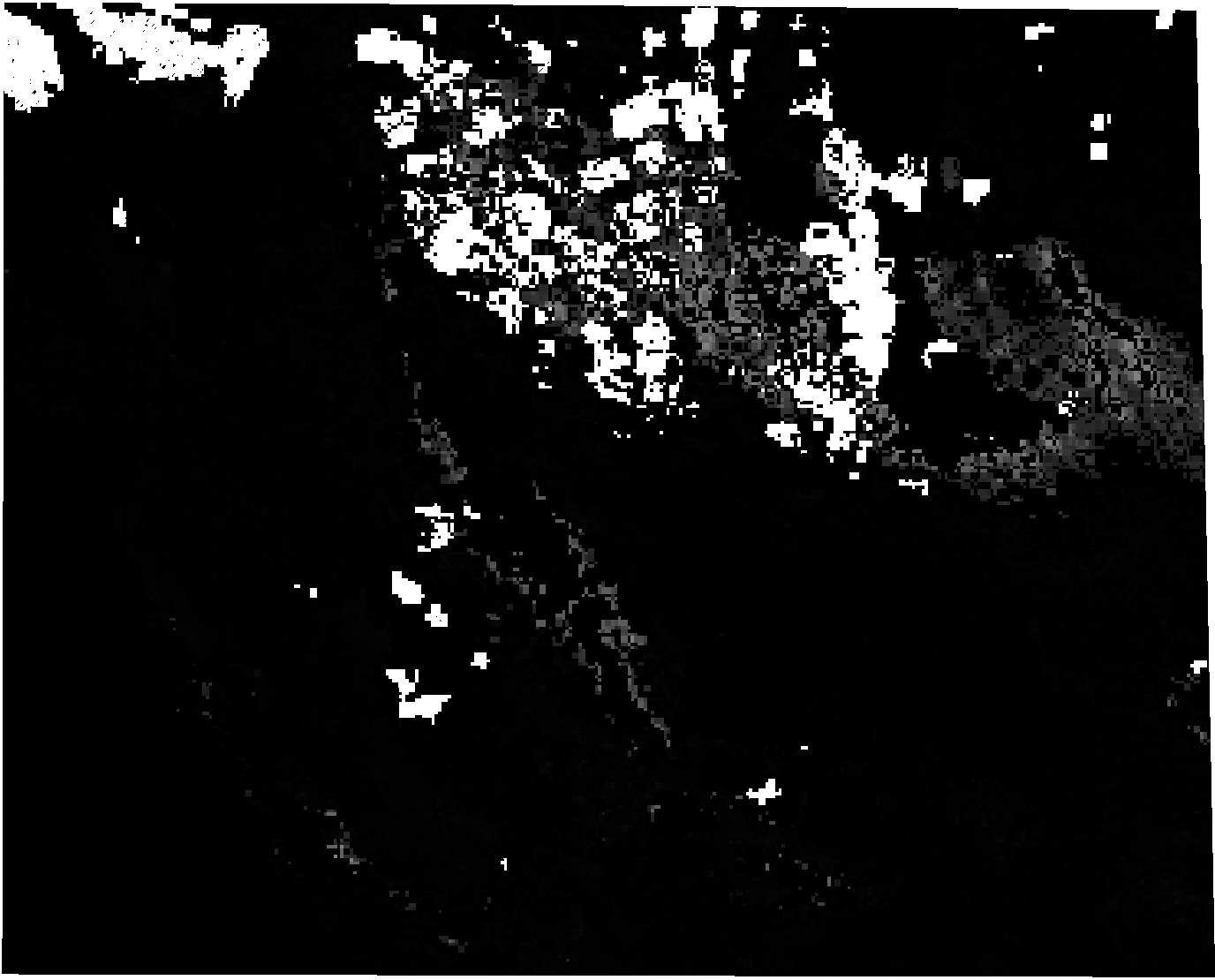

Supplement: S2 File — (ZIP) [file pone.0280187.s002.zip › Landsat2019_clip/LC08_L2SP_127058_20191016_20200825_02_T1_ST_EMSD.TIF]

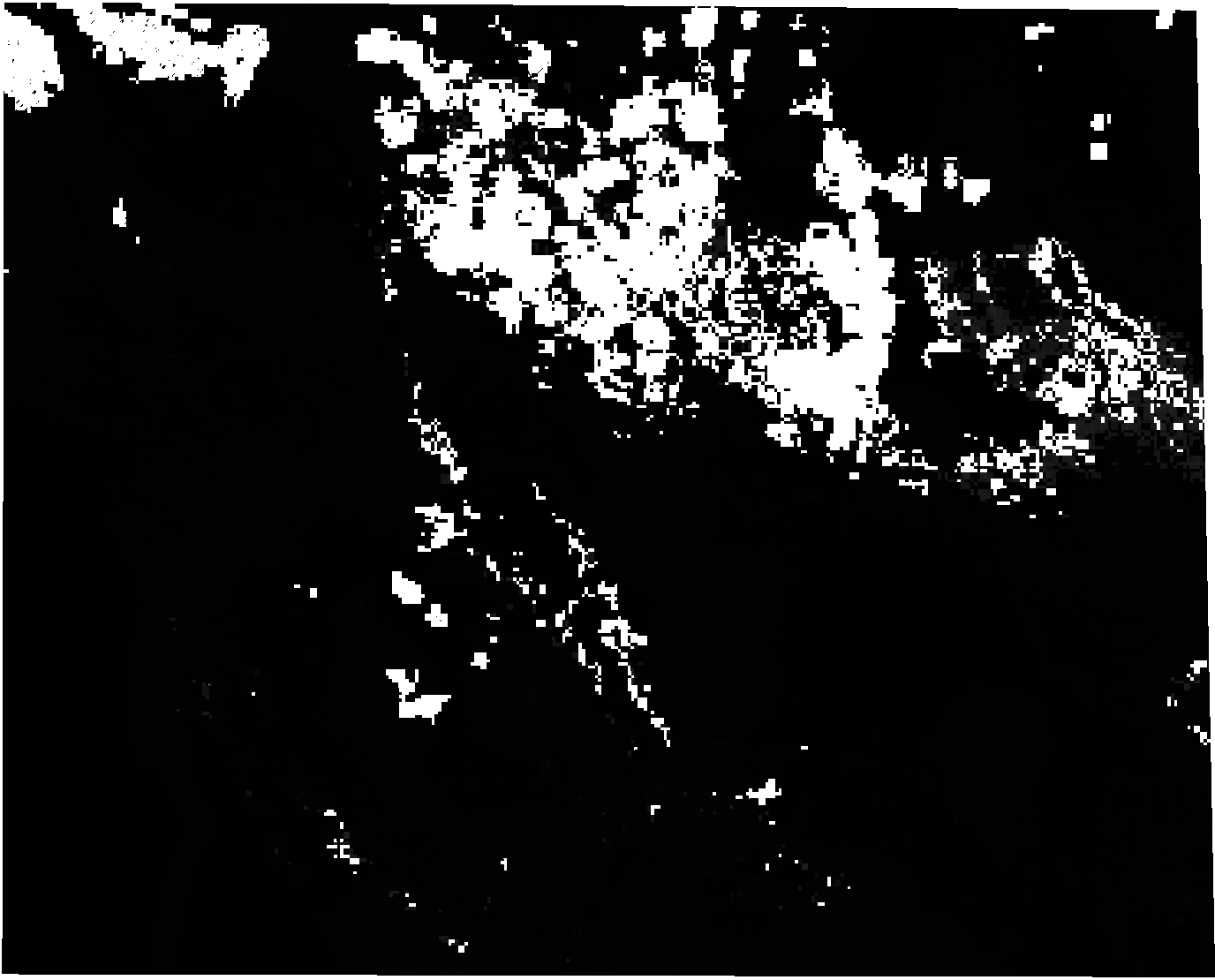

Supplement: S2 File — (ZIP) [file pone.0280187.s002.zip › Landsat2019_clip/LC08_L2SP_127058_20191016_20200825_02_T1_ST_QA.TIF]

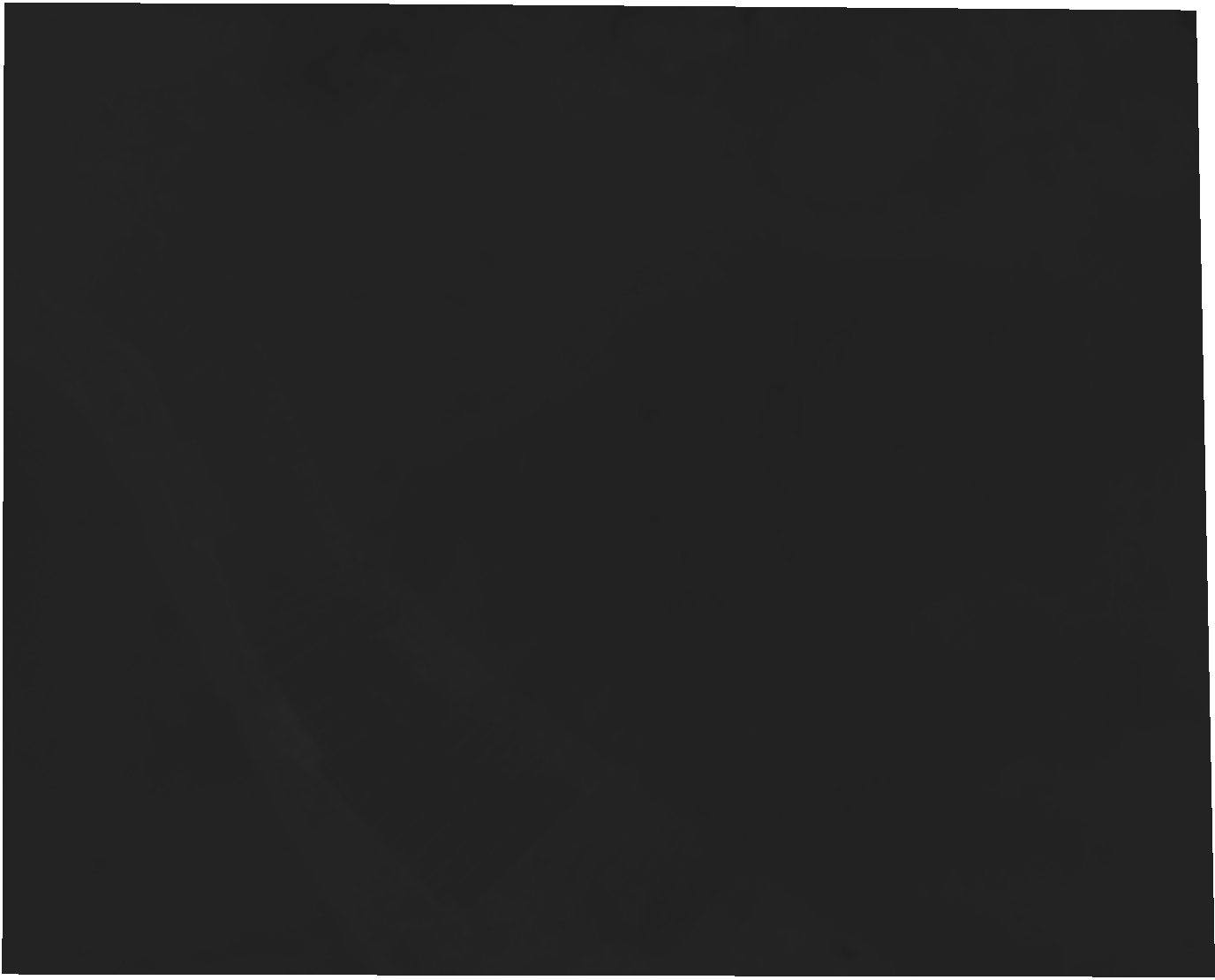

Supplement: S2 File — (ZIP) [file pone.0280187.s002.zip › Landsat2019_clip/LC08_L2SP_127058_20191016_20200825_02_T1_ST_TRAD.TIF]

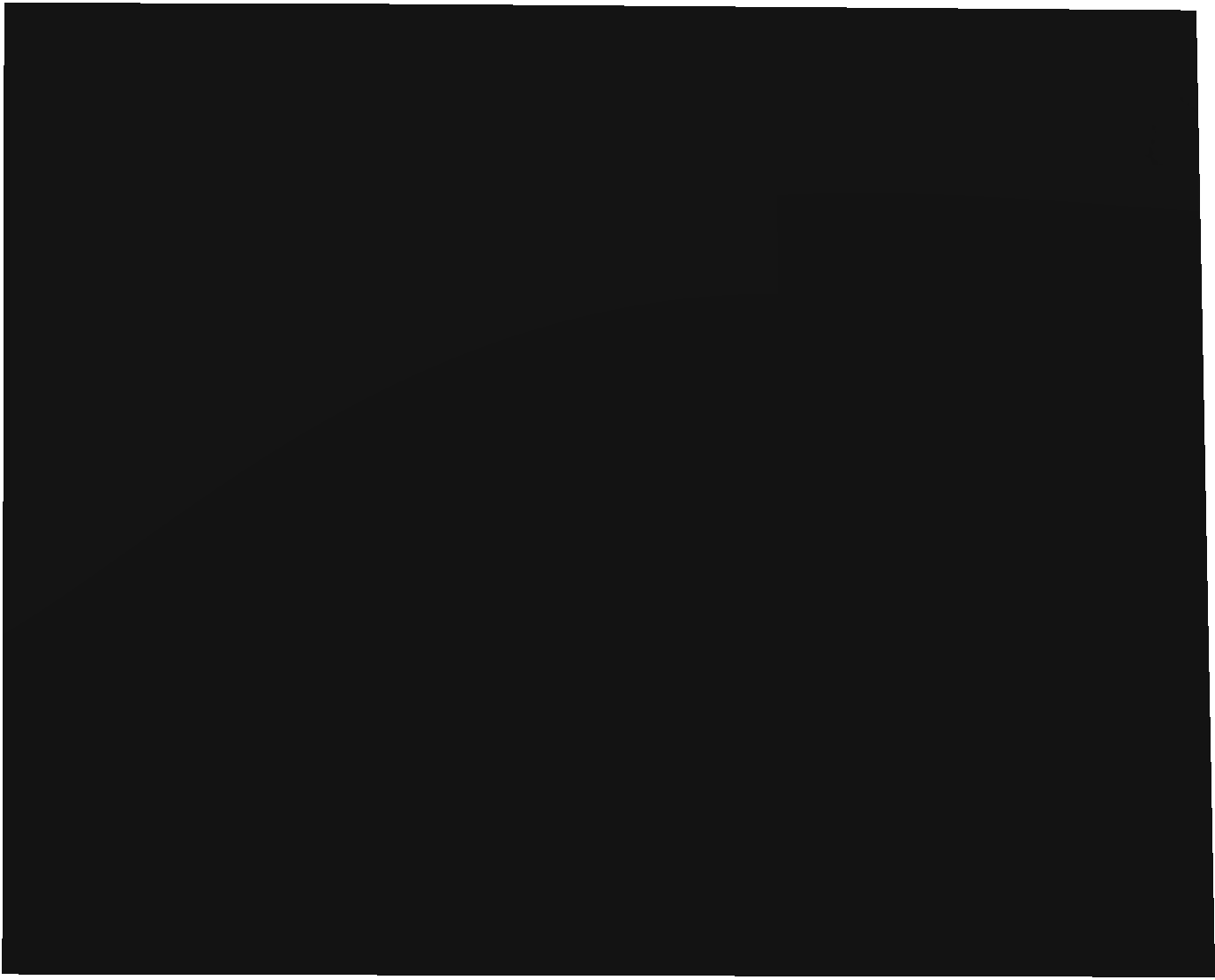

Supplement: S2 File — (ZIP) [file pone.0280187.s002.zip › Landsat2019_clip/LC08_L2SP_127058_20191016_20200825_02_T1_ST_URAD.TIF]
